# Scaffold Hopping Provides Liver X Receptor Agonist With Preference for the β Subtype

**DOI:** 10.1002/cmdc.70509

**Published:** 2026-10-07

**Authors:** Annette Kärcher, Fiona L. Motel, Felix F. Lillich, Astrid Kaiser, Ewgenij Proschak, Pascal Heitel

**Affiliations:** ^1^ Institute of Pharmaceutical Chemistry Goethe University Frankfurt Frankfurt am Main Germany

**Keywords:** drug design, drug discovery, functional selectivity, medicinal chemistry, spiro compounds

## Abstract

The liver X receptor is a promising target for diseases with dysregulated cholesterol levels. In addition to enhanced reverse cholesterol transport, however, LXR activation induces hepatic lipogenesis. Since the subtype LXRα is dominant in the liver, one strategy to overcome this is the development of LXRβ‐selective agonists. We optimized the reported compound **2** with efficacy‐based preference for LXRβ by scaffold hopping to enhance potency and selectivity. Among the four different scaffolds, which were obtained from computer‐assisted design and subsequent synthesis, compound **7** emerged as the most promising scaffold. The spiro[indan‐1,4′‐piperidine] scaffold of **7** demonstrated superior pharmacokinetic and pharmacodynamic properties, warranting its further investigation towards a pharmacological tool compound.

## Graphical abstract

A scaffold hopping strategy was pursued to enhance the binding pocket engagement and β‐subtype selectivity of liver X receptor agonist **2**. The resulting spirocyclic lead compound, (*S*)‐**7**, demonstrates superior potency, enhanced potency‐based selectivity for LXRβ, and improved microsomal stability over the existing scaffold.

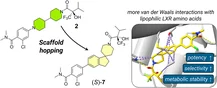

## 1 Introduction

As ligand‐activated transcription factors, liver X receptors (LXRs) are key regulators in cholesterol and lipid homeostasis, immune function, and energy metabolism [1]. They form heterodimers with the retinoid X receptor (RXR) in the nucleus and bind to response elements, which are specific DNA sequences within the promoter of their target genes [2]. In the absence of ligands, heterodimers are constitutively bound to corepressors, resulting in transrepression [3]. The binding of agonists leads to subtle conformational changes, releasing the corepressors and enabling the recruitment of coactivators, which induce target gene transcription [4]. There are two subtypes, LXRα (NR1H3) and LXRβ (NR1H2), sharing ≥74% sequence homology [5]. Both subtypes have the same biological function but differ in their expression pattern. In human, LXRα is abundant in metabolically active tissue and cells such as liver, intestine, adipose tissue, and macrophages, whereas LXRβ is expressed ubiquitously with a higher expression in the central nervous system (CNS) than LXRα [6, 7]. LXR activation enhances reverse cholesterol transport (RCT) from peripheric tissue via macrophages to the liver by increasing the expression of cholesterol‐transporting target genes, including apolipoprotein E (ApoE) and the ATP‐binding cassette transporters A1, G1, G5, and G8 (ABCA1/G1/G5/G8) [8]. Furthermore, LXRs are master regulators of hepatic de novo lipogenesis (DNL) by upregulating sterol regulatory element‐binding protein 1c (SREBP1c) and fatty acid synthase (FAS). Since LXRα is the dominant subtype in the liver, several attempts have been made to develop LXRβ‐selective agonists, including **1**–**4** (Figure 1) [9, 10, 11, 12, 13, 14, 15, 16]. In theory, such subtype‐selective ligands could increase cholesterol excretion while avoiding to stimulate DNL and to promote steatotic effects. Although some of them were tested in clinical trials for atherosclerosis and atopic dermatitis, none of them moved beyond clinical phase II [17, 18, 19].

**FIGURE 1:**
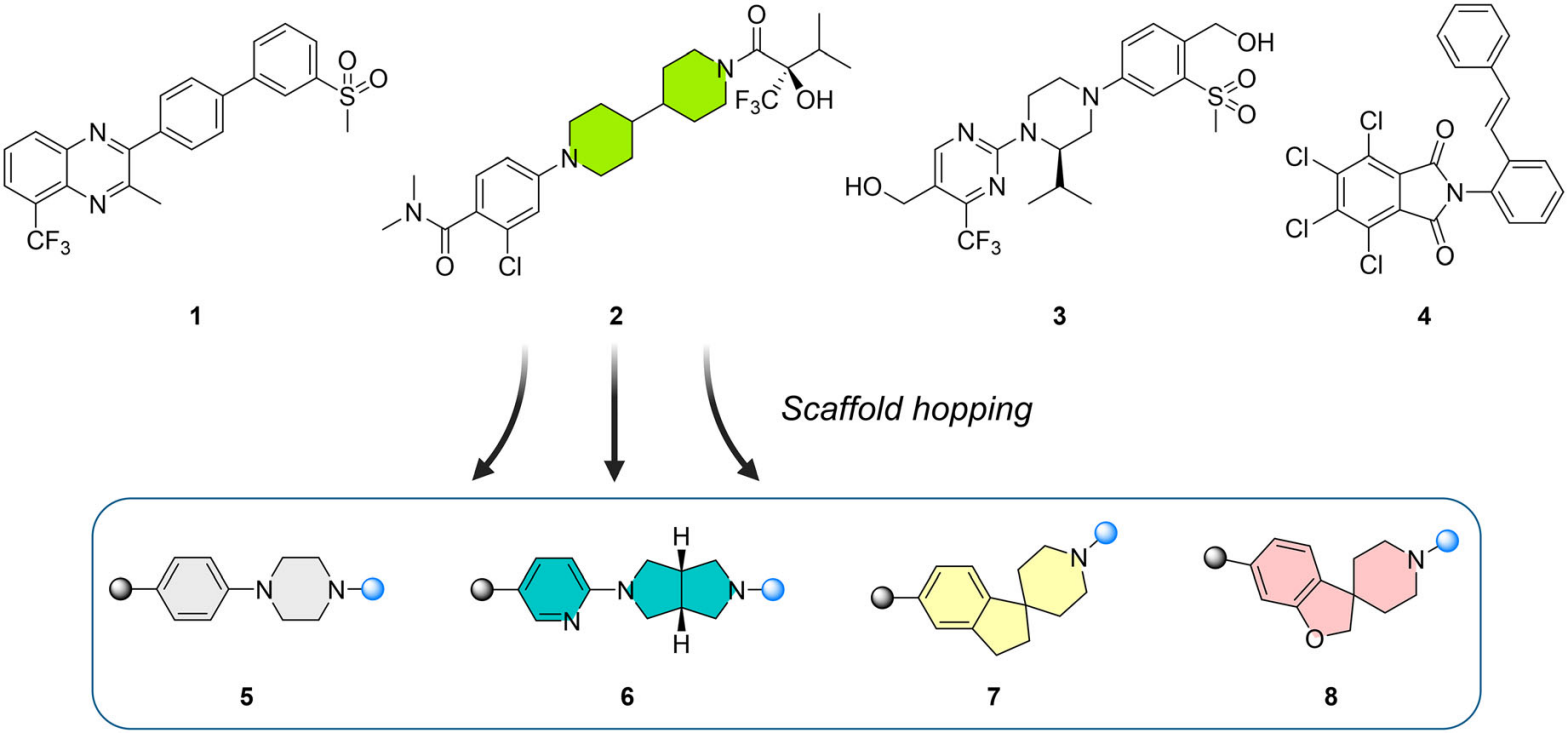
Chemical structure of selected liver X receptor agonists with preference for the β subtype (**1**–**4**) and molecular designs (**5**–**8**) for the scaffold hopping approach.

In 2016, Stachel et al. developed liver X receptor (LXR) β agonist **2** for the potential treatment of Alzheimer's disease (AD) with moderate efficacy‐based selectivity over the LXRα subtype, i.e., the maximum activation on LXRα was lower (31%) than on LXRβ (104%), whereas the potencies were balanced (0.83/0.42 µM) in transactivation assays [12]. In rodent AD models, it increased cerebral levels of the cholesterol‐transporting ApoE and ABCA1. It also demonstrated a propensity to decrease diethylamine‐soluble amyloid‐β levels, but this effect did not reach statistical significance. Although compound **2** did not elevate triglyceride levels in the liver and plasma of rhesus monkeys, significant elevations in low‐density lipoprotein (LDL) cholesterol have been observed at plasma concentrations above the in vitro EC_50_ for LXRα [12]. Furthermore, the compound accumulated at a dose of 20 mg/kg, so that **2** did not qualify for studies beyond preclinical investigations. Despite initially promising results, this study showed that the selectivity profile of LXR agonists needs to be further improved to overcome lipogenic potential.

Since **2** and other reported LXRβ‐selective agonists either did not progress into clinical studies or were discontinued at an early stage, there is a high need for new LXR ligands to explore their therapeutic potential in disorders with cholesterol dysregulation. With compound **2** as a starting point, we used a computer‐assisted scaffold hopping method to develop LXR agonists with improved potency and selectivity for the β subtype.

## 2 Results and Discussion

Based on compound **2** with beneficial physicochemical and pharmacokinetic properties, we wanted to develop new LXRβ‐selective agonists as pharmacological tool compounds [12]. To this end, we employed a computer‐aided scaffold hopping method to improve the interaction of the 4,4′‐bipiperidine core with the ligand‐binding pocket (LBP). An inspection of the cocrystal structures of **2** in LXRα and LXRβ (PDB IDs 5HJS and 5HJP [12]) revealed that the major contributions to binding affinity stem from the ends of the molecule, where the amide oxygen atoms are involved in hydrogen bonds to His421/434 and the amide backbone of Leu316/329 on the other end of the LBP (Figure 2). The 4,4′‐bipiperidine scaffold primarily served as a spacer to facilitate the hydrogen bonds, and we saw potential in this moiety to engage in more lipophilic contacts with LXRs.

**FIGURE 2:**
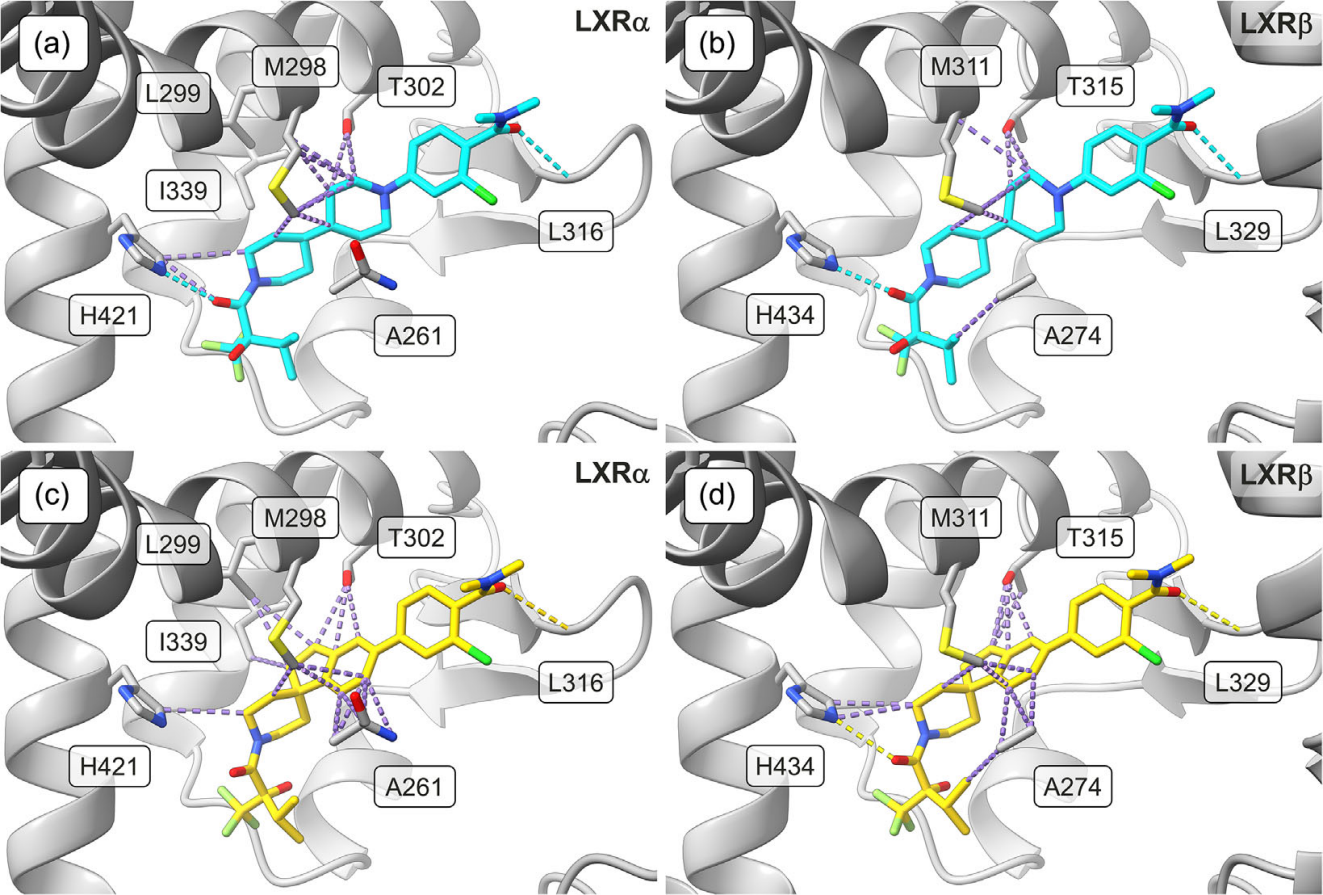
Cocrystal structure of **2** and molecular docking of (*S*)‐**7** into the ligand‐binding pocket of LXRα (PDB ID 5HJS [12]) and LXRβ (PDB ID 5HJP [12]). (a,b) Cocrystal structure of **2** (cyan) in LXRα and LXRβ for comparison. The binding affinity of **2** to LXRs is largely driven by hydrogen bonds (cyan) to His421/434 and Leu316/329. For the sake of clarity, helices 3 and 12 are not displayed and only those van der Waals contacts (purple) that are specific to the molecular scaffold of **2** or (*S*)‐**7** are depicted. (c,d) Molecular docking of (*S*)‐**7** using Autodock Vina [20] indicated binding modes in LXRs similar to the crystal structures of **2**. However, the spirocyclic scaffold of (*S*)‐**7** is involved in more van der Waals interactions (purple) with the hydrophobic central part of the binding pocket than **2**. Furthermore, (*S*)‐**7** only forms a hydrogen bond (yellow) to His421/434 in LXRβ (d) but not in LXRα (c). In total, molecular docking provided a basis for the LXRβ selectivity and improved potency of (*S*)‐**7** observed in biochemical assays. The residue numbering is according to UniProt Q13133 (LXRα) and P55055 (LXRβ).

Using Molecular Operating Environment (MOE; v2022.02), we used the Scaffold Replacement routine to generate suggestions for the replacement of the 4,4′‐bipiperidine scaffold of **2** in the cocrystal structure in LXRβ (PDB ID 5HJP [12]). After visual inspection of the results, we manually selected four different designs based on a combination of low root mean square deviation (RMSD) values and synthetic accessibility (Figure 1). Aside from an *N*‐(2‐methoxyphenyl)piperazine scaffold, all designs were synthesized. Since the sterically demanding methoxy group in the *ortho* position prevented the arylation of the piperazine, we opted instead for the reduced *N*‐phenylpiperazine scaffold **5** to explore the potential of this group.

### 2.1 Synthesis of LXR Ligand Designs

Compounds **5–** **8** were prepared according to Scheme 1, which is based on the reported synthetic route to compound **2** [12]. In the first step, benzoic acid **9** reacted with dimethylamine (**10**) using *O*‐(7‐azabenzotriazol‐1‐yl)‐*N*,*N*,*N*′,*N*′‐tetramethyluronium hexafluorophosphate (HATU) as a coupling reagent to provide amide **11**. This intermediate (**11**) was then borylated with B_2_pin_2_, resulting in **12**, which was used in the next step to introduce the new scaffolds via Suzuki reaction with bromoarenes **13b–** **d**, giving **14b**–**d**. Subsequent deprotection of the Boc group of compounds **14a–** **d** and amide coupling with **15** yielded **16a–** **d**. The final compounds **5–** **8** were obtained by nucleophilic trifluoromethylation using the Ruppert–Prakash reagent.

**SCHEME 1:**
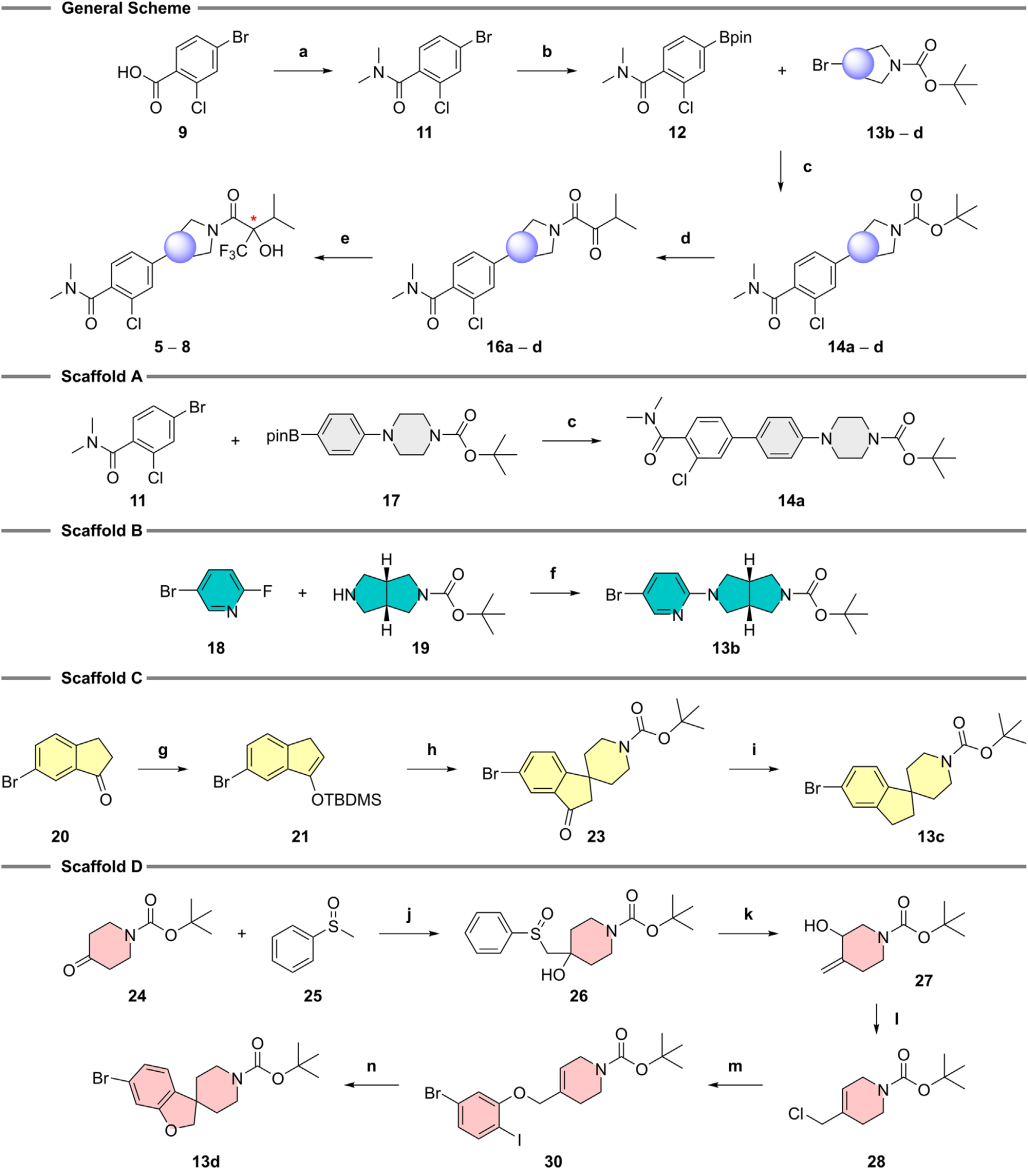
Synthesis of LXR agonist designs **5**–**8**. Reagents and conditions: (a) dimethylamine (**10**), HATU, DIPEA, DMF, rt, 42%; (b) B_2_pin_2_, KOAc, PdCl_2_(dppf), DMSO, 80 °C, 13%; (c) Pd(PPh_3_)_4_, Na_2_CO_3_, 1,4‐dioxane/H_2_O, 80 °C, 54%–84%; (d) (1) TFA, CH_2_Cl_2_, rt, (2) 3‐methyl‐2‐oxobutanoic acid (**15**), PyBOP, DIPEA, DMF, rt, 51%–77%; (e) CF_3_‐TMS, THF, TBAF, rt, 35%–78%; (f) K_2_CO_3_, DMSO, 80 °C, 71%; (g) TBDMSCl, DBU, toluene, 0 °C–rt, 41%; (h) (1) *tert*‐butyl bis(2‐chloroethyl)carbamate (**22**), LiHMDS, THF, −78 °C–rt, (2) TBAF, THF, rt, 64%; (i) (1) KOH, hydrazine hydrate, diethylene glycol, 200 °C, 99%, (2) Boc_2_O, diethylene glycol, MeOH, NEt_3_, rt, 50%; (j) LDA, THF, −78 °C–rt, 39%; (k) KO*t‐*Bu, *t*‐BuOH, 82 °C, 61%; (l) SOCl_2_, 2,6‐lutidine, 0–40 °C, 42%; (m) 5‐bromo‐2‐iodophenol (**29**), K_2_CO_3_. DMF, 70 °C, 69%; (n) 2,4,6‐tris(diphenylamino)‐5‐fluoroisophthalonitrile (**31**), DIPEA, H_2_O/THF, *hν* (*λ *= 405 nm), rt, 68%.

The *N*‐phenylpiperazine scaffold A was introduced from commercially available boronic acid pinacol ester **17** by switching the functional groups for a Suzuki reaction and reacting **17** with bromoarene **11** to form **14a**. The building blocks for the bromoarene scaffolds B–D (**13b–** **d**) were synthesized according to different procedures, with **13b** being available in one step by nucleophilic substitution of 4‐bromo‐2‐fluoropyridine (**18**) with *cis*‐hexahydropyrrolo[3,4‐*c*]pyrrole **19**. The spirocyclic intermediate **13c** was prepared in three steps from 6‐bromo‐indan‐1‐one (**20**), which was first locked into the enol tautomer by the formation of silyl ether **21** to prevent isomerization in the next step [21]. The spiro compound **23** was then obtained from deprotonation of **21** and reaction with *tert*‐butyl bis(2‐chloroethyl)carbamate (**22**), followed by removal of the protecting group using tetra‐*n*‐butylammonium fluoride. Reduction of the carbonyl group in **23** finally yielded **13c**. The second spirocyclic intermediate **13d** was synthesized starting with the nucleophilic addition of (methylsulfinyl)benzene (**25**) to *N*‐Boc‐4‐piperidone (**24**) [22]. The resulting compound **26** provided allyl alcohol **27** by [2,3]‐sigmatropic Mislow–Evans rearrangement through an allyl sulfoxide intermediate. Subsequent conversion with thionyl chloride afforded allyl chloride **28**, which then reacted with 5‐bromo‐2‐iodophenol (**29**) to give ether **30**. Eventually, photocatalyzed radical hydroarylation at 405 nm provided **13d** [23, 24].

### 2.2 Reporter Gene Assay

The obtained designs **5**–**8** were evaluated in a cell‐based reporter gene assay using overexpression of fusion proteins of the Gal4 DNA‐binding domain and the ligand‐binding domain of LXRα or LXRβ, respectively (Table 1). For comparison, we also synthesized compound **2** according to the literature and tested it as a racemate because of minor differences in the potency and efficacy of the enantiomers [12]. In our setting, **2** was less potent (EC_50_ = 10 µM on LXRα and 9.9 µM on LXRβ) than reported in the literature (EC_50_ = 0.83 µM on LXRα and 0.42 µM on LXRβ). Relative to full‐pan LXR agonist T0901317 (**32**), **2** displayed partial agonistic activity on LXRβ (*E*_max_ = 31%) and efficacy‐based selectivity over LXRα (*E*_max_ = 3.3%). Of note, there are considerable differences between the literature transactivation assay systems for LXRα and LXRβ, compromising a direct comparison of the subtypes: LXRα activity was determined in liver carcinoma HepG2 cells with stable LXRβ knockdown (shRNA) and transient transfection of the ABCA1‐based luciferase reporter, whereas for LXRβ, neuroglioma H4 cells were stably transfected with the ABCA1‐based luciferase reporter without further specification of LXRα or LXRβ expression levels [12]. In contrast to our Gal4 hybrids, the literature assays are based on full‐length LXR proteins with native expression levels in HepG2 (LXRα) and H4 (LXRβ) cells, which could explain the observed differences in potency of **2**.

**TABLE 1:** Structure–activity relationship of LXR agonists with different scaffolds. Dose–response curves are shown in Figure S1.

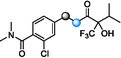							
Compound		LXRα		LXRβ		α/β ratio	
ID	Scaffold	**Gal4** EC_50_, μM (*E*_max_, %^b^)	**fl** a EC_50_, μM (*E*_max_, %^b^)	**Gal4** EC_50_, μM (*E*_max_, %^b^)	**fl** a EC_50_, μM (*E*_max_, %^b^)	**Gal4** EC_50_ (*E*_max_)	**fl** a EC_50_ (*E*_max_)
**32**	—	0.47 ± 0.02 (109 ± 3)	0.021 ± 0.004 (104 ± 3)	0.76 ± 0.10 (135 ± 12)	0.033 ± 0.010 (101 ± 8)	0.61 (0.81)	0.63 (1.0)
(*rac*)‐**2**	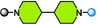	10 ± 1 (3.3 ± 0.2)	0.24 ± 0.03 (64 ± 4)	9.9 ± 1.3 (31 ± 3)	0.097 ± 0.027 (80 ± 6)	1.0 (0.10)	2.5 (0.80)
**5**	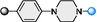	>30	n.t.^d^	1.7 ± 0.1 (1.4 ± 0.1)	n.t.^d^	>18	—
**6**	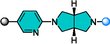	tox.^c^	n.t.^d^	tox.^c^	n.t.^d^	—	—
(*rac*)‐**7**	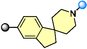	0.70 ± 0.21 (48 ± 6)	n.t.^d^	0.13 ± 0.01 (43 ± 1)	n.t.^d^	5.6 (1.1)	—
(*R*)‐**7**		1.5 ± 0.1 (49 ± 2)	n.t.^d^	0.24 ± 0.04 (46 ± 1)	n.t.^d^	6.2 (1.0)	—
(*S*)‐**7**		0.43 ± 0.08 (27 ± 3)	0.17 ± 0.02 (111 ± 6)	0.16 ± 0.01 (61 ± 1)	0.021 ± 0.005 (95 ± 7)	2.8 (0.44)	7.8 (1.2)
**8**	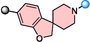	3.8 ± 0.8 (11 ± 1)	n.t.^d^	1.8 ± 0.3 (32 ± 4)	n.t.^d^	2.1 (0.36)	—

a Full‐length.

b Maximum activation relative to 1 μM T0901317 (**32**).

c Not determined due to cytotoxic effects.

d Not tested.

The replacement of the 4,4′‐bipiperidine scaffold of **2** with *N*‐phenylpiperazine (**5**) increased both potency on LXRβ and potency‐based selectivity over LXRα. However, **5** showed very low activation efficacy on LXRβ. Unfortunately, 2‐(*cis*‐hexahydropyrrolo[3,4‐*c*]pyrrolyl)pyridine **6** was cytotoxic at a 10 µM concentration. In contrast, both spirocyclic scaffolds **7** and **8** were more potent than the parent compound **2**. Spiro[indan‐1,4′‐piperidine] **7** displayed the highest potency on LXRβ (EC_50_ = 0.13 µM) with a 78‐fold lower EC_50_ than that of **2**. While the activation efficacy was balanced on both LXRs, **7** demonstrated a potency‐based selectivity for LXRβ with an α/β ratio of 5.6. The 2*H*‐spiro[benzofuran‐3,4′‐piperidine] motif **8** could not match the potency of **7**, but it was preferential for LXRβ both in terms of potency and maximum efficacy. In the next step, we separated the enantiomers of the most potent scaffold **7** by chiral high‐performance liquid chromatography (HPLC) and determined the absolute configuration of (*R*)‐**7** by single‐crystal X‐ray diffraction. Compared to the racemic mixture, (*R*)‐**7** was slightly less potent on both LXR subtypes but retained the balanced *E*_max_ profile. The *S* enantiomer was very potent with a preference for LXRβ (EC_50_ = 0.16 µM, *E*_max_ = 61%) over LXRα (EC_50_ = 0.43 µM, *E*_max_ = 27%) concerning both potency and maximum activation. Starting from compound **2**, the scaffold‐hopping approach was very successful and provided access to the optimized compound (*S*)‐**7**, which almost increased the potency by two orders of magnitude.

### 2.3 Molecular Docking

To rationalize the results of the reporter gene assay, we docked (*S*)‐**7** into the LBD of LXRα (PDB 5HJS) and LXRβ (5HJP) and compared the predicted binding modes to the cocrystallized compound **2** (Figure 2). The key interactions of **2** with LXRs are two hydrogen bonds at the opposite ends of the molecule to His421/434 (LXRα/β) and the amide backbone of Leu316/329. The central part of the LBP is characterized by hydrophobic amino acid residues, and the bipiperidine scaffold of **2** only shows few van der Waals interactions in this region. There is little difference between the binding modes in LXRα and LXRβ, so that the X‐ray crystal structures do not provide a rationale for the observed subtype selectivity [12]. The highest‐ranked docking poses of (*S*)‐**7** in both LXR subtypes generally align well with the binding mode of **2**, thus confirming the validity of the scaffold hopping approach. However, (*S*)‐**7** displays significantly more van der Waals interactions with the hydrophobic central part of the LBP via its spirocyclic scaffold, occupying a larger 3D volume than the scaffold of **2**. In particular, the amino acid residues of Ala261, Leu299 and Ile339 in LXRα are exclusively addressed by (*S*)‐**7**. In the β subtype, the decisive amino acids Ala274, Thr315, and His434 are involved in the stabilization of the protein–ligand complex. This might explain the higher potency of (*S*)‐**7**. Moreover, the distance of the 2‐hydroxy‐3‐methyl‐2‐(trifluoromethyl)butanamide oxygen atom to His421 in LXRα is too large (3.9 Å), whereas a hydrogen bond to His434 can form in LXRβ. Interestingly, Stachel et al. reported some conformational flexibility of this terminal moiety in the LXRα‐bound state, which was reflected in weak electron density [12]. In our docking study, we observed two low‐energy binding modes (docking score pose 1: −13.5 ± 0.1; pose 2: −13.4 ± 0.1) in LXRα with approx. 180° rotation around the C─C bond between the carboxamide carbon and tertiary alcohol carbon atoms, but only one in LXRβ (Figure S2). Altogether, the docking study provided some insights into the LXRβ selectivity of (*S*)‐**7**, but these ultimately require experimental confirmation by cocrystallization.

### 2.4 In Vitro Pharmacological Characterization

With obtaining compound (*S*)‐**7** from scaffold optimization of **2**, we next wanted to investigate the pharmacological potential of this compound. In the first step, we profiled the optimized compound in full‐length LXR reporter gene assays, in which we overexpressed the respective LXR subtype and read out LXR activation by using a luciferase reporter with an ABCA1 promoter (Figure 3a). Compound **2** was characterized as a partial agonist on both LXRs, with EC_50_ values of 0.24 ± 0.03 µM (LXRα) and 0.097 ± 0.027 µM (LXRβ). In contrast to the Gal4 hybrid reporter gene assay, **2** demonstrated high efficacy on both subtypes (*E*_max_ = 64% ± 4% relative to full agonist **32** on LXRα and 80% ± 6% on LXRβ) with little difference between LXR subtypes. With *E*_max_ values of 111% ± 6% (LXRα) and 95% ± 7% (LXRβ), (*S*)‐**7** was more effective and also more potent (EC_50_ = 0.17 ± 0.02 µM on LXRα and EC_50_ = 0.021 ± 0.005 µM on LXRβ) than **2**. Furthermore, (*S*)‐**7** had a higher potency‐based selectivity for LXRβ than **2** (α/β ratio of 7.8 vs. 2.5). For a better comparison of the results from full‐length (fl) and Gal4 hybrid LXR reporter gene assays, we validated these assays with reference LXR agonists T0901317 (**32**) and LXR‐623 (**33**) [25, 26]. Both **32** and **33** achieved higher potency on LXRs in the full‐length assay system (**32**: Gal4‐LXRα EC_50_ = 0.47 µM, Gal4‐LXRβ 0.76 µM, fl‐LXRα 0.021 µM, fl‐LXRβ 0.033 µM; **33**: Gal4‐LXRα EC_50_ = 1.6 µM, Gal4‐LXRβ 1.4 µM, fl‐LXRα 0.063 µM, fl‐LXRβ 0.15 µM), which is in good agreement with literature data (Figure S4) [25, 26, 27]. The EC_50_ values in the full‐length assay were typically 10–20 fold lower than in the Gal4 hybrid setting, and the shift in potency was exceptionally high for **2** on LXRβ (102‐fold). Since the Gal4 hybrid assays are independent of specific LXR target genes, this could indicate that **2** is a selective modulator of the LXR target gene ABCA1.

**FIGURE 3:**
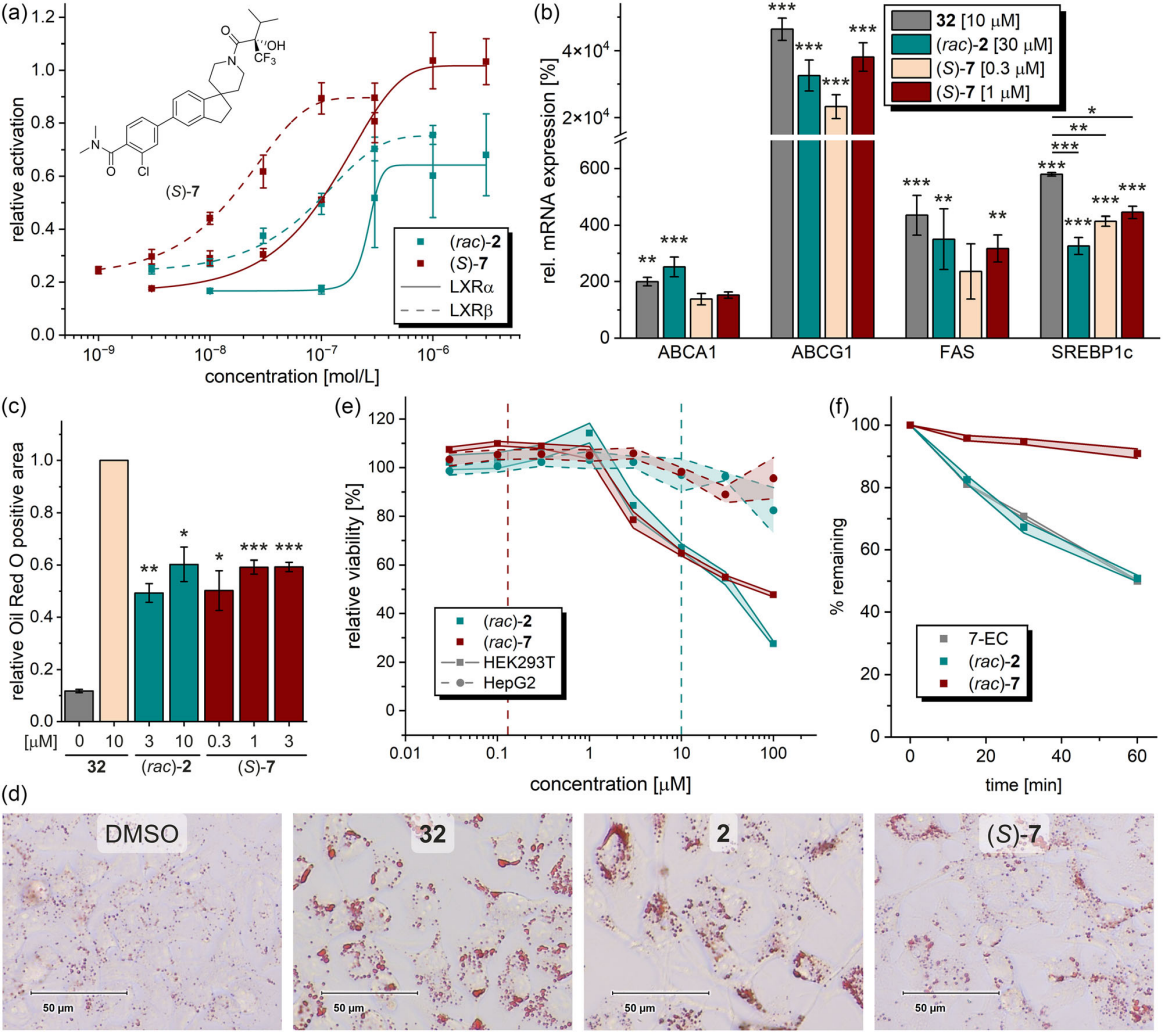
In vitro characterization of the optimized LXR agonist **7**. (a) Full‐length LXR reporter gene assay in HEK293T cells. *N* ≥ 3. (b) Quantification of LXR target genes in HepG2 cells. Both **2** and (*S*)‐**7** robustly induced expression of target genes involved in cholesterol metabolism (ABCA1, ABCG1) and DNL (FAS, SREBP1c). Data are the mean ± SEM of *N* = 4; **p < 0.01, ***p < 0.001 (ANOVA with *post hoc* Bonferroni test against 0.1% DMSO or as indicated by the bars). Full agonist T0901317 (**32**) was tested for comparison. (c,d) Lipogenesis assay. Incubation of HepG2 cells with pan LXR full agonist T0901317 (**32**) for 72 h led to a substantial accumulation of lipids that were stained with Oil Red O. In comparison to **32**, both the parent compound **2** and (*S*)‐**7** displayed less lipogenic potential. Data are the mean ± SEM of *N* = 3; *p < 0.05, **p < 0.01, ***p < 0.001 (one‐sided *t*‐test against 0.1% DMSO). The images shown in (d) are representative details from the full images that can be found in Figure S3. (e) In a cytotoxicity assay, **2** and (*rac*)‐**7** did not influence the metabolic activity of HepG2 cells but induced cytotoxicity to HEK293T cells in the micromolar concentration region. While these concentrations are well above the EC_50_ value of (*S*)‐**7** in the Gal4 reporter gene assay (red dashed line), compound **2** showed partially toxic effects at its EC_50_ value (green dashed line). (f) Metabolic stability of **2** and (*rac*)‐**7** in rat liver microsomes. After 60 min, (*rac*)‐**7** was almost recovered completely, whereas approximately half of **2** had been metabolized. 7‐Ethoxycoumarine (7‐EC) served as internal standard. Data are the mean ± SEM of *N* = 3.

To assess if the increased potency‐based selectivity of (*S*)‐**7** goes hand in hand with less or abolished hepatic lipogenesis, two additional assays were carried out. First, the transcription of LXR target genes was quantified by quantitative polymerase chain reaction (qPCR), and second, accumulation of triglycerides was quantified by Oil Red O staining of HepG2 cells. Both **2** and (*S*)‐**7** induced the expression of the cholesterol‐transporting LXR targets ABCA1 and ABCG1, although the effect did not reach statistical significance by ANOVA for (*S*)‐**7** and ABCA1 (Figure 3b). By contrast, all LXR agonists, including the reference full agonist T0901317 (**32**), induced ABCG1 transcription sharply. Both **2** and (*S*)‐**7** increased the expression of LXR targets involved in DNL (FAS and SREBP1c), but for SREBP1c, the effect size was smaller than for full agonist **32** (one‐way ANOVA followed by Bonferroni test). While we observed the same trend for FAS, the difference between full agonist **32** and **2** or (*S*)‐**7** was not statistically significant. Overall, the target gene expression was in good agreement with the reporter gene assays, where **2** and (*S*)‐**7** demonstrated partial LXR agonism compared to full agonist **32**. Furthermore, (*S*)‐**7** showed a tendency towards a dose‐dependent effect size at 0.3 and 1 µM, approximately corresponding to the Gal4 EC_80_ values on LXRβ and LXRα, respectively. Therefore, the Gal4 assays appear to better reflect the pharmacodynamic properties of **2** and (*S*)‐**7** than the full‐length reporter gene assay.

The induction of hepatic lipogenesis via SREBP1c is an undesired activity of LXR agonists for the potential use in disorders with dysregulated cholesterol levels because it enhances liver cholesterol. We therefore performed an additional assay in HepG2 cells to assess the lipogenic potential of (*S*)‐**7** (Figure 3c,d). After 72 h treatment with LXR full agonist T0901317 (**32**, 10 µM), the cells showed a massive accumulation of neutral lipids that were stained with Oil Red O and quantified. Consistent with the increased transcription of SREBP1c, **2,** and (*S*)‐**7** also enhanced lipid formation at concentrations corresponding to approximately the EC_80_ values in the Gal4 hybrid reporter gene assay. However, the amount of lipids was only 49%–60% of that induced by the full agonist **32**. Notably, both the quantification of target gene expression and lipogenesis were evaluated in HepG2 cells, which are derived from liver carcinoma and do not reflect the physiological or pathophysiological situation perfectly. Among other factors, this includes the fact that the LXR subtype expression profile differs from the physiological LXRα dominance in the liver. In a previous study, we showed that HepG2 cells express both LXR subtypes to a similar extent [28]. Further in vivo studies are required to investigate whether (*S*)‐**7** has a better DNL profile than **2**, particularly since **2** tended to accumulate at higher doses in vivo [12].

For this reason, we examined the pharmacokinetic properties of the optimized compound. Both **2** and **7** had a similar toxicity profile and did not affect the viability of HepG2 cells after 24 h even at a 100 µM concentration (Figure 3e). However, at 3 µM or higher concentrations, they partially reduced the viability of HEK293T cells dose‐dependently. The significantly higher potency of **7** in the Gal4 hybrid reporter gene assay contributes to a better safety profile and indicates that the in vitro therapeutic window for **7** is superior to that for **2**. Additionally, the spirocyclic compound **7** was very stable in rat liver microsomes after 60 min, while after the same time, approximately half of bipiperidine **2** was already metabolized (Figure 3f). With these favorable pharmacokinetic properties and the gain in potency and potency‐based selectivity, (*S*)‐**7** qualifies for further investigations.

## 3 Conclusion

LXR agonist **2** is moderately potent but functionally selective for LXRβ due to its higher transactivation efficacy compared to LXRα. Since increased cholesterol levels were observed in preclinical studies, we improved LXR ligand **2** by scaffold hopping to strengthen the interaction with the lipophilic central part of the LBP. The key step in the synthesis of the optimized spiro[indan‐1,4′‐piperidine] scaffold was the regioselective generation of the spiro carbon atom by using an enol silyl ether to avoid the isomerization of the carbon double bond position. The resulting compound (*S*)‐**7** was very potent with EC_50_ values in the nanomolar region on LXR‐Gal4 hybrids and the full‐length receptors and had a better potency‐based selectivity for LXRβ than that of **2**. Although there is no difference in the absolute cytotoxicity profile of **2** and (*S*)‐**7** on HEK293T cells, (*S*)‐**7** has a significantly superior therapeutic window based on Gal4 EC_50_ values. Together with the high metabolic stability, these results are highly encouraging, but additional experiments are required to assess the lipogenic potential of (*S*)‐**7**. Further improvement in LXRβ selectivity seems to be required to segregate the therapeutic effects from lipogenic side effects; however, this has proven to be very challenging due to the high sequence identity with LXRα. Therefore, tissue‐selective activation appears as a promising strategy to avoid potential adverse effects from systemic LXR activation.

## 4 Experimental Section

### 4.1 Chemistry

#### 4.1.1 General Information

All chemicals and solvents were obtained from commercial sources in reagent grade and used without further purification. TLC was performed using TLC plates (silica gel 60 F254, 0.2 mm, Merck or Alugram Xtra Sil g/UV 0.2 mm, Macherey‐Nagel) with detection under UV light (254 and 366 nm). Preparative column chromatography was performed using Silicagel 60 (Macherey‐Nagel) and solvents of technical grade. Reactions with air‐ or moisture‐sensitive compounds were carried out under an argon atmosphere and in anhydrous solvents. NMR spectra were recorded on Bruker AM 250 XP, AV 300, AV 400, AV 500, or DRX 600 spectrometers (Bruker Corporation, Billerica, MA, USA). Chemical shifts (*δ*) are reported in ppm relative to TMS, coupling constants (*J*) in Hz. Multiplicity of signals is indicated as s for singlet, d for doublet, t for triplet, q for quartet, and m for multiplet. Aromatic signals resembling a triplet that stem from protons with two unequal neighbors but similar or equal coupling constants are denoted as a doublet of doublets (dd). Mass spectra were obtained on a VG Platform II (Thermo Fischer Scientific, Inc., Waltham, MA, USA) using electrospray ionization (ESI). Accurate mass spectra were recorded with ESI on a Bruker MicrOTOF‐Q II mass spectrometer (Bremen, Germany). Compound purity was determined using a Hitachi Chromaster HPLC system (Hitachi Europe, Krefeld, Germany). Separation was performed on an Avantor ACE 5 C18 column (250 × 4.0 mm, 5 µm; VWR International GmbH, Darmstadt, Germany) at a flow rate of 1 mL/min. The mobile phase consisted of water (+0.1% formic acid) and acetonitrile. The elution profile started with an isocratic step (80:20) for 5 min, followed by a linear gradient to 5:95 over 20 min, which was then held for an additional 5 min. Detection was carried out via UV at 254 and 280 nm. All final compounds for biological evaluation had a purity >95% according to the AUC at 254 and 280 nm UV detection. All compounds with biological activity were examined for pan‐assay interference compounds (PAINS) using SwissADME [29], and no alerts were returned.

#### 4.1.2 General Procedures

A. *Amide* *s* *ynthesis I*. A mixture of the respective carboxylic acid (1.3 equiv) and *O*‐(7‐azabenzotriazol‐1‐yl)‐*N* *,* *N* *,* *N′* *,* *N′*‐tetramethyluronium hexafluorophosphate (HATU, 1.3 equiv) was dissolved in dry tetrahydrofuran (THF) under an argon atmosphere. Then, a solution of the corresponding amine (1.0–2.0 equiv) dissolved in dry THF and *N*,*N*‐diisopropylethylamine (DIPEA, 1.5 equiv) was added slowly, and the mixture was stirred at room temperature for 15 h. The mixture was alkalized by addition of ammonia solution, and the organic components were extracted three times with ethyl acetate (EtOAc, 10 mL). The combined organic layers were dried over Na_2_SO_4_, and the solvent was evaporated under reduced pressure. The product was isolated by column chromatography on silica.
B. *Borylation*. Octamethyl‐2,2′‐bi‐1,3,2‐dioxaborolan (1.1 equiv), PdCl_2_(dppf) (0.04 equiv), and potassium acetate (3.0 equiv) were dissolved in dry dimethyl sulfoxide (DMSO) under an argon atmosphere. The bromoarene (1.0 equiv) dissolved in dry DMSO was added, and the mixture was stirred at 80 °C for 6 h. After cooling to room temperature, water was added, and the organic components were extracted three times with EtOAc (10 mL). Purification by column chromatography on silica afforded a pure product.
C. *Suzuki* *r* *eaction*. The bromoarene (1.0–1.2 equiv) and Na_2_CO_3_ (3.0 equiv) were dissolved in a 4:1 mixture of 1,4‐dioxane and water under an argon atmosphere and degassed. A catalytic amount of Pd(PPh_3_)_4_ (0.05 equiv) and the corresponding boronic acid (1.0–1.2 equiv) were added. The mixture was heated to reflux for 2 h. After cooling to room temperature, the mixture was acidified by the addition of 5% aqueous HCl solution, and the organic components were extracted three times with EtOAc (10 mL each). The combined organic layers were dried over Na_2_SO_4_, and the solvent was evaporated under reduced pressure. The product was purified by column chromatography on silica.
D. *Deprotection of BOC* *g* *roups*. The BOC‐protected amine (1.0 equiv) was dissolved in CH_2_Cl_2_/trifluoroacetic acid (TFA) 3:1, and the reaction mixture was stirred at room temperature for 2 h. The solvent was evaporated and gave the crude product, which was used for the next step without further purification.
E. *Amide synthesis II*. The secondary amine (1.0 equiv), (benzotriazol‐1‐yloxy)tripyrrolidinophosphonium hexafluorophosphate (PyBOP, 1.2 equiv), and DIPEA (8.0 equiv) were dissolved in dry dimethylformamide (DMF). 3‐Methyl‐2‐oxobutanoic acid (2.0 equiv) was added, and the reaction mixture was stirred at room temperature for 18 h. The reaction was quenched with water, and the organic components were extracted three times with EtOAc (10 mL). The combined organic layers were dried over Na_2_SO_4_, and the solvents were evaporated under reduced pressure. The product was purified by column chromatography on silica.
F. *Nucleophilic trifluoromethylation*. A mixture of the respective carbonylic compound (1.0 equiv) dissolved in dry THF, trimethyl(trifluoromethyl)silane (CF_3_TMS, 2.0 equiv), and tetra‐*n*‐butylammonium fluoride (TBAF, 1.0 equiv) was stirred for 1 h at room temperature under an argon atmosphere. Then, additional TBAF (1.0 equiv) was added. The reaction mixture was stirred at room temperature for another 3.5 h. THF was removed under reduced pressure at 40 °C. The product was purified by column chromatography on silica.

#### 4.1.3 4‐Bromo‐2‐chloro‐*N*,*N*‐dimethylbenzamide (11)

Preparation according to general procedure (A) using 4‐bromo‐2‐chlorobenzoic acid (**9**, 1.50 g, 6.35 mmol, 1.3 equiv), 2 mol/L dimethylamine in THF (**10**, 2.44 mL, 4.88 mmol, 1.0 equiv), HATU (2.42 g, 6.35 mmol, 1.3 equiv), and DIPEA (1.26 mL, 7.33 mmol, 1.5 equiv) in THF (4 mL). Further purification was performed by column chromatography on silica with 0%–2% MeOH in CH_2_Cl_2_ to obtain **11** as a pale‐yellow oil (544 mg, 42%). *R*_f_ = 0.71 (CH_2_Cl_2_/MeOH 99:1); ^1^H NMR (300 MHz, CDCl_3_): *δ* = 7.57 (d, *J = *1.8 Hz, 1H), 7.45 (dd, *J = *8.2, 1.8 Hz, 1H), 7.17 (d, *J = *8.2 Hz, 1H), 3.12 (s, 3H), 2.85 (s, 3H); ^13^C{^1^H} NMR (75 MHz, CDCl_3_): *δ* = 167.5, 135.3, 132.3, 131.4, 130.5, 129.0, 123.1, 38.0, 34.7; MS (ESI+): *m/z* 283.97 [M+Na]^+^.

#### 4.1.4 *tert*‐Butyl 4‐(3′‐Chloro‐4′‐[dimethylcarbamoyl[‐1,1′‐biphenyl]‐4‐yl)piperazine‐1‐carboxylate (14a)

Preparation according to general procedure (C) using *tert*‐butyl 4‐(4‐[4,4,5,5‐tetramethyl‐1,3,2‐dioxaborolan‐2‐yl]phenyl)piperazine‐1‐carboxylate (**17**, 702 mg, 2.29 mmol, 1.0 equiv), Na_2_CO_3_ (729 mg, 6.88 mmol, 3.0 equiv), Pd(PPh_3_)_4_ (132 mg, 115 µmol, 0.05 equiv), and **11** (722 mg, 2.75 mmol, 1.2 equiv) in 1,4‐dioxane/water (4:1, 5 mL). Further purification was performed by reversed‐phase column chromatography on silica with acetonitrile in water (5%–95%) to obtain **14a** as a colorless solid (851 mg, 84%). *R*_f_ = 0.34 (CH_2_Cl_2_/MeOH 15:1); ^1^H NMR (300 MHz, CDCl_3_): *δ *= 7.50 (d, *J = *1.6 Hz, 1H), 7.46–7.38 (m, 3H), 7.25 (d, *J = *8.0 Hz, 1H), 6.95–6.87 (m, 2H), 3.53 (t, *J = *5.2 Hz, 4H), 3.14 (t, *J = *5.2 Hz, 4H), 3.08 (s, 3H), 2.84 (s, 3H), 1.44 (s, 9H); ^13^C{^1^H} NMR (75 MHz, CDCl_3_): *δ* = 168.4, 154.6, 151.0, 142.8, 133.9, 130.5, 129.9, 128.0, 127.6, 127.0, 125.0, 116.3, 79.8, 48.6, 43.3, 38.0, 34.6, 28.3; MS (ESI+): *m/z* 444.00 [M+H]^+^.

#### 4.1.5 3‐Chloro‐*N*,*N*‐dimethyl‐4′‐(4‐[3‐methyl‐2‐oxobutanoyl]piperazin‐1‐yl)‐[1,1′‐biphenyl]‐4‐carboxamide (16a)

Preparation according to general procedures (D) and (E) using **14a** (31 mg, 0.070 mol, 1.0 equiv), PyBOP (44 mg, 84 µmol, 1.2 equiv), DIPEA (97 µL, 0.56 mmol, 8.0 equiv), and 3‐methyl‐2‐oxobutanoic acid (**15**, 16 mg, 0.14 mmol, 2.0 equiv) in DMF (4 mL). Further purification was performed by column chromatography on silica with 0.8%–6.6% MeOH in CH_2_Cl_2_ to obtain **16a** as a colorless oil (24 mg, 78%). *R*_f_ = 0.37 (CH_2_Cl_2_/MeOH 99:1); ^1^H NMR (300 MHz, acetone‐*d*_6_): *δ* = 7.68 (d, *J =* 1.9 Hz, 2H), 7.69–7.58 (m, 4H), 7.35 (d, *J = *8.0 Hz, 1H), 7.17–7.05 (m, 2H), 3.78 (t, *J* = 5.2 Hz, 2H), 3.58 (t, *J* = 5.2 Hz, 2H), 3.41–3.28 (m, 4H), 3.14 (septet, *J* = 7.0 Hz, 1H), 3.07 (s, 3H), 2.89 (s, 3H), 1.16 (d, *J = *7.0 Hz, 6H); ^13^C{^1^H} NMR (75 MHz, acetone‐*d*_6_): *δ* = 204.3, 167.3, 165.8, 151.1, 142.6, 134.9, 130.3, 129.7, 128.4, 127.6, 126.6, 124.9, 116.5, 49.0, 48.4, 45.2, 40.8, 37.4, 37.2, 33.5, 16.2; MS (ESI+): *m/z* 441.97 [M+H]^+^.

#### 4.1.6 3‐Chloro‐4′‐(4‐[2‐hydroxy‐3‐methyl‐2‐{trifluoromethyl}butanoyl]piperazin‐1‐yl)‐*N*,*N*‐dimethyl‐[1,1′‐biphenyl]‐4‐carboxamide (*rac*‐5)

Preparation according to general procedure (F) using **16a** (48 mg, 0.11 mmol, 1.0 equiv), 2 mol/L CF_3_TMS in THF (0.11 mL, 0.22 mmol, 2.0 equiv), and 1 mol/L TBAF in THF (0.22 mL, 0.22 mmol, 2.0 equiv) in THF (3 mL). Further purification was performed by column chromatography on silica with 0.8%–10% MeOH in CH_2_Cl_2_ to obtain (*rac*)‐**5** as a colorless oil (28 mg, 50%). *R*_f_ = 0.37 (CH_2_Cl_2_/MeOH 15:1); ^1^H NMR (300 MHz, acetone‐*d*_6_): *δ* = 7.68 (d, *J =* 1.7 Hz, 1H), 7.67–7.59 (m, 3H), 7.35 (d, *J = *7.9 Hz, 1H), 7.14–7.06 (m, 2H), 5.71 (s, 1H), 4.24–3.77 (m, 4H), 3.35 (t, *J = *5.2 Hz, 4H), 3.07 (s, 3H), 2.89 (s, 3H), 2.68 (septet, *J = *6.7 Hz, 1H), 1.09 (d, *J = *6.7 Hz, 3H), 0.93 (d, *J = *6.8 Hz, 3H); ^19^F NMR (282 MHz, acetone‐*d*_6_): *δ* = −71.39, −71.58, −73.90; ^13^C{^1^H} NMR (75 MHz, acetone‐*d*_6_): *δ* = 168.2, 167.8, 151.9, 143.6, 135.7, 131.2, 130.5, 129.3, 128.5, 127.4, 125.8, 125.7 (q, *J = *148.0 Hz), 117.0, 49.3, 46.6–45.9 (m, 2C), 38.1, 34.4, 32.6, 17.6, 16.9; Accurate mass (ESI): *m/z* calculated for C_25_H_29_ClF_3_N_3_O_3_ [M+H]^+^: 512.19223; found: 512.1938. Asymmetric carbon atom is not visible in ^13^C NMR.

#### 4.1.7 2‐Chloro‐*N*,*N*‐dimethyl‐4‐(4,4,5,5‐tetramethyl‐1,3,2‐dioxaborolan‐2‐yl)benzamide (12)

Preparation according to general procedure (B) using **11** (502 mg, 1.91 mmol, 1.0 equiv), octamethyl‐2,2′‐bi‐1,3,2‐dioxaborolan (535 mg, 2.10 mmol, 1.1 equiv), PdCl_2_(dppf) (64 mg, 76 µmol, 0.04 equiv), and potassium acetate (563 mg, 5.74 mmol, 3.0 equiv) in DMSO (7 mL). Further purification was performed by column chromatography on silica with *n‐*hexane/acetone (8:1–1:1) to obtain **12** as a colorless solid (75 mg, 13%). *R*_f_ = 0.74 (CH_2_Cl_2_/MeOH 99:1); ^1^H NMR (400 MHz, CDCl_3_): *δ* = 7.81 (d, *J* = 0.8 Hz, 1H), 7.71 (dd, *J = *7.5, 0.8 Hz, 1H), 7.28 (d, *J = *7.5 Hz, 1H), 3.13 (s, 3H), 2.83 (s, 3H), 1.23 (s, 12H); ^11^B{^1^H} NMR (128 MHz, CDCl_3_): *δ* = 30.18; ^13^C{^1^H} NMR (101 MHz, CDCl_3_): *δ* = 168.4, 138.7, 135.6, 133.9, 133.3, 130.0, 127.2, 84.4, 75.0, 38.0, 34.6, 24.9; MS (ESI+): *m/z* 332.05 [M+Na]^+^.

#### 4.1.8 *tert*‐Butyl (3a*R*,6a*S*)‐5‐(5‐Bromopyridin‐2‐yl)hexahydropyrrolo[3,4‐*c*]pyrrole‐2(1*H*)‐carboxylate (13b)

*Tert*‐butyl (3a*R*,6a*S*)‐hexahydropyrrolo[3,4‐*c*]pyrrole‐2(1*H*)‐carboxylate (**19**, 663 mg, 3.12 mmol, 1.1 equiv), 5‐bromo‐2‐fluoropyridine (**18**, 500 mg, 2.84 mmol, 1.0 equiv), and K_2_CO_3_ (789 mg, 5.68 mmol, 2.0 equiv) were dissolved in DMSO (30 mL) under an argon atmosphere. The mixture was heated to 80 °C and stirred for 5 h. After cooling to room temperature, EtOAc was added, and the organic layer was washed three times with water (10 mL). It was dried over Na_2_SO_4,_ and the solvent was evaporated. The product was isolated by column chromatography on silica with *n‐*hexane/EtOAc (40:1–3:1) to obtain **13b** as a colorless oil (741 mg, 71%). *R*_f_ = 0.31 (cyclohexane/EtOH 10:1); ^1^H NMR (300 MHz, CDCl_3_): *δ* = 8.17 (s, 1H), 7.52 (d, *J = *9.0 Hz, 1H), 6.27 (dd, *J = *9.0, 3.9 Hz, 1H), 3.80–3.51 (m, 4H), 3.52–3.14 (m, 4H), 3.10–2.87 (m, 2H), 1.44 (s, 9H); ^13^C{^1^H} NMR (75 MHz, CDCl_3_): δ = 154.6, 147.9, 140.1, 108.4, 106.4, 79.7, 51.3, 50.3, 49.9, 42.3, 41.4, 28.6; MS (ESI+): *m/z* 389.93 [M+Na]^+^.

#### 4.1.9 *tert*‐Butyl (3a*R*,6a*S*)‐5‐(5‐[3‐Chloro‐4‐{dimethylcarbamoyl}phenyl]pyridin‐2‐yl)hexahydropyrrolo[3,4‐*c*]pyrrole‐2(1*H*)‐carboxylate (14b)

Preparation according to general procedure (C) using **12** (50 mg, 0.16 mmol, 1.2 equiv), Na_2_CO_3_ (43 mg, 0.40 mmol, 3.0 equiv), Pd(PPh_3_)_4_ (8 mg, 7 µmol, 0.05 equiv), and **13b** (50 mg, 0.14 mmol, 1.0 equiv) in 1,4‐dioxane/water (4:1, 5 mL). Further purification was performed by column chromatography on silica with *n‐*hexane/acetone (24:1–3:1) to obtain **14b** as a colorless oil (51 mg, 80%). *R*_f_ = 0.29 (cyclohexane/acetone 4:1); ^1^H NMR (400 MHz, CDCl_3_): *δ* = 8.37 (dd, *J = *2.5, 0.7 Hz, 1H), 7.65 (dd, *J = *8.8, 2.5 Hz, 1H), 7.50 (d, *J = *1.7 Hz, 1H), 7.42 (dd, *J = *7.9, 1.7 Hz, 1H), 7.31 (d, *J = *7.9 Hz, 1H), 6.42 (d, *J = *8.8 Hz, 1H), 3.81–3.70 (m, 2H), 3.71–3.59 (m, 2H), 3.53–3.21 (m, 4H), 3.14 (s, 3H), 3.07–2.97 (m, 2H), 2.91 (s, 3H), 1.44 (s, 9H); ^13^C{^1^H} NMR (101 MHz, CDCl_3_): *δ* = 168.5, 156.7, 154.6, 146.4, 140.9, 135.8, 134.2, 131.0, 128.4, 126.8, 124.8, 122.9, 106.8, 79.6, 51.1, 50.3, 50.0, 42.2, 41.4, 38.3, 34.8, 28.6; MS (ESI+): *m/z* 470.97 [M+H]^+^.

#### 4.1.10 2‐Chloro‐*N*,*N*‐dimethyl‐4‐(6‐[{3a*R*,6a*S*}‐5‐{3‐methyl‐2‐oxobutanoyl}hexahydropyrrolo[3,4‐*c*]pyrrol‐2{1*H*}‐yl]pyridin‐3‐yl)benzamide (16b)

Preparation according to general procedures (D) and (E) using **14b** (77 mg, 0.16 mmol, 1.0 equiv), PyBOP (102 mg, 197 µmol, 1.2 equiv), DIPEA (230 µL, 1.31 mmol, 8.0 equiv), and 3‐methyl‐2‐oxobutanoic acid (**15**, 38 mg, 0.33 mmol, 2.0 equiv) in DMF (6 mL). Further purification was performed by reversed‐phase column chromatography on silica with 5%–95% acetonitrile in water to obtain **16b** as a colorless oil (45 mg, 59%). *R*_f_ = 0.25 (CH_2_Cl_2_/MeOH 99:1); ^1^H NMR (300 MHz, acetone‐*d*_6_): *δ* = 8.46 (dd, *J = *2.6, 0.7 Hz, 1H), 7.82 (dd, *J = *8.8, 2.6 Hz, 1H), 7.66 (d, *J = *1.7 Hz, 1H), 7.59 (dd, *J = *8.0, 1.8 Hz, 1H), 7.35 (d, *J = *7.9 Hz, 1H), 6.51 (d, *J = *8.8 Hz, 1H), 3.92–3.67 (m, 4H), 3.57–3.37 (m, 4H), 3.31–3.05 (m, 6H), 2.88 (s, 3H), 1.10 (d, *J = *4.4 Hz, 3H), 1.08 (d, *J = *4.4 Hz, 3H); ^13^C{^1^H} NMR (75 MHz, acetone‐*d*_6_): *δ* = 204.9, 168.2, 165.1, 158.1, 147.4, 141.7, 136.2, 135.6, 131.3, 129.3, 126.9, 125.3, 123.1, 107.4, 51.8, 51.6, 51.3, 50.8, 43.5, 41.0, 38.1, 37.3, 34.4, 17.3, 17.2; MS (ESI+): *m/z* 469.02 [M+H]^+^.

#### 4.1.11 2‐Chloro‐4‐(6‐[{3a*R*,6a*S*}‐5‐{2‐hydroxy‐3‐methyl‐2‐(trifluoromethyl)butanoyl}hexahydropyrrolo[3,4‐*c*]pyrrol‐2{1*H*}‐yl]pyridin‐3‐yl)‐*N*,*N*‐dimethylbenzamide (*rac*‐6)

Preparation according to general procedure (F) using **16b** (165 mg, 351 µmol, 1.0 equiv), 2 mol/L CF_3_TMS in THF (0.35 mL, 0.70 mmol, 2.0 equiv), and 1 mol/L TBAF in THF (0.70 mL, 0.70 mmol, 2.0 equiv) in THF (9 mL). Further purification was performed by reversed‐phase column chromatography on silica with 10%–90% acetonitrile in water to obtain (*rac*)**‐6** as a colorless oil (132 mg, 70%). *R*_f_ = 0.27 (CH_2_Cl_2_/MeOH 99:1); ^1^H NMR (300 MHz, acetone‐*d*_6_): *δ* = 8.47 (d, *J = *2.6 Hz, 1H), 7.85 (dd, *J = *8.8, 2.6 Hz, 1H), 7.67 (d, *J = *1.7 Hz, 1H), 7.61 (dd, *J = *8.0, 1.7 Hz, 1H), 7.36 (d, *J = *7.9 Hz, 1H), 6.55 (d, *J = *8.8 Hz, 1H), 5.52 (s, 1H), 4.20–4.00 (m, 1H), 4.00–3.69 (m, 4H), 3.68–3.52 (s, 1H), 3.52–3.38 (m, 2H), 3.30–3.01 (m, 5H), 2.89 (s, 3H), 2.66 (septet, *J = *6.8 Hz, 1H), 1.05 (d, *J = *6.8 Hz, 3H), 0.86 (d, *J = *6.8 Hz, 3H); ^19^F NMR (282 MHz, acetone‐*d*_6_): *δ* = −71.90; ^13^C{^1^H} NMR (75 MHz, acetone‐*d*_6_): *δ* = 167.3, 166.7 (q, *J = *1.9 Hz), 157.2, 146.5, 140.8, 135.3, 134.7, 130.4, 128.5, 126.1, 125.1 (q, *J = *288.0 Hz), 124.5, 122.3, 106.5, 81.3 (q, *J = *28.0 Hz), 53.3, 51.2, 50.9, 50.3, 43.3, 39.0, 37.2, 33.6, 30.5, 16.5, 15.6; Accurate mass (ESI): *m/z* calculated for C_26_H_30_ClF_3_N_4_O_3_ [M+H]^+^: 539.20313; found: 539.2033.

#### 4.1.12 ([5‐Bromo‐1*H*‐inden‐3‐yl]oxy)(*tert*‐butyl)dimethylsilane (21)

6‐Bromo‐2,3‐dihydro‐1*H*‐inden‐1‐one (**20**, 382 mg, 1.76 mmol, 1.0 equiv) was dissolved in dry toluene (5 mL) under an argon atmosphere and cooled to 0 °C. *tert*‐Butylchlorodimethylsilane (TBDMSCl, 432 mg, 2.81 mmol, 1.6 equiv) dissolved in toluene (2 mL) was added. 1,8‐Diazabicycloundec‐7‐ene (DBU, 480 µL, 3.16 mmol, 1.8 equiv) was added drop by drop, and the reaction mixture was stirred at 0 °C for ten minutes. Then, the ice bath was removed, and the reaction mixture was stirred at room temperature for 21 h. The reaction mixture was suspended in *n*‐hexane and filtrated twice over celite. Purification by column chromatography on silica with 0%–5% EtOAc in *n*‐hexane afforded **21** as a colorless oil (235 mg, 41%). *R*_f_ = 0.95 (*n*‐hexane/EtOAc 10:1); ^1^H NMR (300 MHz, CDCl_3_): δ = 7.47 (d, *J = *1.8 Hz, 1H), 7.33 (dd, *J = *7.9, 1.8 Hz, 1H), 7.24 (d, *J* = 4.9 Hz, 1H), 5.43 (t, *J = *2.5 Hz, 1H), 3.22 (d, *J = *2.4 Hz, 2H), 1.02 (s, 9H), 0.25 (s, 6H); ^29^Si{^1^H} NMR (99 MHz, CDCl_3_): δ = 22.81; ^13^C{^1^H} NMR (75 MHz, CDCl_3_): δ = 152.9, 144.1, 141.4, 127.9, 125.1, 121.4, 120.2, 107.1, 33.7, 25.7, 18.3, −4.7; MS (ESI+): *m/z* 324.94 [M+H]^+^.

#### 4.1.13 *tert*‐Butyl 5‐Bromo‐3‐oxo‐2,3‐dihydrospiro[indene‐1,4′‐piperidine]‐1′‐carboxylate (23)

Compound **21** (1.30 g, 4.00 mmol, 1.0 equiv) dissolved in dry THF (7 mL) under an argon atmosphere was cooled to −78 °C. A solution of 1.3 M lithium bis(trimethylsilyl)amide (LiHMDS) in THF (12.3 mL, 16.0 mmol, 4.0 equiv) was added drop by drop, and the reaction mixture was stirred for 30 min. *tert*‐Butylbis(2‐chloroethyl)carbamate (**22**, 1.20 g, 4.80 mmol, 1.2 equiv) dissolved in dry THF (5 mL) was added, and the reaction mixture was slowly allowed to warm to room temperature and was then stirred for 22 h. Water was added, and the organic components were extracted three times with EtOAc (10 mL each). The organic layers were dried over MgSO_4_, and the solvents were evaporated. The crude product was dissolved in dry THF (5 mL) under an argon atmosphere, and a solution of 1 mol/L TBAF in THF (8.0 mL, 8.0 mmol, 2.0 equiv) was added. The reaction mixture was stirred at room temperature for 4 h. The mixture was acidified by the addition of 10% aqueous HCl to pH 4. The organic components were extracted three times with EtOAc (10 mL each). The product was purified by column chromatography on silica with *n‐*hexane/EtOAc (20:1–1:1) to obtain **23** as a pale‐yellow solid (975 mg, 64%). *R*_f_ = 0.81 (*n*‐hexane/EtOAc 2:1); ^1^H NMR (300 MHz, CDCl_3_): *δ* = 7.85 (d, *J = *2.0 Hz, 1H), 7.73 (dd, *J = *8.1, 2.0 Hz, 1H), 7.37 (d, *J = *8.1 Hz, 1H), 4.23 (dt, *J = *13.4, 2.1 Hz, 2H), 2.84 (td, *J* = 13.0, 2.4 Hz, 2H), 2.64 (s, 2H), 1.94 (td, *J = *13.2, 4.6 Hz, 2H), 1.59–1.41 (m, 11H); ^13^C{^1^H} NMR (75 MHz, CDCl_3_): *δ* = 203.0, 160.6, 154.8, 138.0, 137.6, 126.7, 125.6, 122.4, 80.0, 47.0, 41.6, 41.5, 37.5, 28.5; MS (ESI+): *m/z* not found.

#### 4.1.14 *tert*‐Butyl 5‐Bromo‐2,3‐dihydrospiro[indene‐1,4′‐piperidine]‐1′‐carboxylate (13c)

Compound **23** (60 mg, 0.16 mmol, 1.0 equiv), potassium hydroxide (52 mg, 0.79 mmol, 5.0 equiv), and hydrazine hydrate (20 µL, 0.47 mmol, 1.4 equiv) were dissolved in diethylene glycol (3 mL) under an argon atmosphere and heated to 200 °C for 2.5 h. After cooling to room temperature, methanol/NEt_3_ 4:1 (2.0/0.5 mL) and di‐*tert*‐butyl dicarbonate (138 mg, 631 µmol, 4.0 equiv) were added, and the reaction mixture was stirred for 21 h. Water was added, and the organic components were extracted three times with EtOAc (10 mL each). Column chromatography on silica with *n‐*hexane/EtOAc (10:1–1:1) afforded **13c** as a colorless oil (29 mg, 50%). *R*_f_ = 0.90 (*n*‐hexane/EtOAc 2:1); ^1^H NMR (300 MHz, CDCl_3_): *δ* = 7.38–7.24 (m, 2H), 6.99 (d, *J = *8.0 Hz, 1H), 4.09 (d, *J = *13.6 Hz, 2H), 3.02–2.82 (m, 4H), 2.05 (t, *J = *7.4 Hz, 2H), 1.73 (td, *J = *13.1, 4.6 Hz, 2H), 1.53–1.44 (m, 11H); ^13^C{^1^H} NMR (75 MHz, CDCl_3_): *δ* = 155.0, 149.8, 145.4, 129.6, 127.9, 124.0, 120.5, 79.5, 46.3, 41.3, 36.3, 34.2, 29.7, 28.5; MS (ESI+): *m/z* 367.13 [M+H]^+^.

#### 4.1.15 *tert*‐Butyl 5‐(3‐Chloro‐4‐[dimethylcarbamoyl]phenyl)‐2,3‐dihydrospiro[indene‐1,4′‐piperidine]‐1′‐carboxylate (14c)

Preparation according to general procedure (C) using **13c** (125 mg, 342 µmol, 1.0 equiv), Na_2_CO_3_ (109 mg, 1.03 mmol, 3.0 equiv), Pd(PPh_3_)_4_ (20 mg, 17 µmol, 0.05 equiv), and **12** (127 mg, 410 µmol, 1.2 equiv) in 1,4‐dioxane/water (4:1, 5 mL). Further purification was performed by column chromatography on silica with *n‐*hexane/EtOAc (10:1–1:1) to obtain **14c** as a white solid (135 mg, 84%). *R*_f_ = 0.58 (CH_2_Cl_2_/MeOH 99:1); ^1^H NMR (500 MHz, CD_2_Cl_2_): *δ* = 7.61 (d, *J = *1.7 Hz, 1H), 7.52 (dd, *J = *7.9, 1.7 Hz, 1H), 7.47–7.38 (m, 2H), 7.32 (d, *J = *7.9 Hz, 1H), 7.23 (d, *J = *7.8 Hz, 1H), 4.15–4.01 (m, 2H), 3.10 (s, 3H), 3.03–2.90 (m, 4H), 2.88 (s, 3H), 2.12 (t, *J = *7.4 Hz, 2H), 1.80 (td, *J =* 13.0, 4.7 Hz, 2H), 1.58–1.53 (m, 2H), 1.47 (s, 9H); ^13^C{^1^H} NMR (126 MHz, CD_2_Cl_2_): *δ* = 167.9, 154.7, 151.3, 144.2, 143.5, 137.9, 135.0, 130.5, 128.0, 127.9, 125.8, 125.5, 123.3, 122.9, 79.0, 46.5, 37.8, 36.3, 34.30, 34.27, 29.8, 28.2; MS (ESI+): *m/z* 491.10 [M+Na]^+^.

#### 4.1.16 2‐Chloro‐*N*,*N*‐dimethyl‐4‐(1′‐[3‐methyl‐2‐oxobutanoyl]‐2,3‐dihydrospiro[indene‐1,4′‐piperidin]‐5‐yl)benzamide (16c)

Preparation according to general procedures (D) and (E) using **14c** (81 mg, 0.22 mmol, 1.0 equiv), PyBOP (140 mg, 263 µmol, 1.2 equiv), DIPEA (300 µL, 1.76 mmol, 8.0 equiv), and 3‐methyl‐2‐oxobutanoic acid (**15**, 52 mg, 0.44 mmol, 2.0 equiv) in DMF (2 mL). Further purification was performed by column chromatography on silica with 11%–100% EtOAc in *n‐*hexane to obtain **16c** as a colorless solid (66 mg, 64%). *R*_f_ = 0.95 (CH_2_Cl_2_/MeOH 99:1); ^1^H NMR (300 MHz, CD_2_Cl_2_): *δ* = 7.61 (d, *J = *1.6 Hz, 1H), 7.52 (dd, *J = *7.9, 1.7 Hz, 1H), 7.47–7.37 (m, 2H), 7.32 (d, *J = *7.9 Hz, 1H), 7.24 (d, *J = *7.9 Hz, 1H), 4.56–4.43 (m, 1H), 3.67–3.55 (m, 1H), 3.28 (td, *J = *13.2, 2.9 Hz, 1H), 3.22–3.08 (m, 4H), 3.01 (t, *J = *7.2 Hz, 2H), 2.97–2.86 (m, 4H), 2.17 (td, *J = *7.7, 3.3 Hz, 2H), 1.95–1.78 (m, 2H), 1.64 (t, *J = *13.7 Hz, 2H), 1.18 (dd, *J = *6.9, 6.9 Hz, 6H); ^13^C{^1^H} NMR (75 MHz, CD_2_Cl_2_): *δ* = 205.0, 167.9, 166.2, 150.3, 144.1, 143.4, 138.2, 135.1, 130.5, 128.1, 127.9, 125.8, 125.7, 123.4, 122.9, 46.8, 43.5, 38.9, 37.9, 37.7, 36.8, 35.9, 34.5, 34.3, 29.8, 16.6, 16.5; MS (ESI+): *m/z* 489.10 [M+Na]^+^.

#### 4.1.17 2‐Chloro‐4‐(1′‐[2‐hydroxy‐3‐methyl‐2‐{trifluoromethyl}butanoyl]‐2,3‐dihydrospiro[indene‐1,4′‐piperidin]‐5‐yl)‐*N*,*N*‐dimethylbenzamide (*rac‐*7)

Preparation according to general procedure (F) using **16c** (200 mg, 428 µmol, 1.0 equiv), 2 mol/L CF_3_TMS in THF (0.43 mL, 0.86 mmol, 2.0 equiv), and 1 mol/L TBAF in THF (0.86 mL, 0.86 mmol, 2.0 equiv) in THF (5 mL). Further purification was performed by column chromatography on silica with *n‐*hexane/acetone (4:1) to obtain (*rac*)‐**7** as a white solid (179 mg, 78%). *R*_f_ = 0.78 (*n*‐hexane/acetone 2:1); ^1^H NMR (500 MHz, CD_2_Cl_2_): *δ* = 7.61 (d, *J = *1.7 Hz, 1H), 7.52 (dd, *J = *7.9, 1.7 Hz, 1H), 7.46 (d, *J* = 1.6 Hz, 1H), 7.42 (dd, *J* = 7.8, 2.0 Hz, 1H), 7.33 (d, *J = *7.9 Hz, 1H), 7.20 (d, *J = *7.9 Hz, 1H), 5.71 (s, 1H), 4.79–4.14 (m, 2H), 3.51–3.07 (m, 5H), 3.02 (t, *J = *7.3 Hz, 2H), 2.88 (s, 3H), 2.51 (septet, *J = *6.3 Hz, 1H), 2.18 (t, *J = *7.3 Hz, 2H), 1.92–1.80 (m, 2H), 1.73–1.64 (m, 2H), 1.11 (d, *J* = 6.4 Hz, 3H), 1.03–0.79 (m, 3H); ^19^F NMR (471 MHz, CD_2_Cl_2_): *δ* = −70.05, −71.19; ^13^C{^1^H} NMR (126 MHz, CD_2_Cl_2_): *δ* = 167.9, 167.0, 150.0, 144.1, 143.3, 138.2, 135.1, 130.5, 128.1, 127.9, 125.8, 125.7, 123.52 (q, *J* = 286.0 Hz), 123.45, 122.8, 81.1, 46.5, 43.7, 37.9, 36.4, 34.8, 34.3, 31.0, 29.8, 16.9, 16.4; Accurate mass (ESI): *m/z* calculated for C_28_H_32_ClF_3_N_2_O_3_ [M+H]^+^: 537.21263; found: 537.2118.

#### 4.1.18 *tert*‐Butyl 4‐Hydroxy‐4‐([phenylsulfinyl]methyl)piperidine‐1‐carboxylate (26)

(Methylsulfinyl)benzene (**25**, 1.23 g, 8.68 mmol, 1.3 equiv) was dissolved in dry THF (5 mL) under an argon atmosphere and cooled to −78 °C. 2 mol/L Lithium diisopropylamide (LDA) in THF/heptane/ethylbenzene (3.44 mL, 6.89 mmol, 1.0 equiv) was added. After 30 min, *tert*‐butyl 4‐oxopiperidine‐1‐carboxylate (**24**, 1.40 g, 6.89 mmol, 1.0 equiv) was added, and the reaction mixture was allowed to warm to room temperature over 20 h. Saturated NH_4_Cl solution was added, and the organic components were extracted three times with EtOAc (10 mL). The product was purified by column chromatography on silica with 0%–4% MeOH in CH_2_Cl_2_ to obtain **26** as a pale‐yellow solid (906 mg, 39%). *R*_f_ = 0.54 (*n*‐hexane/EtOH 6:1); ^1^H NMR (300 MHz, CDCl_3_): *δ* = 8.77–7.29 (m, 5H), 3.93 (dt, *J* = 13.5, 4.7 Hz, 1H), 3.82 (dt, *J* *= *13.3, 4.6 Hz, 1H), 3.37–3.16 (m, 2H), 3.05 (d, *J = *13.3 Hz, 1H), 2.69 (d, *J = *13.3 Hz, 1H), 2.23–2.10 (m, 1H), 1.82 (td, *J = *12.6, 4.7 Hz, 1H), 1.69 (dt, *J = *13.4, 3.0 Hz, 1H), 1.57–1.49 (m, 1H), 1.45 (s, 9H); ^13^C{^1^H} NMR (75 MHz, CDCl_3_): *δ* = 154.8, 143.5, 131.6, 129.6, 123.9, 79.6, 70.3, 66.8, 39.6, 39.0, 38.2, 36.2, 28.5; MS (ESI+): *m/z* 362.02 [M+Na]^+^.

#### 4.1.19 *tert*‐Butyl 3‐Hydroxy‐4‐methylenepiperidine‐1‐carboxylate (27)

Compound **26** (906 mg, 2.67 mmol, 1.0 equiv) and potassium *tert*‐butoxide (390 mg, 3.47 mmol, 1.3 equiv) were dissolved in *tert*‐butanol (5 mL) under an argon atmosphere and stirred for 3 h at 82 °C. After cooling to room temperature, saturated NH_4_Cl solution was added, and the organic components were extracted three times with EtOAc (10 mL). The combined organic layers were dried over Na_2_SO_4,_ and the solvents were evaporated. Purification was performed by column chromatography on silica with *n‐*hexane/acetone (10:1–3:1) to obtain **27** as a yellow resin (345 mg, 61%). *R*_f_ = 0.21 (*n*‐hexane/acetone 10:1); ^1^H NMR (400 MHz, CDCl_3_): *δ* = 5.05–4.98 (m, 1H), 4.87 (p, *J = *1.3 Hz, 1H), 4.15–4.07 (m, 1H), 3.76 (ddd, *J = *13.0, 4.2, 1.1 Hz, 1H), 3.64–3.49 (m, 1H), 3.34–3.11 (m, 2H), 2.43 (dt, *J* = 14.0, 5.0 Hz, 1H), 2.20–2.01 (m, 2H), 1.46 (s, 9H); ^13^C{^1^H} NMR (75 MHz, CDCl_3_): *δ* = 155.2, 147.1, 108.3, 80.0, 70.1, 51.5, 45.2, 32.2, 28.4; MS (ESI+): *m/z* 236.20 [M+Na]^+^.

#### 4.1.20 *tert*‐Butyl 4‐(Chloromethyl)‐3,6‐dihydropyridine‐1(2*H*)‐carboxylate (28)

Compound **27** (345 mg, 1.62 mmol, 1.0 equiv) dissolved in dry toluene (3 mL) under an argon atmosphere was cooled to 0 °C. 2,6‐Lutidine (230 µL, 1.94 mmol, 1.2 equiv) and SOCl_2_ (140 µL, 2.59 mmol, 1.6 equiv) were added. The reaction mixture was stirred at 40 °C for 1 h. The mixture was cooled to 0 °C and neutralized with a cold NaHCO_3_ solution. The product was extracted three times with EtOAc (10 mL). Further purification was performed by column chromatography on silica with *n‐*hexane/EtOAc (10:1–2:1) to obtain **28** as a brown oil (157 mg, 42%). *R*_f_ = 0.76 (*n*‐hexane/EtOAc 2:1); ^1^H NMR (300 MHz, CDCl_3_): *δ* = 5.80–5.70 (m, 1H), 4.06–3.96 (m, 2H), 3.96–3.86 (m, 2H), 3.52 (t, *J = *5.7 Hz, 2H), 2.294–2.14 (m, 2H), 1.47 (s, 9H); ^13^C{^1^H} NMR (75 MHz, CDCl_3_): *δ* = 154.8, 133.1, 123.7, 79.8, 48.8, 43.1, 40.1, 28.5, 26.1; MS (ESI+)*: m/z* 254.15 [M+Na]^+^.

#### 4.1.21 *tert*‐Butyl 4‐([5‐Bromo‐2‐iodophenoxy]methyl)‐3,6‐dihydropyridine‐1(2*H*)‐carboxylate (30)

Compound **28** (157 mg, 678 µmol, 1.0 equiv), K_2_CO_3_ (103 mg, 745 µmol, 1.1 equiv), and 5‐bromo‐2‐iodophenol (**29**, 414 mg, 1.36 mmol, 2.0 equiv) were suspended in DMF (4 mL) and stirred under an argon atmosphere for 19 h at 70 °C. After cooling to room temperature, water was added, and the organic components were extracted three times with EtOAc (10 mL each). The combined organic layers were dried over Na_2_SO_4,_ and the solvents were evaporated. Column chromatography on silica with *n‐*hexane/EtOAc (20:1–8:1) afforded **30** as a yellow oil (231 mg, 69%). *R*_f_ = 0.28 (*n*‐hexane/acetone 20:1); ^1^H NMR (400 MHz, CDCl_3_): *δ* = 7.61 (d, *J = *8.3 Hz, 1H), 6.99–6.81 (m, 2H), 5.96–5.77 (m, 1H), 4.46 (s, 2H), 4.05–3.91 (m, 2H), 3.57 (t, *J = *5.6 Hz, 2H), 2.33–2.20 (m, 2H), 1.48 (s, 9H); ^13^C{^1^H} NMR (101 MHz, CDCl_3_): *δ* = 157.8, 154.9, 140.2, 131.6, 125.8, 122.8, 115.8, 84.8, 79.7, 72.3, 60.3, 28.5, 25.8, 14.2; MS (ESI+): *m/z* 515.85 [M+Na]^+^.

#### 4.1.22 *tert*‐Butyl 6‐Bromo‐2*H*‐spiro[benzofuran‐3,4′‐piperidine]‐1′‐carboxylate (13d)

Compound **30** (231 mg, 467 µmol, 1.0 equiv) and 2,4,6‐tris(diphenylamino)‐5‐fluoroisophthalonitrile (**31**, 3 mg, 0.02 mmol, 0.05 equiv) were placed in a 10 mL microwave vial under an argon atmosphere and dissolved in THF/H_2_O 9:1 (1 mL). DIPEA (40 µL, 2.3 mmol, 5.0 equiv) was added. The reaction mixture was stirred at room temperature under irradiation at 405 nm and 6 W for 24 h. Purification was performed by reversed‐phase column chromatography with 5%–95% acetonitrile in water to obtain **13d** as a colorless oil (117 mg, 68%). *R*_f_ = 0.18 (*n*‐hexane/EtOAc 10:1); ^1^H NMR (300 MHz, CDCl_3_): *δ* = 7.01 (dd, *J = *8.1, 1.6 Hz, 1H), 6.99–6.89 (m, 2H), 4.41 (s, 2H), 4.06 (d, *J = *13.9 Hz, 2H), 2.89 (td, *J* = 13.2, 2.7 Hz, 2H), 1.86–1.66 (m, 4H), 1.48 (s, 9H); ^13^C{^1^H} NMR (75 MHz, CDCl_3_): *δ* = 160.4, 154.8, 133.8, 124.0, 123.8, 121.6, 113.6, 80.5, 79.9, 44.4, 41.1, 35.9, 28.5; MS (ESI+): *m/z* 389.96 [M+Na]^+^.

#### 4.1.23 *tert*‐Butyl 6‐(3‐Chloro‐4‐[dimethylcarbamoyl]phenyl)‐2*H*‐spiro[benzofuran‐3,4′‐piperidine]‐1′‐carboxylate (14d)

Preparation according to general procedure (C) using **12** (119 mg, 384 µmol, 1.2 equiv), Na_2_CO_3_ (102 mg, 961 µmol, 3.0 equiv), Pd(PPh_3_)_4_ (19 mg, 16 µmol, 0.05 equiv), and **13d** (118 mg, 320 µmol, 1.0 equiv) in 1,4‐dioxane/water (4:1, 5 mL). Further purification was performed by column chromatography on silica with *n‐*hexane/EtOAc (2:1–1:1) to obtain **14d** as an off‐white solid (128 mg, 85%). *R*_f_ = 0.27 (*n‐*hexane/EtOAc 2:1); ^1^H NMR (500 MHz, CD_2_Cl_2_): *δ* = 7.60 (d, *J = *1.7 Hz, 1H), 7.51 (dd, *J = *7.9, 1.8 Hz, 1H), 7.32 (d, *J = *7.9 Hz, 1H), 7.21 (d, *J = *7.7 Hz, 1H), 7.12 (dd, *J = *7.7, 1.6 Hz, 1H), 7.00 (d, *J = *1.6 Hz, 1H), 4.47 (s, 2H), 4.17–3.99 (m, 2H), 3.10 (s, 3H), 2.98–2.84 (m, 5H), 1.87 (td, *J = *13.2, 4.6 Hz, 2H), 1.78–1.70 (m, 2H), 1.47 (s, 9H); ^13^C{^1^H} NMR (126 MHz, CD_2_Cl_2_): *δ* = 167.9, 160.3, 154.6, 143.1, 139.9, 135.3, 135.0, 130.5, 128.1, 127.9, 125.9, 123.4, 119.7, 108.3, 80.4, 79.4, 44.6, 37.9, 35.9, 34.3, 28.1; MS (ESI+): *m/z* 493.07 [M+Na]^+^.

#### 4.1.24 2‐Chloro‐*N*,*N*‐dimethyl‐4‐(1′‐[3‐methyl‐2‐oxobutanoyl]‐2*H*‐spiro[benzofuran‐3,4′‐piperidin]‐6‐yl)benzamide (16d)

Preparation according to general procedures (D) and (E) using **14d** (102 mg, 274 µmol, 1.0 equiv), PyBOP (171 mg, 329 µmol, 1.2 equiv), DIPEA (370 µL, 2.19 mmol, 8.0 equiv), and 3‐methyl‐2‐oxobutanoic acid (**15**, 65 mg, 0.55 mmol, 2.0 equiv) in DMF (2 mL). Further purification was performed by column chromatography on silica with *n‐*hexane/acetone (6:1–3:1) to obtain **16d** as a colorless solid (65 mg, 51%). *R*_f_ = 0.49 (0.5% MeOH in CH_2_Cl_2_); ^1^H NMR (400 MHz, CDCl_3_): *δ* = 7.56 (d, *J = *1.7 Hz, 1H), 7.47 (dd, *J = *7.9, 1.7 Hz, 1H), 7.33 (d, *J = *7.9 Hz, 1H), 7.20 (d, *J = *7.8 Hz, 1H), 7.09 (dd, *J = *7.7, 1.6 Hz, 1H), 6.99 (d, *J = *1.5 Hz, 1H), 4.54–4.41 (m, 3H), 3.71 (dt, *J* = 14.1, 3.4 Hz, 1H), 3.30–3.12 (m, 5H), 3.05–2.94 (m, 1H), 2.91 (s, 3H), 2.00–1.80 (m, 4H), 1.19 (dd, *J = *9.0, 7.0 Hz, 6H); ^13^C{^1^H} NMR (101 MHz, CDCl_3_): *δ* = 204.4, 168.4, 166.2, 160.1, 143.1, 140.4, 135.1, 133.7, 130.7, 128.2, 128.1, 126.0, 123.4, 120.1, 108.8, 80.4, 44.9, 43.3, 39.0, 38.2, 37.6, 36.4, 35.5, 34.7, 16.71, 16.69; MS (ESI+): *m/z* 469.11 [M+H]^+^.

#### 4.1.25 2‐Chloro‐4‐(1′‐[2‐hydroxy‐3‐methyl‐2‐{trifluoromethyl}butanoyl]‐2*H*‐spiro[benzofuran‐3,4′‐piperidin]‐6‐yl)‐*N*,*N*‐dimethylbenzamide (*rac‐*8)

Preparation according to general procedure (F) using **16d** (65 mg, 0.14 mmol, 1.0 equiv), 2 mol/L CF_3_TMS in THF (139 µL, 277 µmol, 2.0 equiv), and 1 mol/L TBAF in THF (277 µL, 277 µmol, 2.0 equiv) in THF (5 mL). Further purification was performed by reversed‐phase column chromatography on silica with 5%–95% acetonitrile in water to obtain (*rac*)‐**8** as a colorless solid (26 mg, 35%). *R*_f_ = 0.41 (0.5% MeOH in CH_2_Cl_2_); ^1^H NMR (500 MHz, CD_2_Cl_2_): *δ* = 7.60 (d, *J = *1.8 Hz, 1H), 7.50 (d, *J* = 7.9 Hz, 1H), 7.32 (dd, *J = *7.9, 1.5 Hz, 1H), 7.20 (d, *J* = 8.4 Hz, 1H), 7.14 (d, *J* = 7.7 Hz, 1H), 7.02 (d, *J = *1.8 Hz, 1H), 5.61 (s, 1H), 4.78–4.17 (m, 4H), 3.53–3.00 (m, 5H), 2.87 (s, 3H), 2.49 (septet, *J = *6.2 Hz, 1H), 2.01–1.81 (m, 4H), 1.11 (d, *J = *6.4 Hz, 3H), 1.01–0.76 (m, 3H); ^19^F NMR (471 MHz, CD_2_Cl_2_): *δ* = −70.26, −71.16; ^13^C{^1^H} NMR (126 MHz, CD_2_Cl_2_): *δ* = 167.8, 167.2, 160.3, 142.9, 140.3, 135.4, 133.8, 130.5, 128.1, 127.9, 125.9, 124.6 (q, *J* = 284.9 Hz), 123.3, 119.9, 108.5, 81.1, 80.5, 44.6, 43.3, 37.8, 35.9, 34.3, 31.0, 16.4 (d, *J* = 63.1 Hz); Accurate mass (ESI): *m/z* calculated for C_27_H_30_ClF_3_N_2_O_4_ [M+H]^+^: 539.19190; found: 539.1922.

#### 4.1.26 Chiral Separation and Enantiomer Characterization of (*rac*)‐7

##### 4.1.26.1 Chiral HPLC

Analytical and semipreparative HPLC were performed on a Shimadzu Prominence with an SPD20A UV/Vis (*λ* = 240/280 nm) detector (Shimadzu, Duisburg, Germany). The stationary phases for analytical determination were Lux 5 µm i‐Amylose‐1 (250 × 4.6 mm), and  the stationary phase used for semipreparative purification was Lux 5 µm i‐Amylose‐1 (250 × 10 mm), both from Phenomenex (Aschaffenburg, Germany). The eluent was a mixture of acetonitrile and an aqueous formic acid solution (0.1%). Flow rates were set to 1 and 5 mL/min. An isocratic mixture with 64% acetonitrile over 70 min was used (analytical and semipreparative purification).

Chiral HPLC analysis of (*rac*)‐**7** (Figure S5) at 254 and 280 nm revealed a peak ratio 52:48 (first:second peak) and 53:47 (first:second peak), demonstrating that (*R*)‐**7** (26 min) was eluted before (*S*)‐**7** (36 min), respectively.

##### 4.1.26.2 Single‐Crystal X‐Ray Diffraction

Single‐crystal diffraction data were collected at −100 °C on a STOE *IPDS* II two‐circle diffractometer equipped with a Genix 3D IµS microfocus X‐ray source (*λ* = 0.71073 Å). Data finalization including empirical absorption corrections was done using *CrysAlis PRO* 1.171.42.43a (Rigaku Oxford Diffraction, 2022). The structure was solved using *SHELXT* [30, 31] and refined against |F|^2^ with full‐matrix least‐squares techniques using the program *SHELXL2019*/2 [32]. It was possible to determine the absolute configuration of (*R*)‐**7**. It crystallized as a dimer. The structure on the right‐hand side is partly disordered, while the other structure is optically pure (Figure S6). A CIF file containing the crystallographic information is deposited in the Cambridge Crystallographic Data Centre under deposition code CCDC 2546214 and can be obtained free of charge via www.ccdc.cam.ac.uk/data_request/cif. Crystallographic data and parameters of the diffraction experiments are given in Table S1.

### 4.2 In Vitro Pharmacological Characterization

#### 4.2.1 Cell Culture

HEK293T and HepG2 cells (German Collection of Microorganisms and Cell Cultures, DSMZ) were grown in Dulbecco's modified Eagle's medium (DMEM) high glucose supplemented with 10% fetal bovine serum (FBS), sodium pyruvate (1 mM), penicillin (100 U/mL), and streptomycin (100 µg/mL) at 37 °C and 5% CO_2_.

#### 4.2.2 Plasmids

For the hybrid reporter gene assays, Gal4‐fusion receptor plasmids pFA‐CMV‐hLXRα‐LBD [33] and pFA‐CMV‐hLXRβ‐LBD [33], coding for the hinge region and LBD of the canonical isoform of the respective nuclear receptor, were used. pFR‐Luc (Stratagene) was used as a reporter plasmid. For the full‐length reporter gene assays, full‐length receptor plasmids pCMV6‐XL4‐hLXRa and pCMV6‐AC‐hLXRb (Origene), coding for the full‐length LXR subtypes, were used. ABCA1‐pGL3 was used as a reporter plasmid. pRL‐SV40 (Promega) was used in all assays for the normalization of transfection efficiency and cell growth. pECE‐SV40‐Gal4‐VP16 was a gift from Lea Sistonen (Addgene plasmid #71728 [34]).

#### 4.2.3 Reporter Gene Assays

The day before transfection, HEK293T cells were seeded in 96‐well plates (4 × 10^4^ cells/well). Before transfection, the medium was changed to Opti‐MEM without supplements. Transient transfection was carried out using Lipofectamine LTX reagent (Invitrogen) according to the manufacturer's protocol with the reporter plasmid, pRL‐SV40 (Promega), and the corresponding nuclear receptor plasmid. Five hours after transfection, the medium was changed to Opti‐MEM supplemented with penicillin (100 U/mL) and streptomycin (100 µg/mL) and now additionally containing 0.1% DMSO and the respective test compound or 0.1% DMSO alone as a negative control. Each concentration was tested in duplicate, and each experiment was repeated in at least three biologically independent repeats. Following overnight (14–16 h) incubation with the test compounds, cells were assayed for luciferase activity using the Dual‐Glo Luciferase Assay System (Promega) according to the manufacturer's protocol. Luminescence was measured with a Spark 10 M luminometer (Tecan Deutschland GmbH). Normalization of transfection efficiency and cell growth was done by division of firefly luciferase data by renilla luciferase data multiplied by 1000, resulting in relative light units (RLU). Fold activation was obtained by dividing the mean RLU of the tested compound at a respective concentration by the mean RLU of the 0.1% DMSO control. Relative activation was obtained by dividing the fold activation of the tested compound at a respective concentration by the fold activation of the respective reference agonist (LXRα/β: 1 µM T0901317(**32**)). Toxicity‐adjusted data for compound **2** on full‐length LXRα and full‐length LXRβ were calculated by dividing the relative activation by the relative viability obtained from the cytotoxicity assay at the corresponding concentration. Separate control experiments to exclude nonspecific reporter gene activation or VP16‐mediated effects were performed following the same procedure in the absence of a nuclear receptor expression plasmid (pFR‐Luc, pRL‐SV40, and pECE‐SV40‐Gal4‐VP16 only). EC_50_/IC_50_ values and the standard errors thereof were calculated with the mean fold activation values by OriginPro, Version 2026 (OriginLab Corporation, Northampton, MA, USA), fitting a dose–response curve with variable Hill slope (Levenberg–Marquardt algorithm). All assays were validated with the above‐mentioned reference agonist, which yielded EC_50_ values in agreement with the literature [25, 26]: T0901317 (**32**): Gal4‐LXRα 0.47 ± 0.02 μM (109% ± 3%), Gal4‐LXRβ 0.76 ± 0.10 μM (135% ± 12%), full‐length LXRα 0.021 ± 0.004 μM (104% ± 3%), full‐length LXRβ 0.033 ± 0.010 μM (101% ± 8%); LXR‐623 (**33**): Gal4‐LXRα 1.6 ± 0.2 μM (40% ± 6%), Gal4‐LXRβ 1.4 ± 0.1 μM (50% ± 4%), full‐length LXRα 0.063 ± 0.003 μM (58% ± 1%), full‐length LXRβ 0.15 ± 0.02 μM (77% ± 2%). Dose–response curves for reference agonists T0901317 (**32**) and LXR‐623 (**33**) can be found in the Supporting Information (Figure S4).

#### 4.2.4 Cytotoxicity

HEK293T cells were seeded in DMEM high glucose supplemented with sodium pyruvate (1 mM), penicillin (100 U/mL), streptomycin (100 µg/mL), and 10% FBS in 96‐well plates (4 × 10^4^ cells/well). After 24 h, the cells were incubated with compound **2**, compound (*rac*)‐**7** (final concentrations 0.03, 0.1, 0.3, 1, 3, 10, 30, and 100 µM), bexarotene (100 µM) as a positive control, or 0.1% DMSO in Opti‐MEM as a negative control. After 24 h, the medium was aspirated, and the cells were incubated with 100 µM resazurin solution in Opti‐MEM (prepared from a 0.15 mg/mL resazurin stock in Dulbecco's phosphate‐buffered saline (DPBS)) for 60 min. The metabolic activity was measured by assessing the conversion of resazurin to resorufin, which was quantified by measuring absorbance at 570 nm and a reference wavelength of 600 nm on a Tecan Spark 10 M luminometer (Tecan Deutschland GmbH). Each experiment was repeated three times in duplicates.

#### 4.2.5 Metabolic Stability Assay

The dissolved test compound (5 µL, final concentration 10 µM in phosphate buffer (0.1 M, pH 7.4)) was preincubated at 37 °C in 432 µL of phosphate buffer (0.1 M, pH 7.4) together with a 50 µL NADPH regenerating system (30 mM glucose‐6‐phosphate, 4 U/mL glucose‐6‐phosphate dehydrogenase, 10 mM NADP, and 30 mM MgCl_2_). After 5 min, the reaction was started by the addition of 13 µL of microsome mix from the liver of Sprague−Dawley rats (Invitrogen; 20 mg protein/mL in 0.1 M phosphate buffer) in a shaking water bath at 37 °C. The reaction was stopped by addition of 500 µL ice‐cold methanol at 0, 15, 30, and 60 min. The samples were centrifuged at 10^4^ g for 5 min at 4 °C, and the test compounds were quantified by HPLC from the supernatants. The mobile phase is adapted to the test compound in a range of 40%–90% acetonitrile and 10%–60% of H_2_O with 0.1% formic acid; flow rate: 1 mL/min; stationary phase: Purospher STAR 120 RP‐18e, 5 µm, 125 × 4.0 mm column (Merck KGaA, Darmstadt, Germany), precolumn: Purospher STAR RP‐18, 5 µm, 4.0 × 4.0 mm (Merck KGaA, Darmstadt, Germany); detection wavelength: 254 and 280 nm; injection volume: 50 µL. Control samples were performed to check the test compound's stability in the reaction mixture: The first control was without NADPH, which is needed for the enzymatic activity of the microsomes, the second control was with inactivated microsomes (incubated for 20 min at 90 °C), and the third control was without test compound (to determine the baseline). The amounts of the test compound were quantified by an external calibration curve, where data are expressed as the mean ± SEM of single determinations obtained in three independent experiments.

#### 4.2.6 Target Gene Quantification

HepG2 cells were seeded in 6‐well plates (1 × 10^6^ per well). After 24 h, the medium was changed to Opti‐MEM supplemented with 1% charcoal‐stripped FBS, penicillin (100 U/mL), streptomycin (100 µg/mL), and L‐glutamine (2 mM). After an additional 24 h, cells were incubated with the test compounds dissolved in the same medium with 0.1% DMSO or medium with 0.1% DMSO alone as a negative control for 24 h, harvested, washed with cold phosphate‐buffered saline (PBS), and then directly used for the next step. Total RNA was extracted from HepG2 cells using the total RNA Mini Kit (R6834−02, Omega Bio‐Tek, Inc.), and the same amount of RNA per sample was then reverse transcribed into cDNA with the High‐Capacity cDNA Reverse Transcription Kit (4368814, Thermo Fisher Scientific, Inc.). Target gene expression was then studied by quantitative real‐time PCR (qRT‐PCR) analysis with a qTower3/G (Analytik Jena GmbH & Co. KG) using PowerSYBRGreen (Life Technologies). Each sample was set up in duplicates and repeated in four independent experiments. Data were analyzed by the comparative ΔΔ*C*_t_ method with glyceraldehyde 3‐phosphate dehydrogenase (GAPDH) as the reference gene. The following primers were used (human genes): hGAPDH fwd: 5′‐ATA TGA TTC CAC CCA TGG CA‐3′, rev: 5′‐GAT GAT GAC CCT TTT GGC TC‐3′; hABCA1 fwd: 5′‐TTCGCTCTGAGATGAGCACCA‐3′, rev: 5′‐TTTCAAGCGGGCATAGAACCA‐3′; hABCG1 fwd: 5′‐ACGCAGTTCTGCATCCTCTT‐3′, rev: 5′‐CGGAGTTGCTCAAGACCTTC‐3′; hFAS fwd: 5′‐ATCGGCTCCACCAAGTCCAA‐3′, rev: 5′‐CCGCGATGTCGTTCAGCA‐3′; hSREBP1c fwd: 5′‐GGAGGGGTAGGGCCAACGGCCT‐3′, rev: 5′‐CATGTCTTCGAAAGTGCAATCC‐3′.

#### 4.2.7 Lipogenesis Assay

HepG2 cells were seeded in collagen G‐coated 96‐well plates (1.0 × 10^4^ per well) and incubated for 24 h. Then, the cells were incubated with the test compounds dissolved in culture medium with 0.1% DMSO or medium with 0.1% DMSO alone as a negative control for 72 h. The cells were washed with PBS (2 × 50 µL), fixed with 10% formalin solution (50 µL) for 15 min, and washed again with H_2_O (2 × 50 µL) followed by 60% *i*‐PrOH (50 µL). Neutral lipid droplets were stained with 0.3% Oil Red O solution (in 60% *i*‐PrOH, 50 µL) for 10 min. After washing the cells quickly with 60% *i*‐PrOH (50 µL) and PBS (2 × 50 µL), they were visualized under a BZ‐X810 microscope (KEYENCE, Frankfurt, Germany) using a 40x objective. The images were analyzed using ImageJ software (v1.54) according to the literature method [35].

### 4.3 Computational Methods

#### 4.3.1 Scaffold Hopping

The X‐ray structure of LXR in complex with compound **2** was prepared using the QuickPrep routine in MOE, version 2022.02. Subsequently, the bipepiridine scaffold in compound **2** was selected manually, and the Scaffold Replacement routine with default settings and without filters was used to generate suggestions from the default linker library (40,626 linkers). 456 structures were created and inspected manually, paying attention to synthetic accessibility.

#### 4.3.2 Molecular Docking

Molecular docking was performed using AutoDock Vina [20], as implemented in Chimera, version 1.19 [36]. The cocrystal structures of **2** in LXRα and LXRβ (PDB ID: 5HJS and 5HJP [12]) were used as template and the cocrystallized ligand was removed. The protein and ligand structures were pretreated using the “Dock prep” function with default settings, including the deletion of solvent, addition of hydrogens, and Gasteiger charges. The docking grid was defined as a box around the ligand with the center coordinates (20.5899, −16.6750, 19.3280) for LXRα (−4.00777, −6.21816, 21.7257) and for LXRβ and a dimension of (25.4397, 13.5801, 16.3231) for LXRα and (18.5438, 11.3530, 16.4443) for LXRβ. The exhaustiveness was set to 8, and the top 10 scoring binding modes were visualized in ChimeraX, version 1.10 [37]. To validate the method, **2** was redocked five times into the crystal structure using identical settings, yielding low root mean square deviation (RMSD) values (mean RMSD = 0.807 ± 0.002 Å for LXRα and 0.704 ± 0.080 Å for LXRβ). Compound (*S*)‐**7** was docked five times into the crystal structures, and in all cases, the poses shown in Figure 2 (RMSD = 0.489 ± 0.161 Å for LXRα and 0.376 ± 0.160 Å for LXRβ) were found to be the top‐ranked binding mode.

## Funding

This study was supported by the Hans und Ria Messer Stiftung (Z_HMS_11/022 Goethe Universität FFM_Dr. Heitel). Annette Kärcher was supported individually by Stiftung Polytechnische Gesellschaft Frankfurt and Detlef Hübner Stiftung.

## Conflicts of Interest

The authors declare no conflicts of interest.

## Supporting information

Supplementary Material

## Data Availability

The data that support the findings of this study are available in the Supporting Information of this article.
