# Supplementary material for: Scaffold Hopping Provides Liver X Receptor Agonist With Preference for the β Subtype
Source: ChemMedChem. 2026 Oct 7;21(19):e70509. doi: 10.1002/cmdc.70509 (PMC13646660; doi:10.1002/cmdc.70509)
Supplement: Supplementary file 1 [file CMDC-21-e70509-s001.pdf]

Supporting Information

Scaffold Hopping Provides Liver X Receptor Agonist with Preference for the β Subtype

Annette Kärcher[1], Fiona L. Motel[1], Felix F. Lillich[1], Astrid Kaiser[1], Ewgenij Proschak[1], and Pascal Heitel[1]*

[1] Institute of Pharmaceutical Chemistry, Goethe University Frankfurt, Max-von-Laue-Strasse 9, 60438 Frankfurt, Germany

* Pascal Heitel, email: heitel@pharmchem.uni-frankfurt.de

Table of Contents

1. Supporting Figures & Tables 4

2. Synthesis of reference (*rac*)-**2** and photocatalyst **31** 8

3. NMR Spectra & HPLC Chromatograms 9

2-Chloro-4-(1'-[2-hydroxy-3-methyl-2-{trifluoromethyl}butanoyl]-[4,4'-bipiperidin]-1-yl)-*N*,*N*-dimethylbenzamide (*rac*-**2**) 9

4-Bromo-2-chloro-*N*,*N*-dimethylbenzamide (**11**) 11

tert-Butyl 4-(3'-chloro-4'-[dimethylcarbamoyl]-[1,1'-biphenyl]-4-yl)piperazine-1-carboxylate (**14a**) 12

3-Chloro-*N*,*N*-dimethyl-4'-(4-[3-methyl-2-oxobutanoyl]piperazin-1-yl)-[1,1'-biphenyl]-4-carboxamide (**16a**) 13

3-Chloro-4'-(4-[2-hydroxy-3-methyl-2-{trifluoromethyl}butanoyl]piperazin-1-yl)-*N*,*N*-dimethyl-[1,1'-biphenyl]-4-carboxamide (*rac*-**5**) 14

2-Chloro-*N*,*N*-dimethyl-4-(4,4,5,5-tetramethyl-1,3,2-dioxaborolan-2-yl)benzamide (**12**) 17

tert-Butyl (3a*R*,6a*S*)-5-(5-bromopyridin-2-yl)hexahydropyrrolo[3,4-*c*]pyrrole-2(1*H*)-carboxylate (**13b**) 18

tert-Butyl (3a*R*,6a*S*)-5-(5-[3-chloro-4-{dimethylcarbamoyl}phenyl]pyridin-2-yl)hexahydropyrrolo[3,4-*c*]pyrrole-2(1*H*)-carboxylate (**14b**) 19

2-Chloro-*N*,*N*-dimethyl-4-(6-[{3a*R*,6a*S*}-5-{3-methyl-2-oxobutanoyl}hexahydropyrrolo[3,4-*c*]pyrrol-2{1*H*}-yl]pyridin-3-yl)benzamide (**16b**) 20

2-Chloro-4-(6-[{3a*R*,6a*S*}-5-{2-hydroxy-3-methyl-2-(trifluoromethyl)butanoyl}hexahydropyrrolo[3,4-*c*]pyrrol-2{1*H*}-yl]pyridin-3-yl)-*N*,*N*-dimethylbenzamide (*rac*-**6**) 21

([5-Bromo-1*H*-inden-3-yl]oxy)(*tert*-butyl)dimethylsilane (**21**) 24

tert-Butyl 5-bromo-3-oxo-2,3-dihydrospiro[indene-1,4'-piperidine]-1'-carboxylate (**23**) 25

tert-Butyl 5-bromo-2,3-dihydrospiro[indene-1,4'-piperidine]-1'-carboxylate (**13c**) 26

tert-Butyl 5-(3-chloro-4-[dimethylcarbamoyl]phenyl)-2,3-dihydrospiro[indene-1,4'-piperidine]-1'-carboxylate (**14c**) 27

2-Chloro-*N*,*N*-dimethyl-4-(1'-[3-methyl-2-oxobutanoyl]-2,3-dihydrospiro[indene-1,4'-piperidin]-5-yl)benzamide (**16c**) 28

2-Chloro-4-(1'-[2-hydroxy-3-methyl-2-{trifluoromethyl}butanoyl]-2,3-dihydrospiro[indene-1,4'-piperidin]-5-yl)-*N*,*N*-dimethylbenzamide (*rac*-**7**) 29

tert-Butyl 4-hydroxy-4-([phenylsulfinyl]methyl)piperidine-1-carboxylate (**26**) 32

tert-Butyl 3-hydroxy-4-methylenepiperidine-1-carboxylate (**27**) 33

tert-Butyl 4-(chloromethyl)-3,6-dihydropyridine-1(2*H*)-carboxylate (**28**) 34

tert-Butyl 4-([5-bromo-2-iodophenoxy]methyl)-3,6-dihydropyridine-1(2*H*)-carboxylate (**30**) 35

2,4,6-Tris(diphenylamino)-5-fluoroisophthalonitrile (**31**) 36

tert-Butyl 6-bromo-2*H*-spiro[benzofuran-3,4'-piperidine]-1'-carboxylate (**13d**) 37

tert-Butyl 6-(3-chloro-4-[dimethylcarbamoyl]phenyl)-2*H*-spiro[benzofuran-3,4'-piperidine]-1'-carboxylate (**14d**) 38

2-Chloro-*N,N*-dimethyl-4-(1'-[3-methyl-2-oxobutanoyl]-2*H*-spiro[benzofuran-3,4'-piperidin]-6-yl)benzamide (**16d**) 39

2-Chloro-4-(1'-[2-hydroxy-3-methyl-2-{trifluoromethyl}butanoyl]-2*H*-spiro[benzofuran-3,4'-piperidin]-6-yl)-*N,N*-dimethylbenzamide (*rac*-**8**) 40

4. Supplementary References 43

1. Supporting Figures & Tables

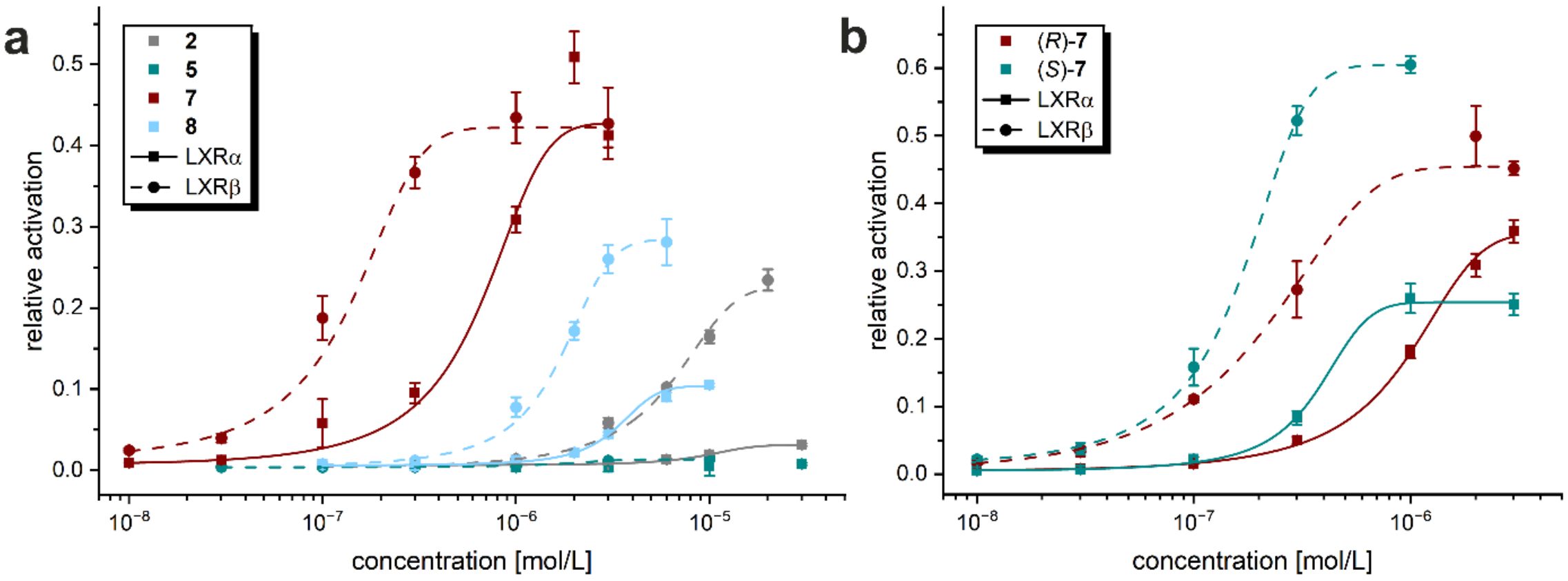

Figure S1. Dose-response curves of scaffolds **5**, **7** and **8** compared to the parent compound **2**. Panel (a) shows the activity of racemic **7**, and the enantiomers are displayed in panel (b). Data are the mean activation ± SEM relative to 1 µM T0901317 of $N \geq 3$.

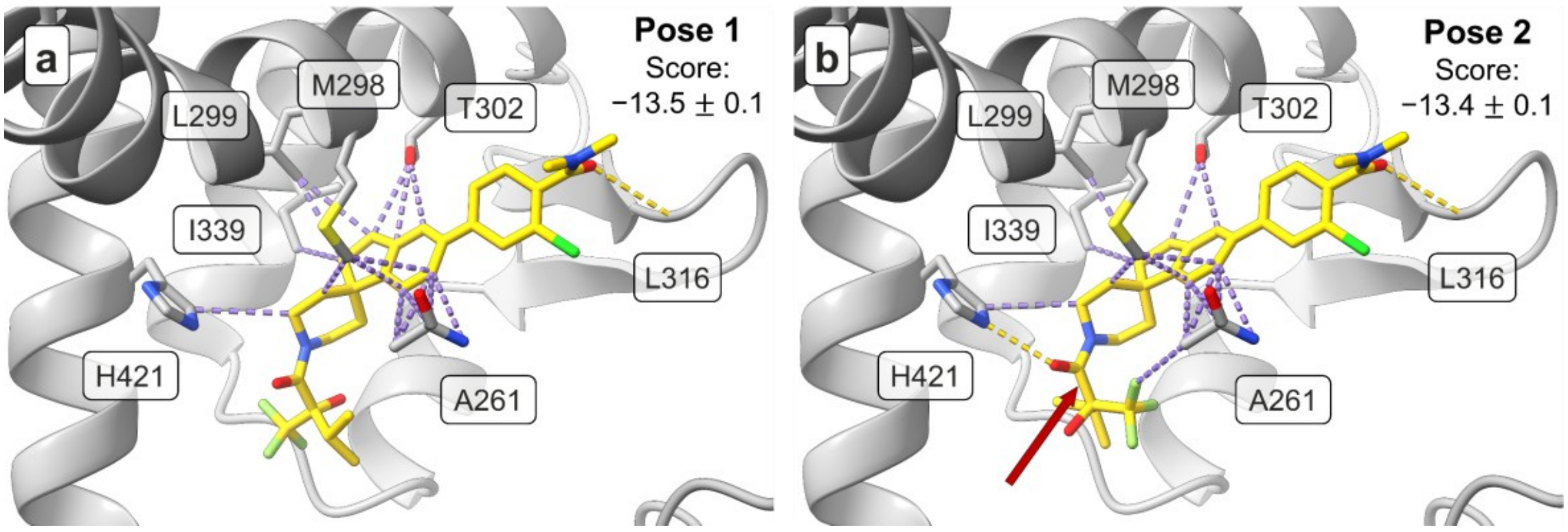

Figure S2. Molecular docking of the improved scaffold (*S*)-**7** in the ligand-binding domain of LXRα (PDB ID 5HJS[1]) using AutodockVina[2] provided two poses with low binding scores. The major difference between them is an approximately 180 ° rotation around the C-C bond indicated by a red arrow. In contrast, this conformational flexibility has not been observed in the docking poses in LXRβ (PDB ID 5HJP[1]).

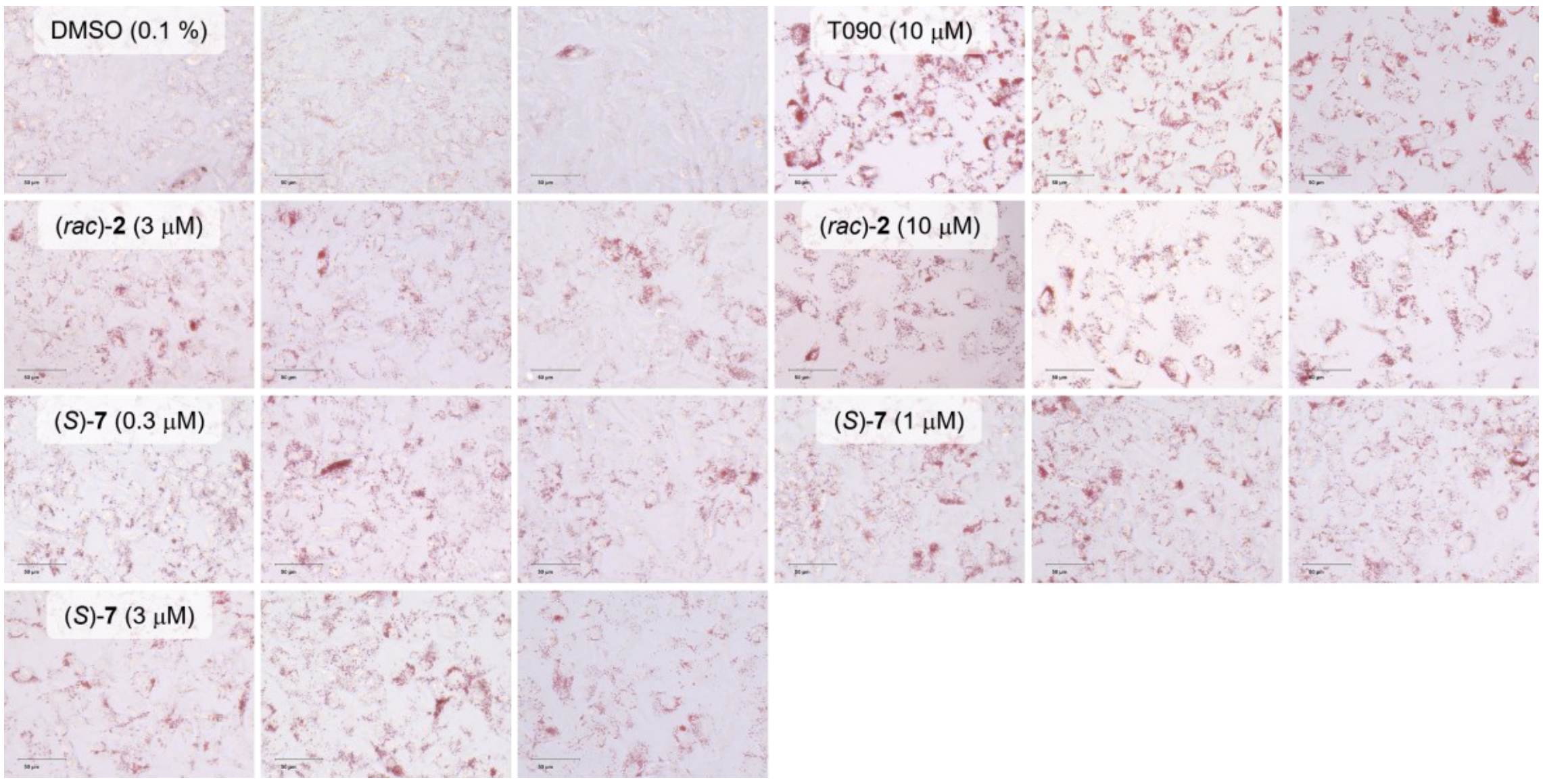

Figure S3. Complete set of images obtained from Oil Red O staining in HepG2 cells after 72 h incubation with indicated compounds (40x magnification): DMSO 0.1% as negative control, pan LXR full agonist T0901317 (**32**, 10 µM) as positive control or the LXRβ-selective agonists **2** and (*S*)-**7**. Each image was obtained from an individual biological experiment. $N = 3$.

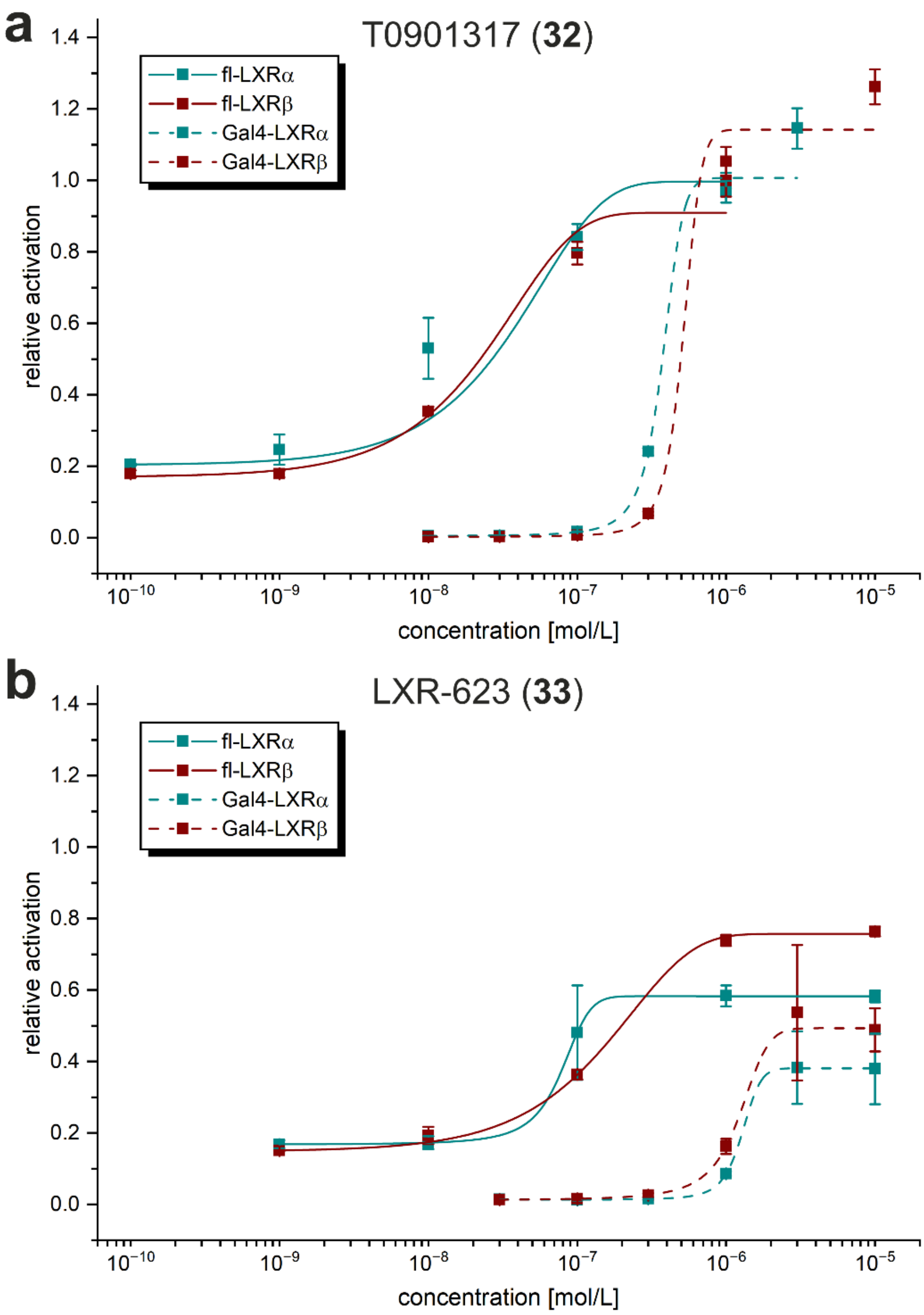

Figure S4. Dose-response curves of the literature LXR agonists (a) T0901317 (**32**) and (b) LXR-623 (**33**) in the Gal4 hybrid and full-length LXR reporter gene assay. Data are the mean activation ± SEM relative to 1 µM T0901317 of $N \geq 3$.

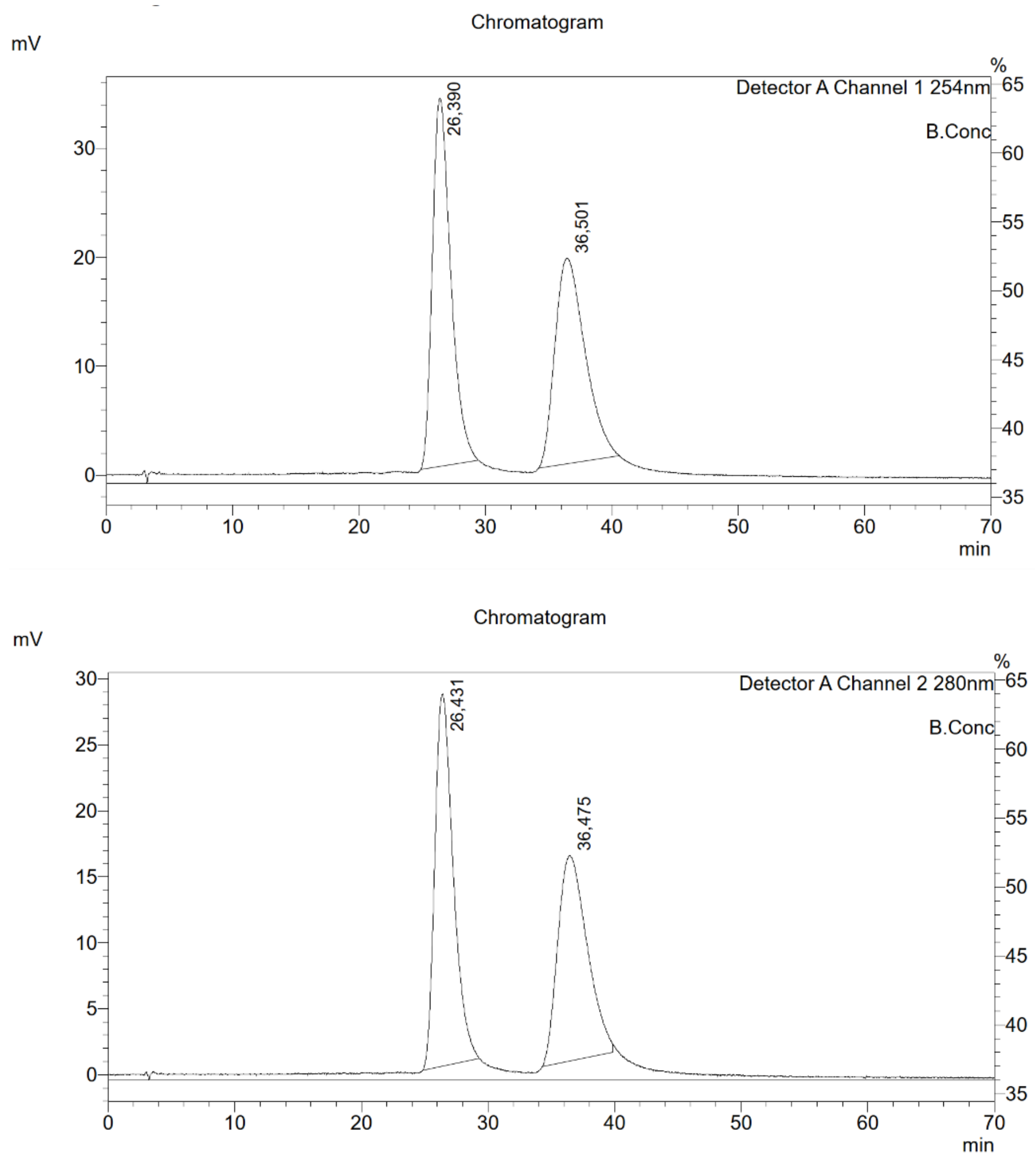

Figure S5. HPLC chromatograms of HPLC analysis (i-Amylose-1) at 254 nm and 280 nm of (*rac*)-**7** containing 52 % *R* enantiomer and 48 % *S* enantiomer, and 53 % *R* enantiomer and 47 % *S* enantiomer, respectively.

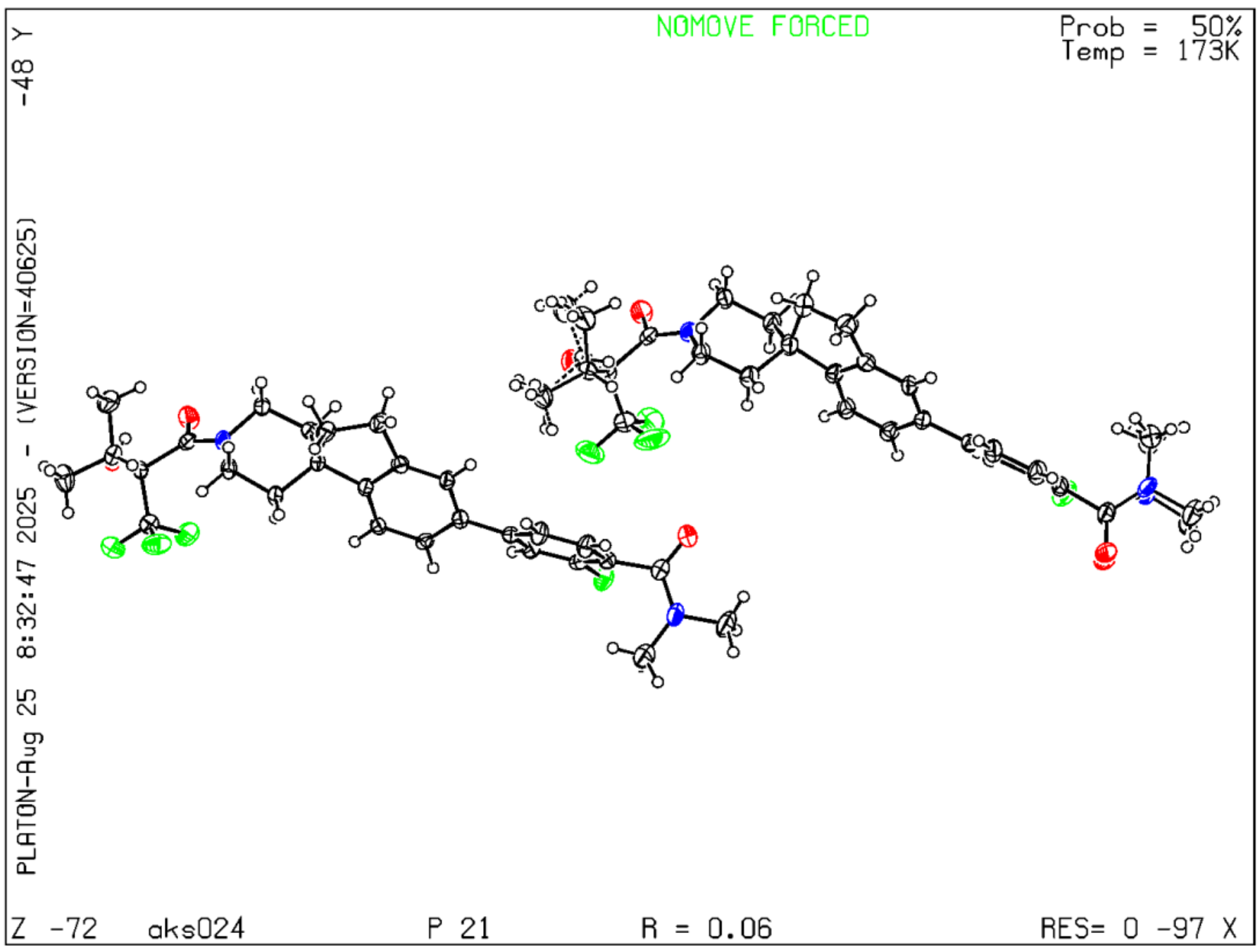

Figure S6. Crystal structure of (*R*)-**7** crystallized as a dimer.

Table S1. Crystallographic data of (*R*)-**7**.

Chemical formula	$C_{28}H_{32}ClF_3N_2O_3$
M_r	537.00
Crystal system, space group	Monoclinic, $P2_1$
Temperature (K)	173
a, b, c (Å)	9.8006 (7), 12.6046 (7), 21.5842 (11)
b(°)	92.560 (6)
V (Å^3)	2663.7 (3)
Z	4
F(000)	1128
D_x(Mg m^{-3})	1.339
Radiation type	Mo *Kα*
No. of reflections for cell measurement	2639
θ range (°) for cell measurement	2.7–24.7
μ (mm^{-1})	0.20
Crystal shape	Thin plate
Color	Colorless
Crystal size (mm)	0.25 × 0.13 × 0.08
Diffractometer	STOE *IPDS* II two-circle-diffractometer
Radiation source	Genix 3D IμS microfocus X-ray source
Monochromator	Mirror
Detector resolution (pixels mm^{-1})	6.67
Scan method	ω scans
Absorption correction	Multi-scan<br>*CrysAlis PRO* 1.171.42.43a (Rigaku Oxford Diffraction, 2022) Empirical absorption correction using spherical harmonics, implemented in SCALE3 ABSPACK scaling algorithm.
T_{min}, T_{max}	0.957, 1.000
No. of measured, independent and observed [$I > 2\sigma(I)$] reflections	20730, 10300, 7439
R_{int}	0.077
$(\sin \theta/\lambda)_{max}$ (Å^{-1})	0.617
Range of *h*, *k*, *l*	$h = -12 \rightarrow 12$, $k = -15 \rightarrow 15$, $l = -22 \rightarrow 26$
Refinement on	F^2
$R[F^2 > 2\sigma(F^2)]$, $wR(F^2)$, S	0.058, 0.130, 1.01
No. of reflections	10300
No. of parameters	731
No. of restraints	61
H-atom treatment	H atoms treated by a mixture of independent and constrained refinement
Weighting scheme	$w = 1/[\sigma^2(F_o^2) + (0.0282P)^2]$ where $P = (F_o^2 + 2F_c^2)/3$
$(\Delta/\sigma)_{max}$	0.001
$\Delta\rho_{max}$, $\Delta\rho_{min}$ (e Å^{-3})	0.52, −0.25
Absolute structure	Flack x determined using 2449 quotients [(I+)-(I-)]/[(I+)+(I-)][3]
Absolute structure parameter	-0.03 (6)

Computer programs: *X-AREA* (Stoe & Cie, 2001), *CrysAlis PRO* 1.171.42.43a (Rigaku OD, 2022), *SHELXT*,[4,5] *SHELXL2019*/2,[6] IUCr *publCIF* online service.

2. Synthesis of reference (rac)-2 and photocatalyst 31

2-Chloro-4-(1'-[2-hydroxy-3-methyl-2-{trifluoromethyl}butanoyl]-[4,4'-bipiperidin]-1-yl)-*N*,*N*-dimethylbenzamide (*rac*-2). Preparation according to the literature.[12] R_f = 0.35 (CH_2Cl_2/MeOH 99:0.5); ^{1}H NMR (500 MHz, $CDCl_3$): δ = 7.15 (d, *J* = 8.5 Hz, 1H), 6.95 – 6.75 (m, 2H), 5.68 (s, 1H), 4.74 (s, 1H), 4.31 (s, 1H), 3.73 (d, *J* = 12.3 Hz, 2H), 3.10 (s, 3H), 2.88 (s, 3H), 2.73 (s, 3H), 2.44 (s, 1H), 1.91 – 1.73 (m, 4H), 1.50 – 1.15 (m, 7H), 1.11 (d, *J* = 6.4 Hz, 3H), 0.83 (s, 3H); ^{19}F NMR (471 MHz, $CDCl_3$): δ = −69.73, −70.90, −73.16; ^{13}C{^{1}H} NMR (126 MHz, $CDCl_3$): δ = 168.9, 166.8, 143.3, 131.4, 128.7, 125.62, 125.62 (q), 116.0, 114.4, 81.0, 49.3, 46.3, 40.8, 40.4, 38.3, 34.8, 31.0, 29.44, 29.37, 28.6, 17.1, 16.7; Accurate mass (ESI): *m/z* calculated for $C_{25}H_{36}ClF_3N_3O_3$ $[M+H]^+$: 518.2392; found: 518.2390.

2,4,6-Tris(diphenylamino)-5-fluoroisophthalonitrile (31). Diphenylamine (1.27 g, 7.50 mmol, 3.8 equiv) dissolved in dry THF (10 mL) was placed under argon atmosphere. NaH (270 mg, 11.3 mmol, 5.6 equiv, 60 % dispersion in mineral oil) was added and the mixture was stirred at 50 °C for 30 minutes. After cooling to room temperature, 2,4,5,6-tetrafluoroisophthalonitrile (400 mg, 2.00 mmol, 1.0 equiv) was added and the mixture was stirred at room temperature for 16 h. Water was added and the organic components were extracted three times with CH_2Cl_2 (10 mL each). The combined organic layers were washed with brine (10 mL), dried over Na_2SO_4 and the solvent was evaporated. Further purification was performed by column chromatography on silica with *n*-hexane/EtOAc 3:1 to obtain 2,4,6-tris(diphenylamino)-5-fluoroisophthalonitrile (**31**) as a yellow solid (531 mg, 41 %).[7] R_f = 0.28 (*n*-hexane/EtOAc 3:1); ^{1}H NMR (300 MHz, $CDCl_3$): δ = 7.34 – 7.24 (m, 12H), 7.15 – 6.97 (m, 18H); ^{19}F NMR (282 MHz, $CDCl_3$): δ = −121.26; ^{13}C{^{1}H} NMR (75 MHz, $CDCl_3$): δ = 152.6 (d, *J* = 258.9 Hz), 152.0 (d, *J* = 3.7 Hz), 145.7, 145.5 (d, *J* = 1.3 Hz), 143.2 (d, *J* = 11.4 Hz), 129.6, 129.5, 124.7, 124.2, 122.89, 122.85, 112.8 (d, *J* = 4.0 Hz), 109.3 (d, *J* = 3.7 Hz); MS (ESI+): *m/z* 670.05 $[M+Na]^+$.

3. NMR Spectra & HPLC Chromatograms

2-Chloro-4-(1'-[2-hydroxy-3-methyl-2-{trifluoromethyl}butanoyl]-[4,4'-bipiperidin]-1-yl)-*N*,*N*-dimethylbenzamide (*rac*-2)

^{1}H NMR (500 MHz, $CDCl_3$)

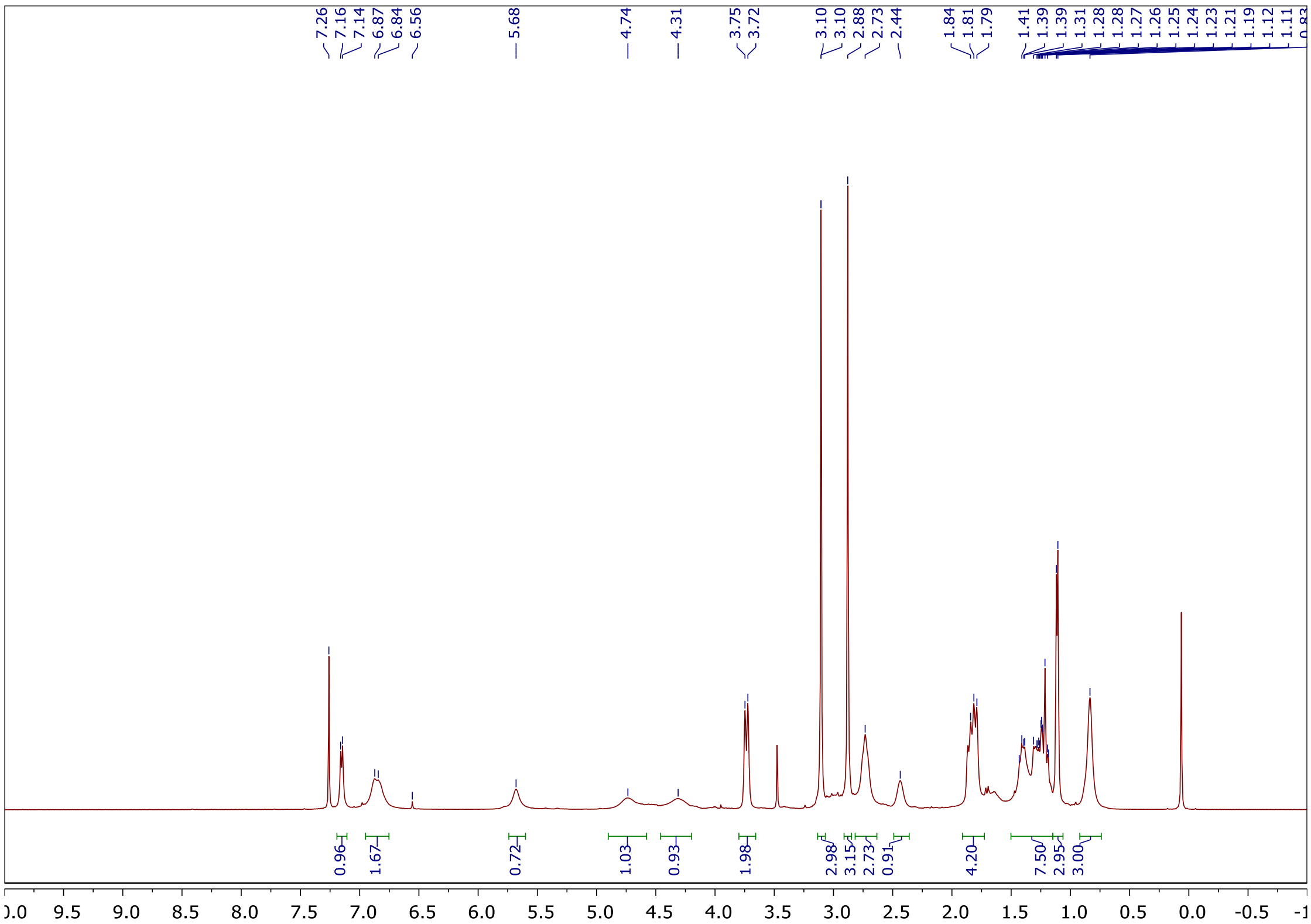

^{13}C NMR (126 MHz, $CDCl_3$)

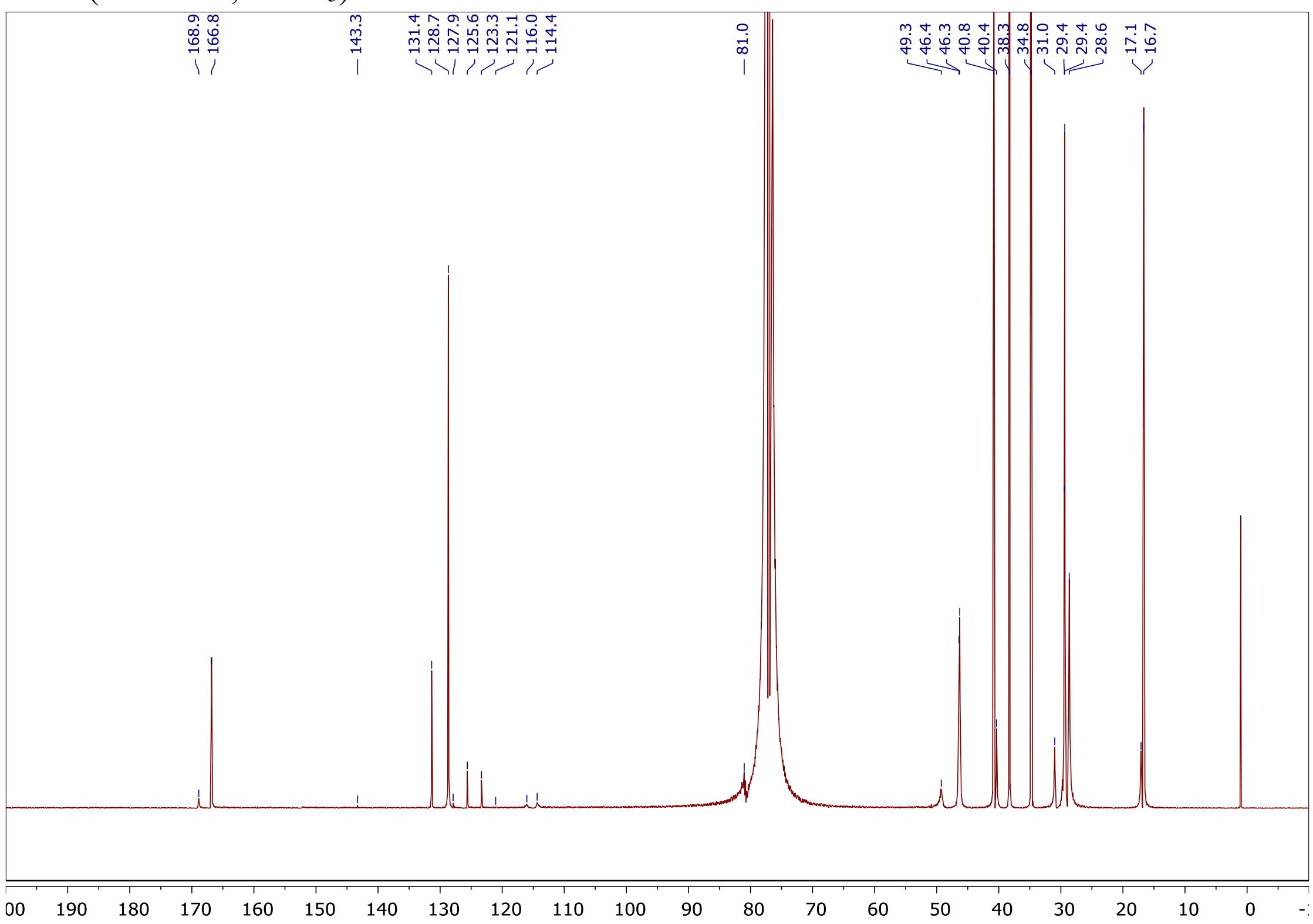

^{19}F NMR (471 MHz, $CDCl_3$)

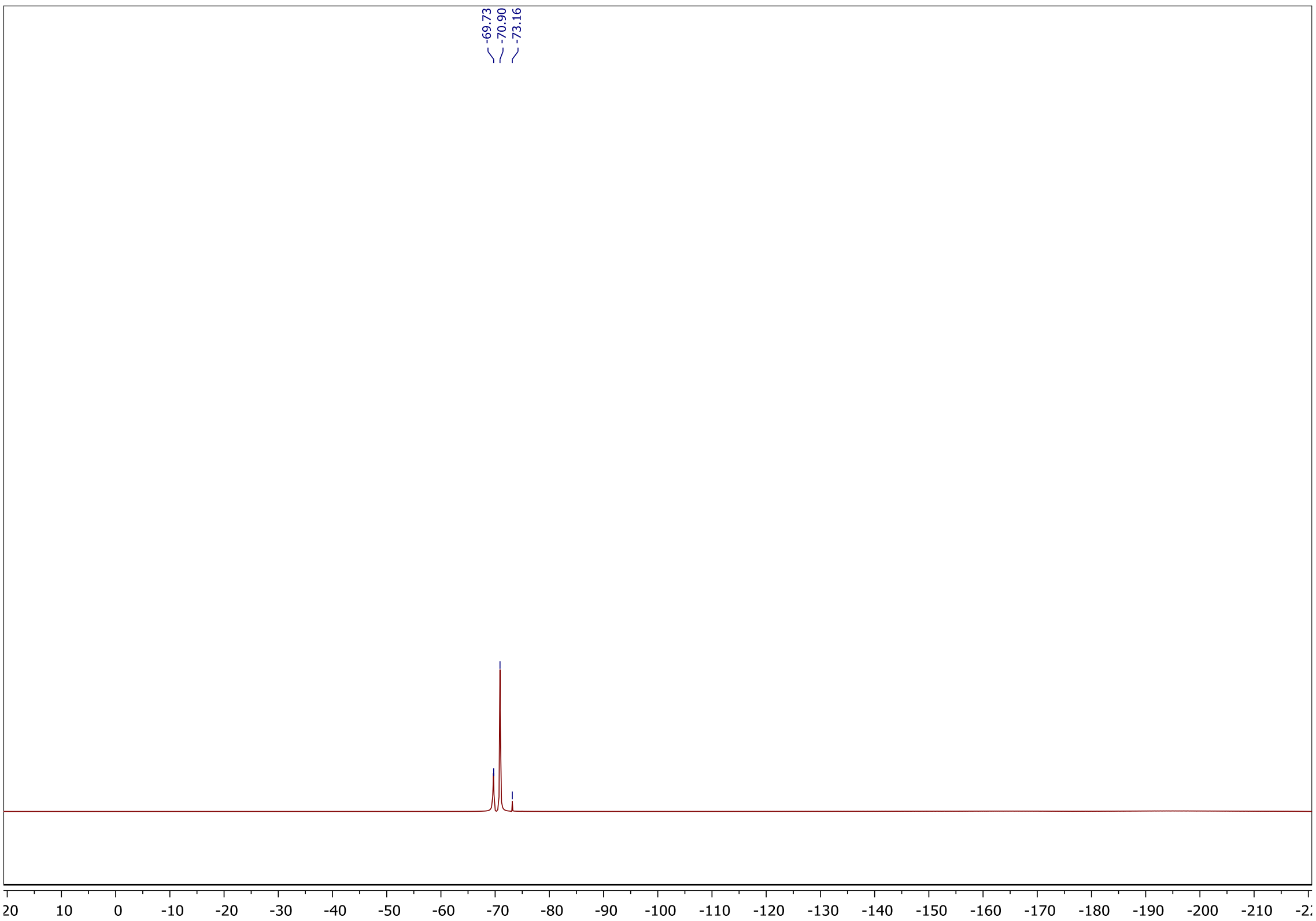

HPLC Traces

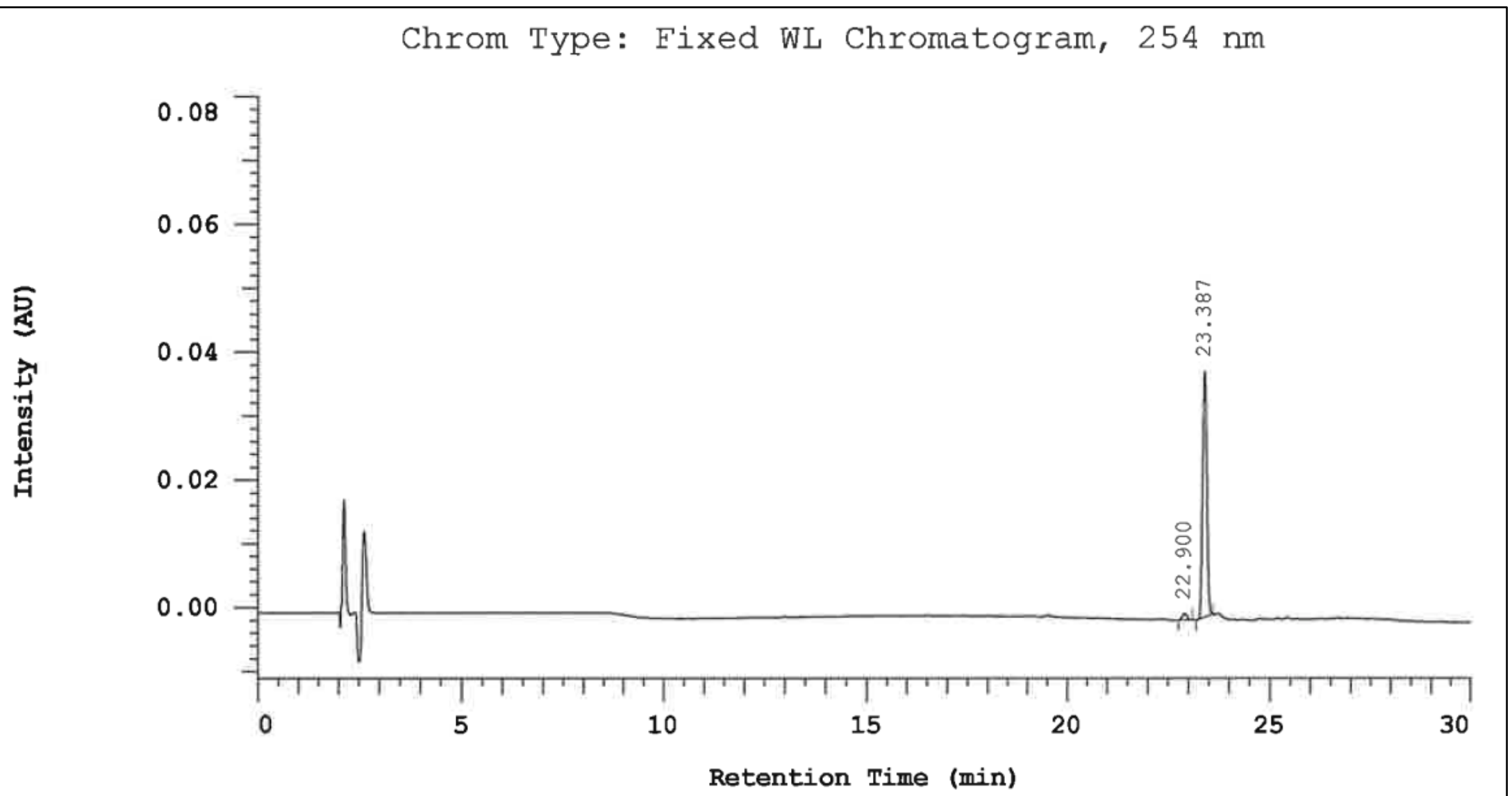

No.	RT	Area	Area %	Height
1	22.900	3832	2.748	498
2	23.387	135638	97.252	19223
		139470	100.000	19721

Peak rejection level: 0

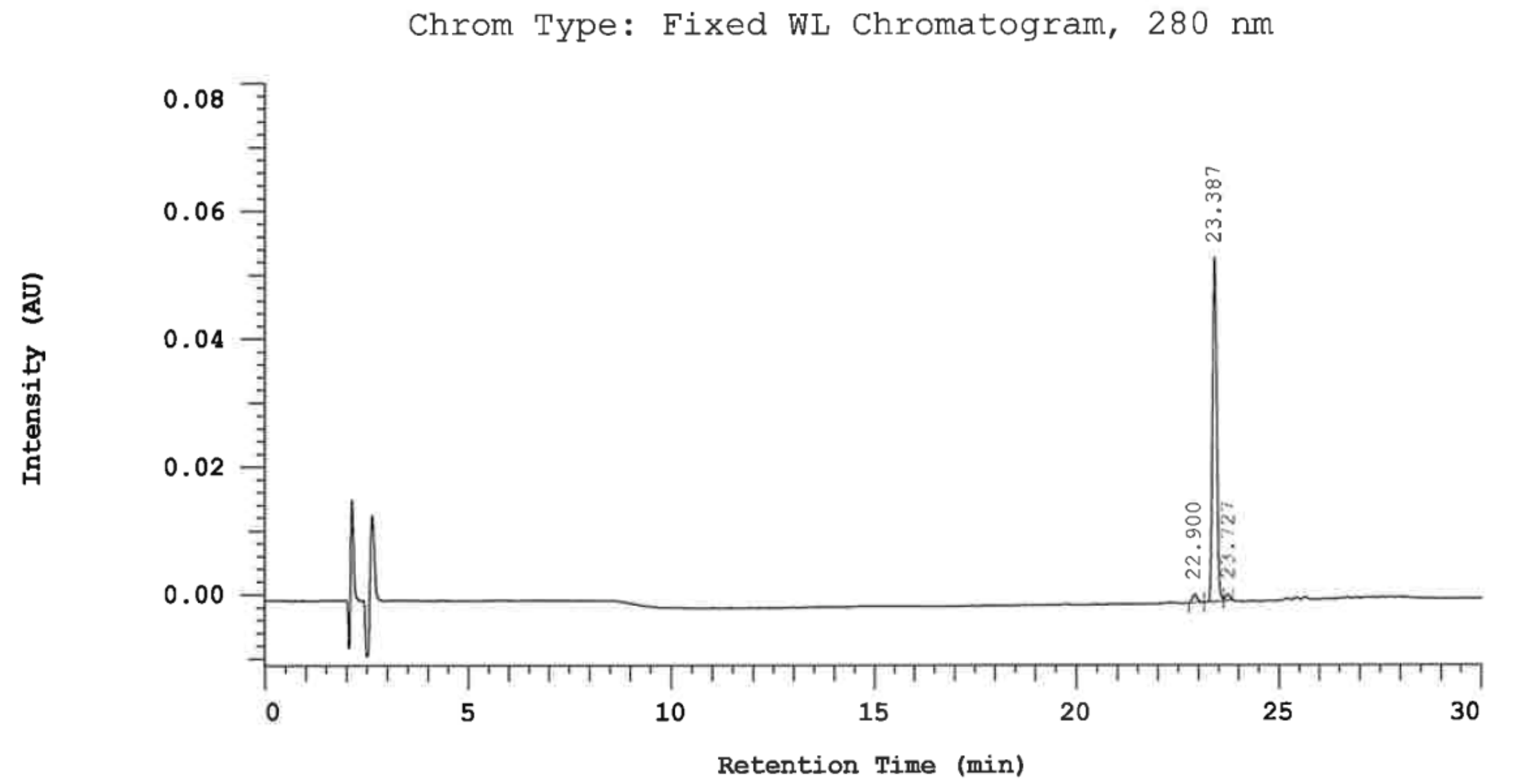

No.	RT	Area	Area %	Height
1	22.900	4909	2.438	629
2	23.387	192240	95.479	26884
3	23.727	4192	2.082	492
		201341	100.000	28005

Peak rejection level: 0

4-Bromo-2-chloro-*N*,*N*-dimethylbenzamide (11)

^{1}H NMR (300 MHz, $CDCl_3$)

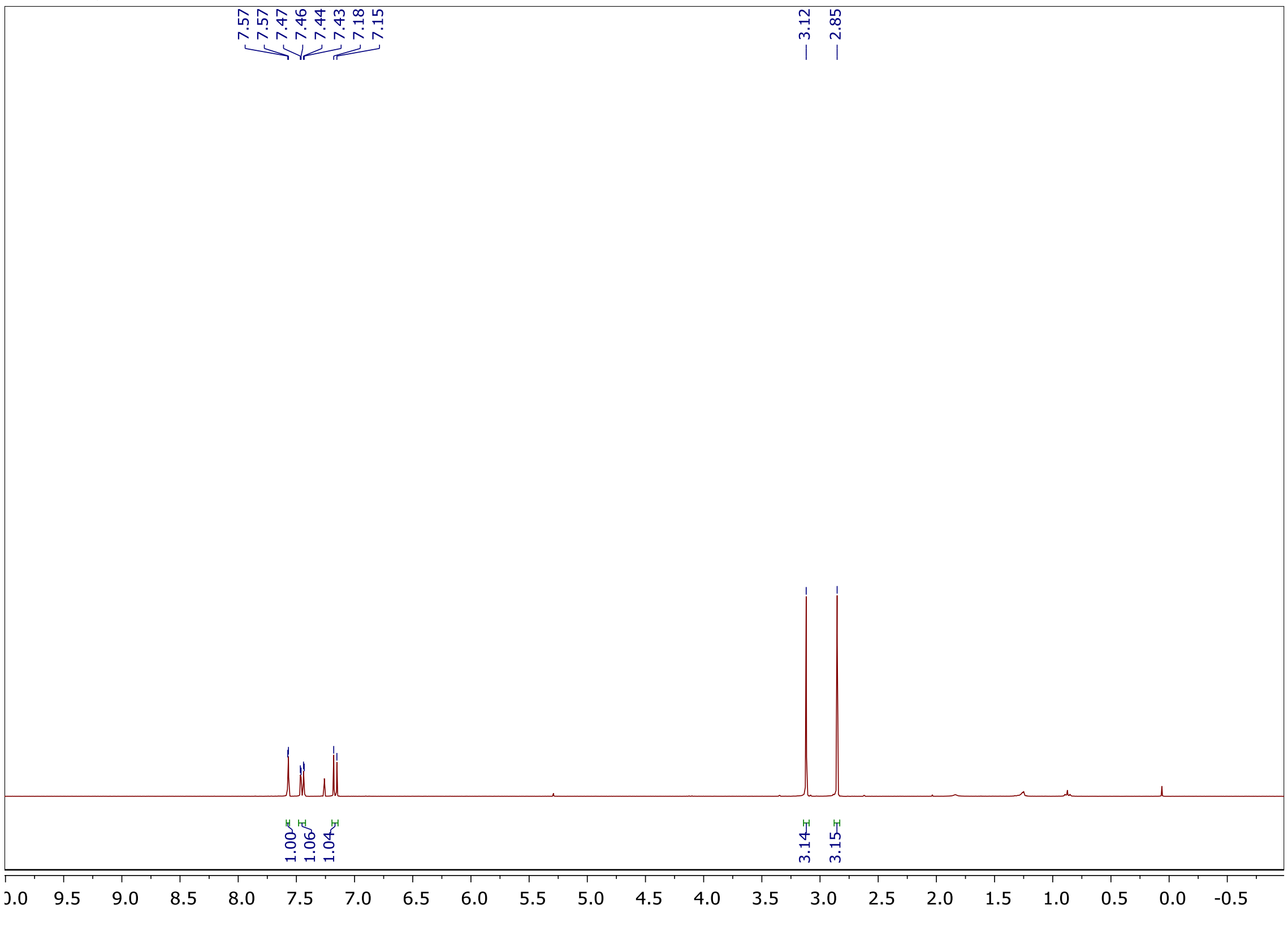

^{13}C NMR (75 MHz, $CDCl_3$)

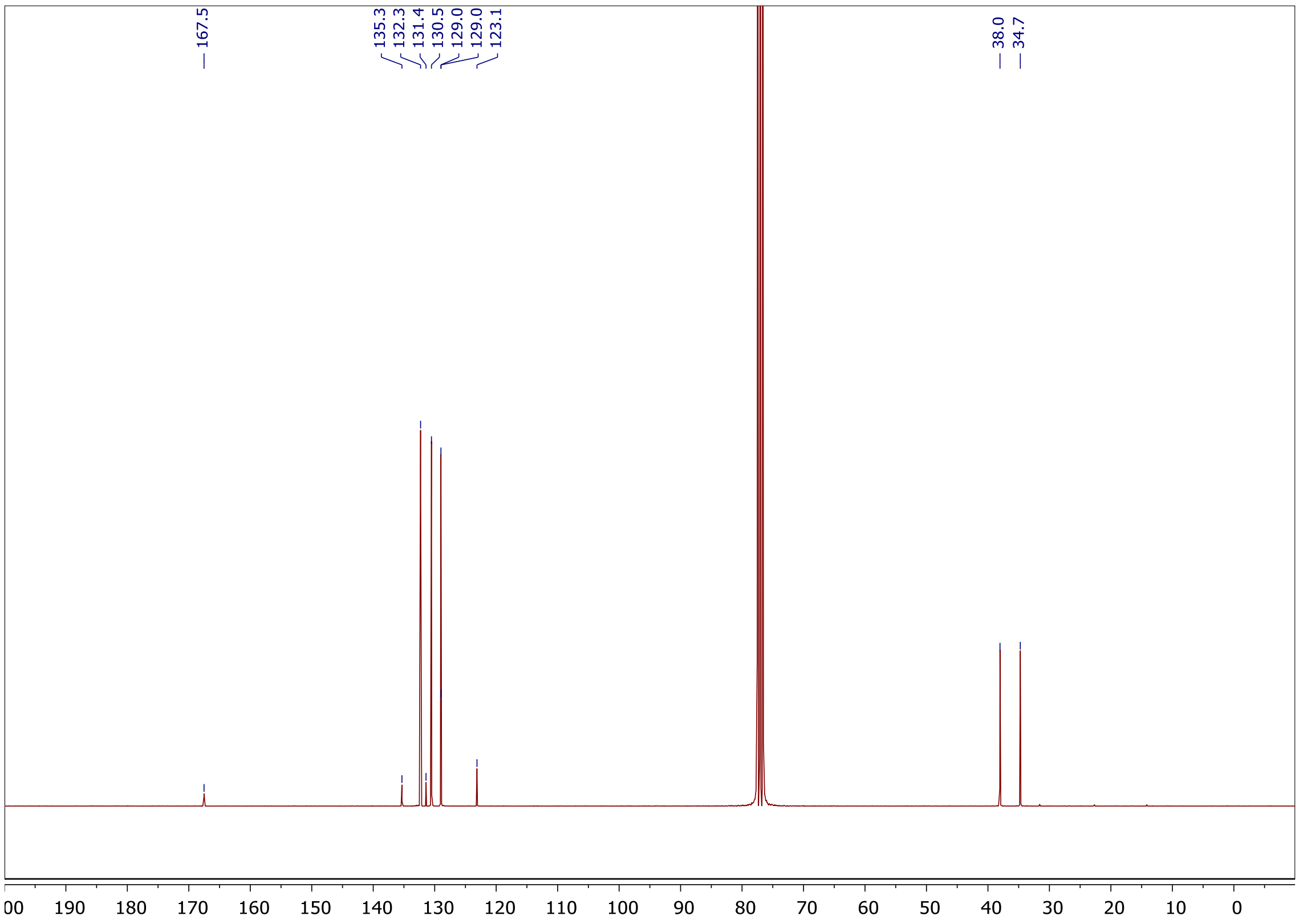

***tert*-Butyl 4-(3'-chloro-4'-[dimethylcarbamoyl]-[1,1'-biphenyl]-4-yl)piperazine-1-carboxylate (14a)**

^1H NMR (300 MHz, $CDCl_3$)

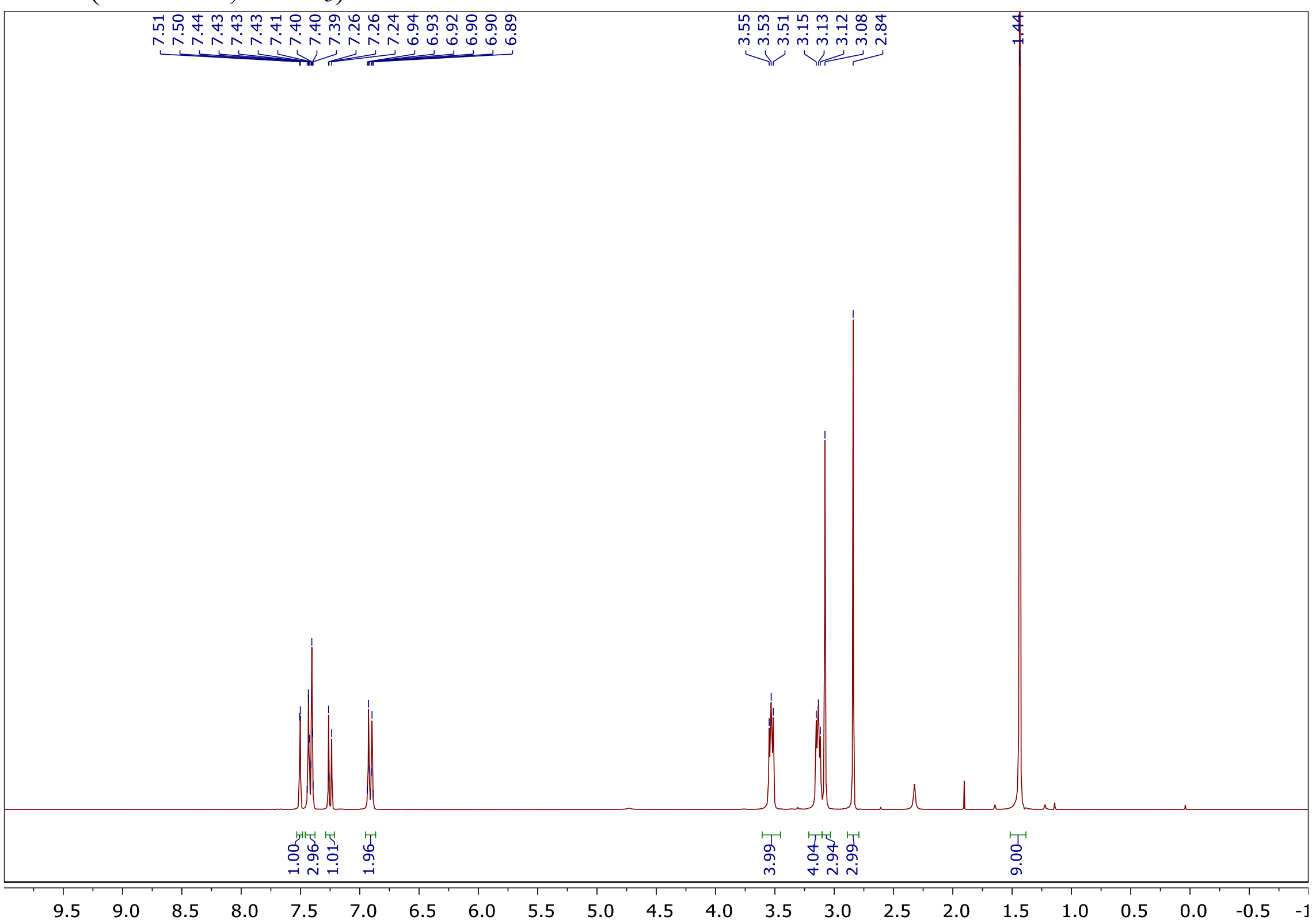

^{13}C NMR (75 MHz, $CDCl_3$)

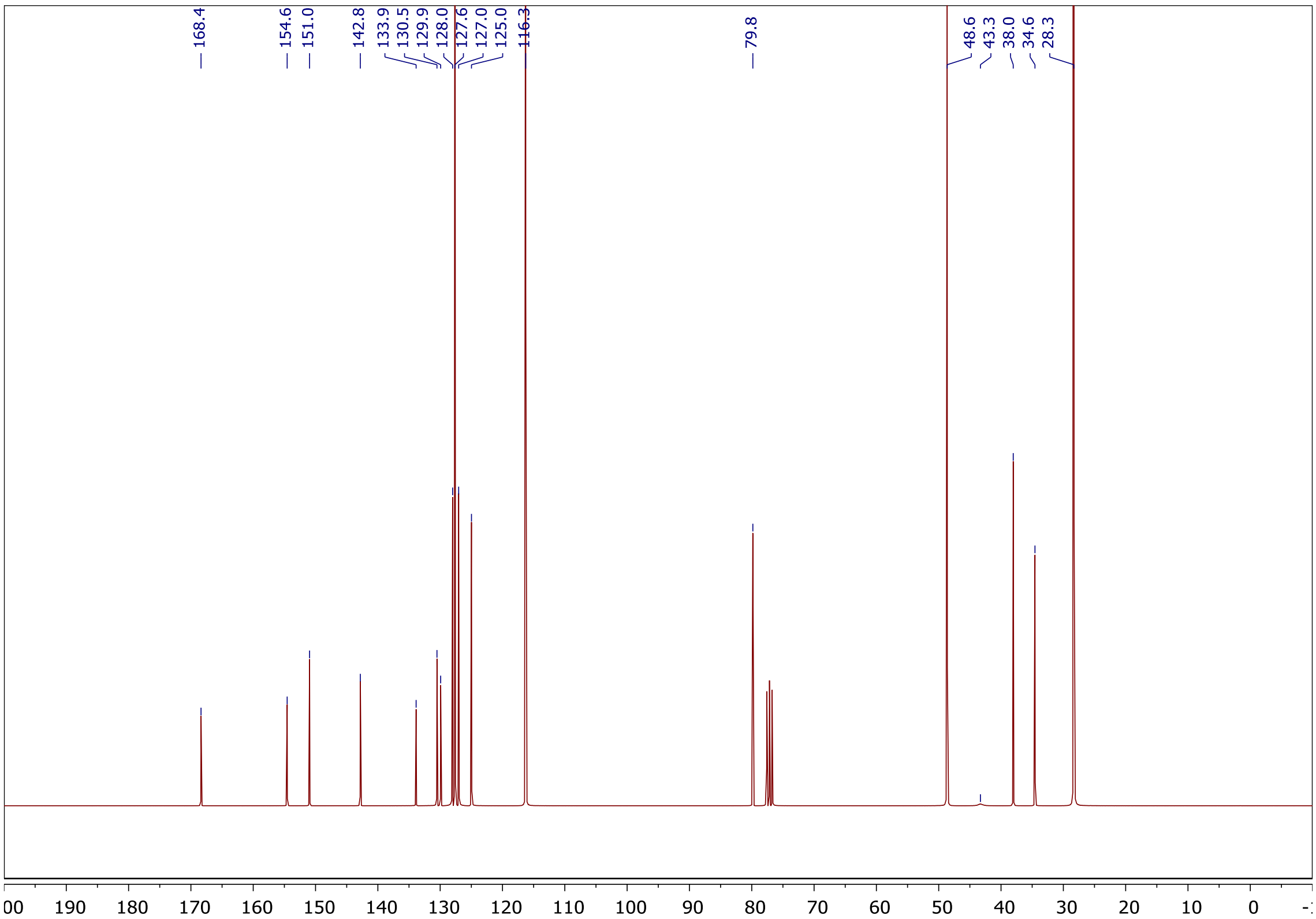

3-Chloro-*N,N*-dimethyl-4'-(4-[3-methyl-2-oxobutanoyl]piperazin-1-yl)-[1,1'-biphenyl]-4-carboxamide (16a)

^{1}H NMR (300 MHz, acetone-d_6)

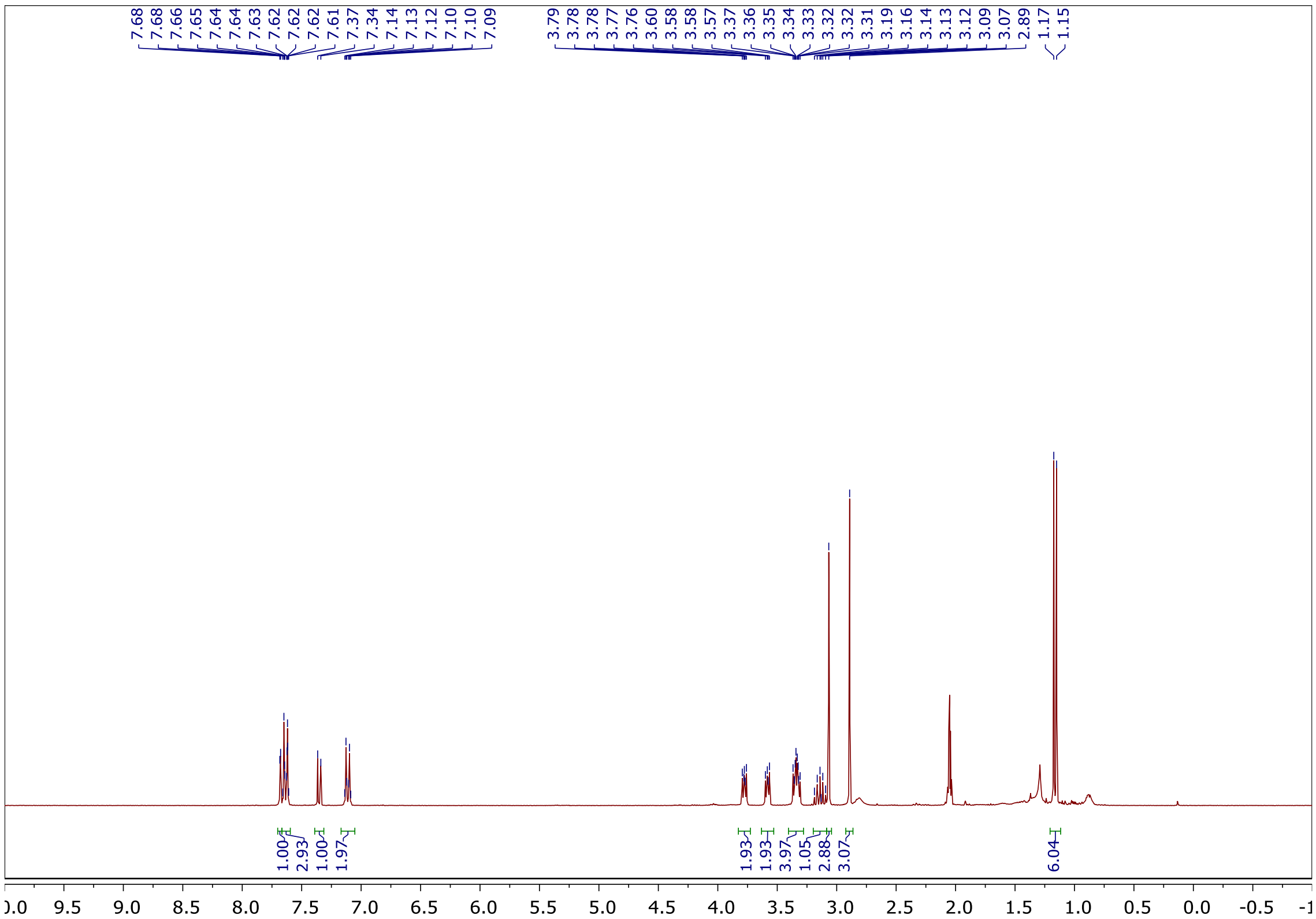

^{13}C NMR (75 MHz, acetone-d_6)

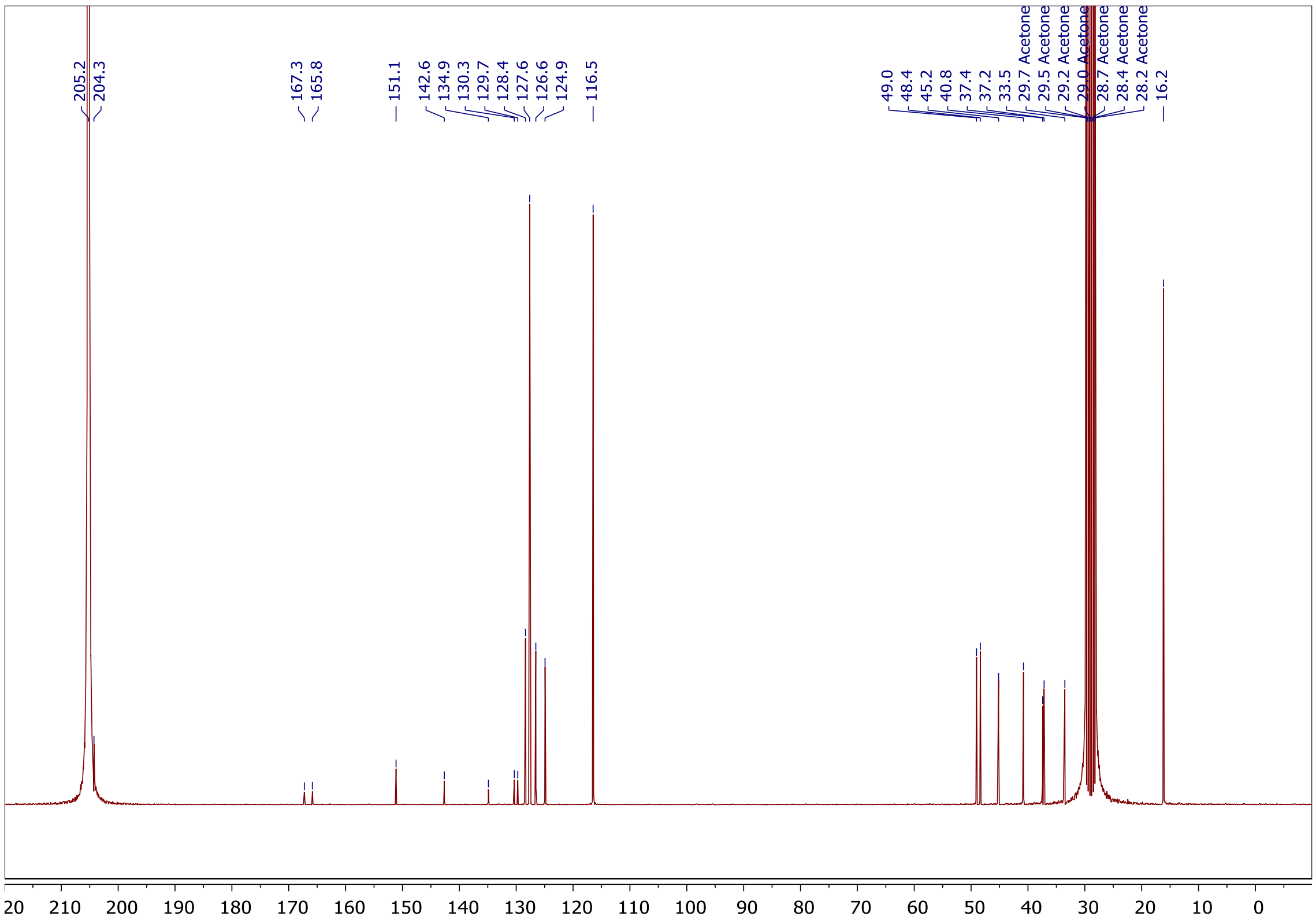

3-Chloro-4'-(4-[2-hydroxy-3-methyl-2-{trifluoromethyl}butanoyl]piperazin-1-yl)-*N*,*N*-dimethyl-[1,1'-biphenyl]-4-carboxamide (*rac*-5)

^{1}H NMR (300 MHz, acetone-d_6)

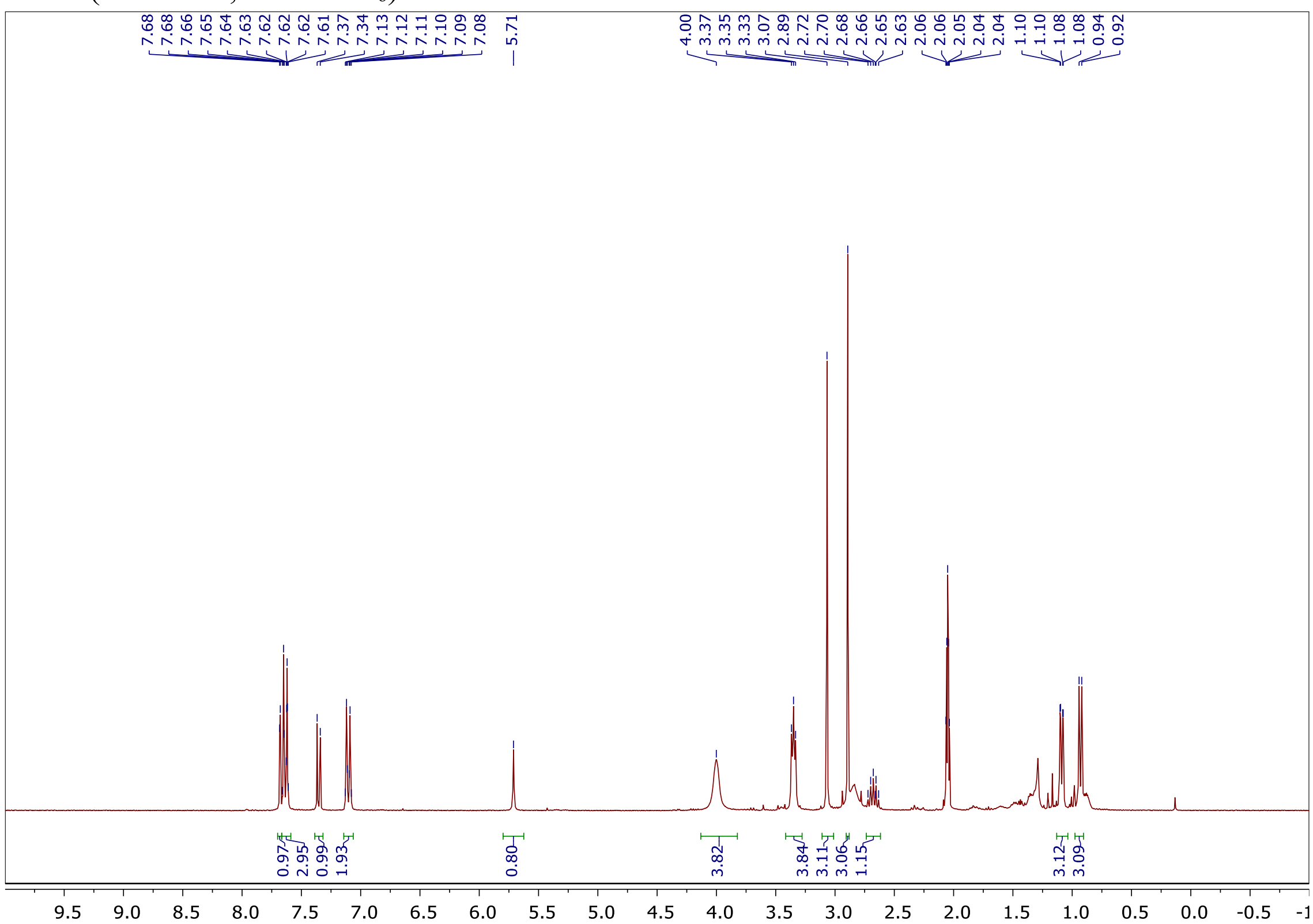

^{13}C NMR (75 MHz, acetone-d_6)

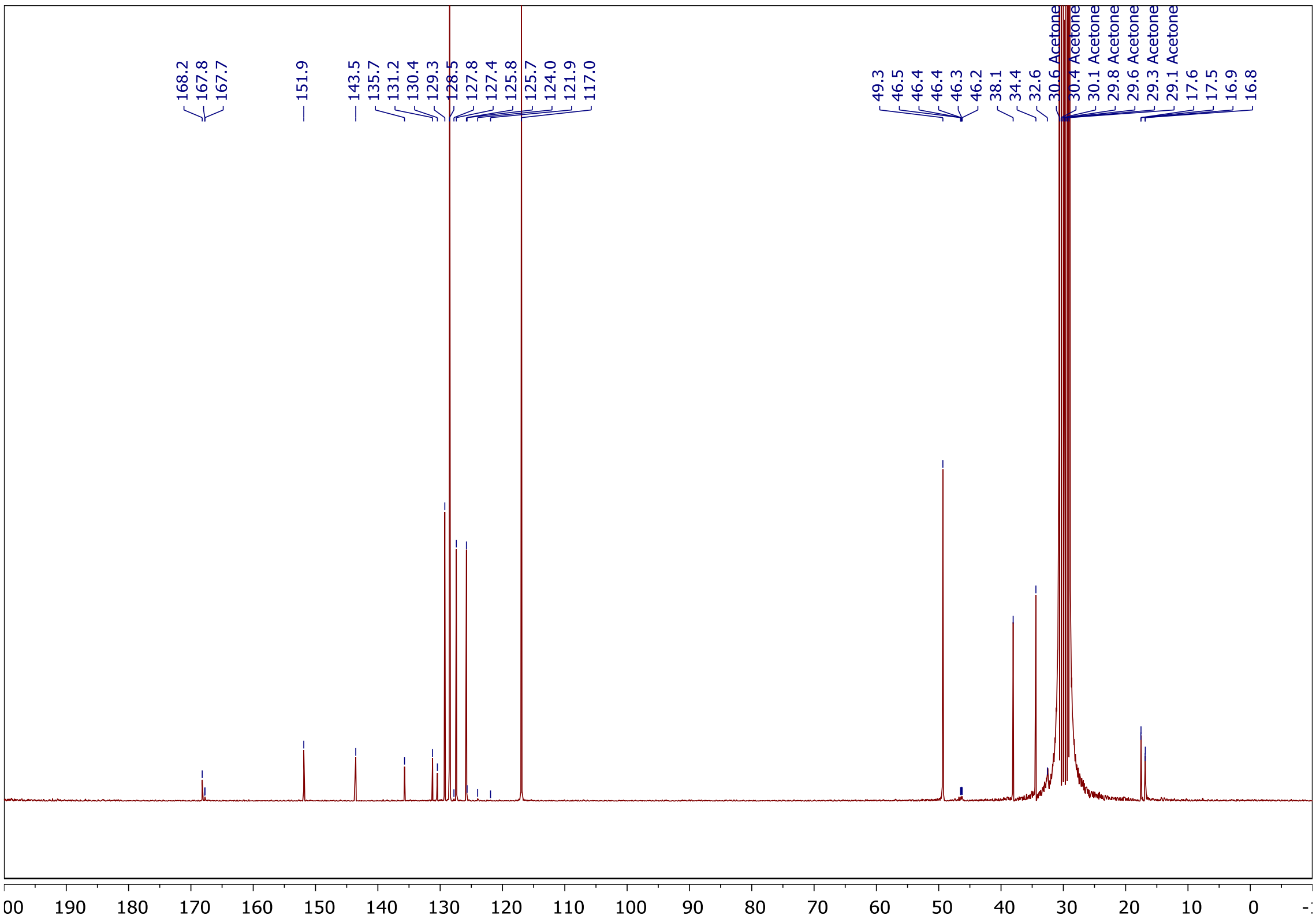

^{19}F NMR (282 MHz, acetone- d_6)

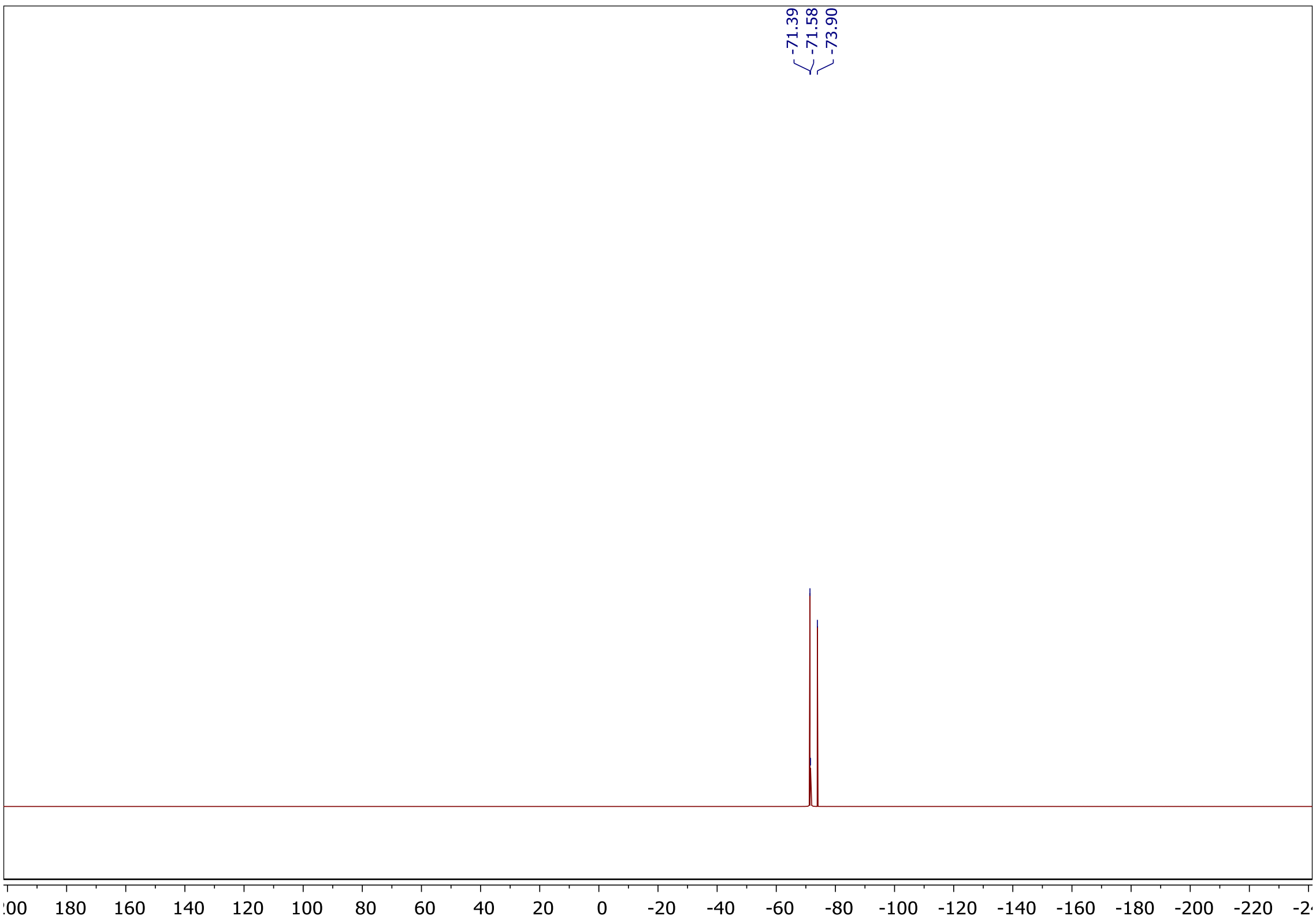

HPLC Traces

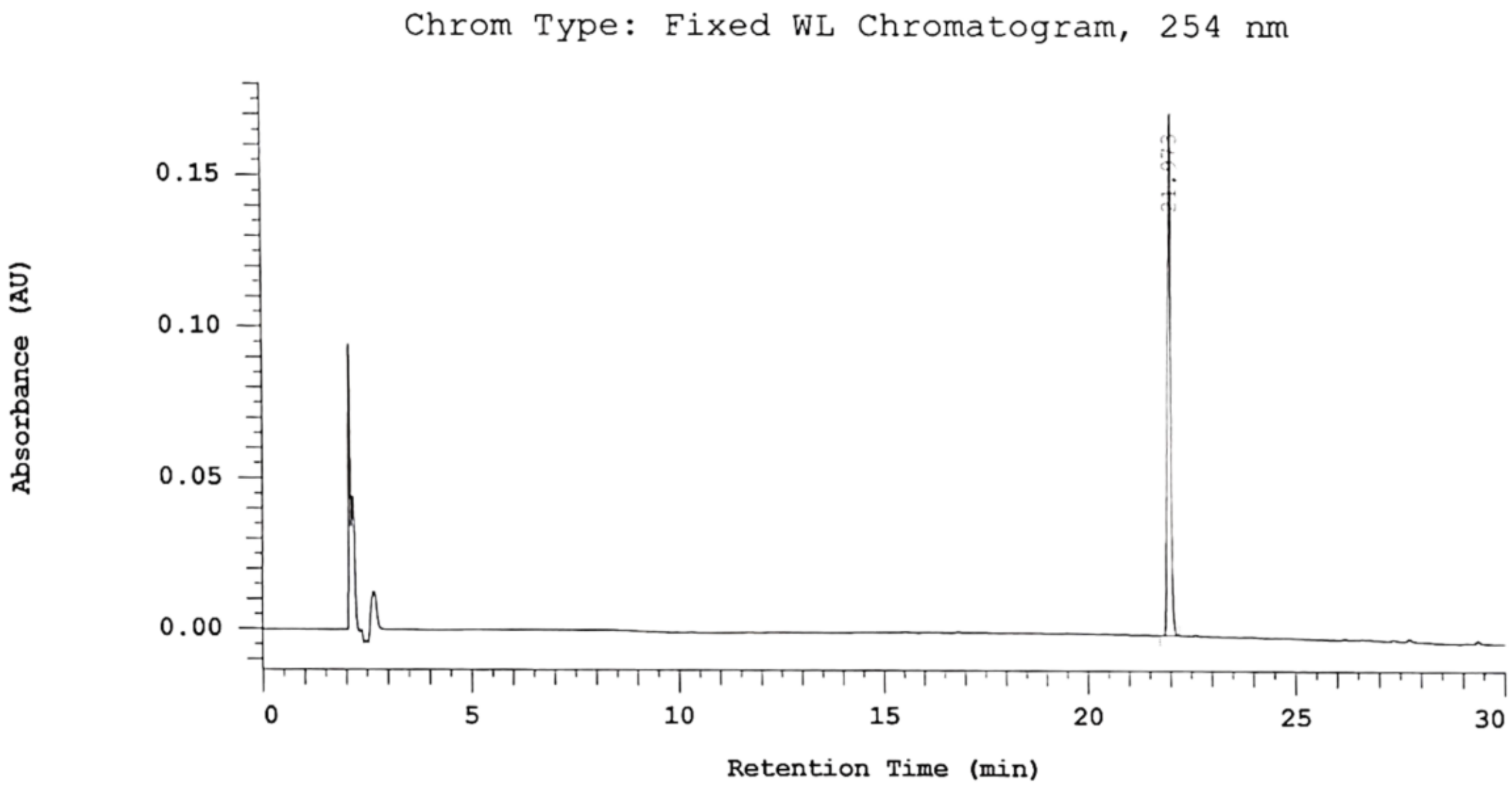

No.	RT	Area	Area %	Height
1	21.973	454692	100.000	86413
		454692	100.000	86413

Peak rejection level: 0

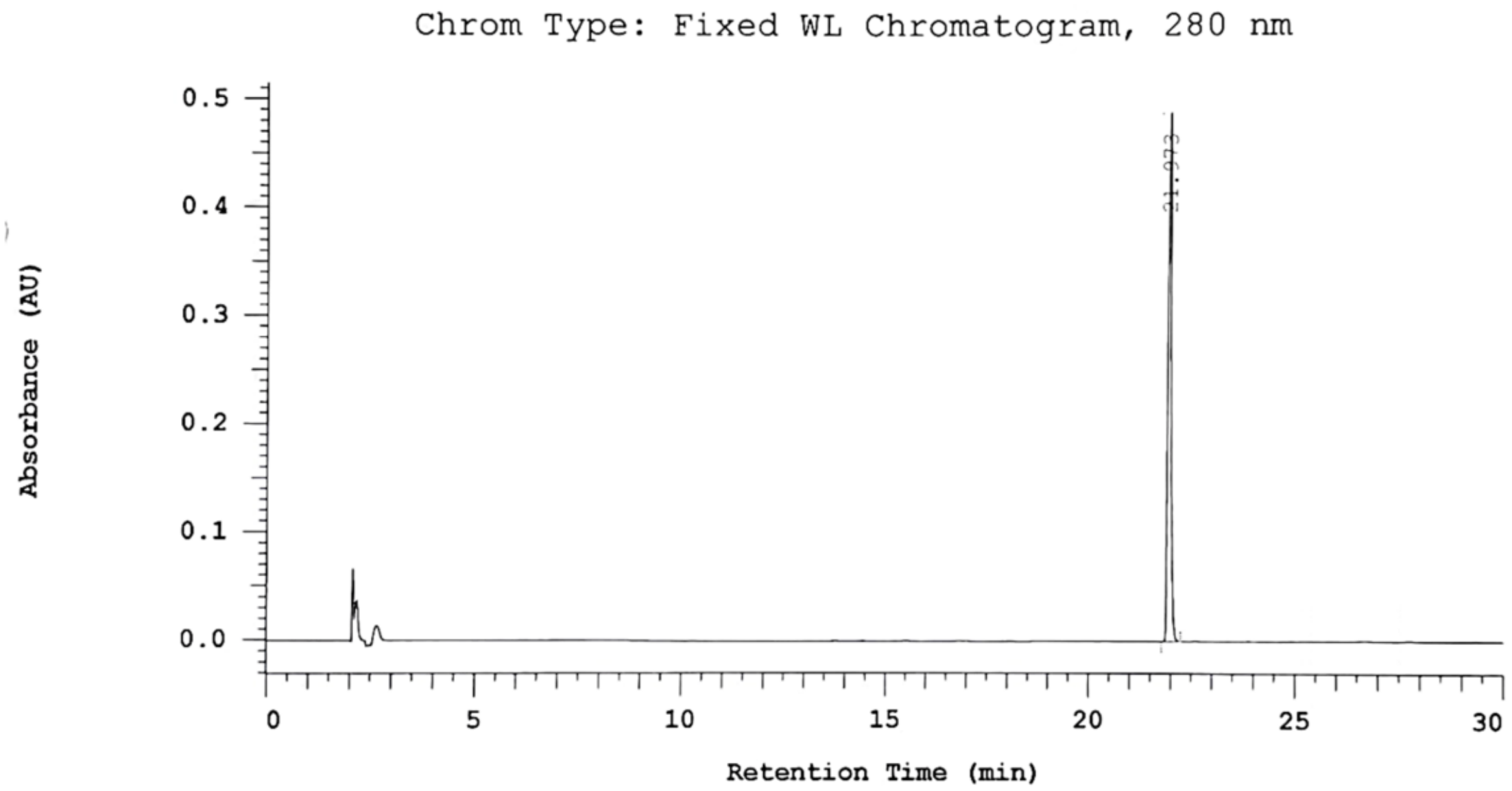

No.	RT	Area	Area %	Height
1	21.973	1294422	100.000	244934
		1294422	100.000	244934

Peak rejection level: 0

2-Chloro-*N*,*N*-dimethyl-4-(4,4,5,5-tetramethyl-1,3,2-dioxaborolan-2-yl)benzamide (12)

^{1}H NMR (400 MHz, $CDCl_3$)

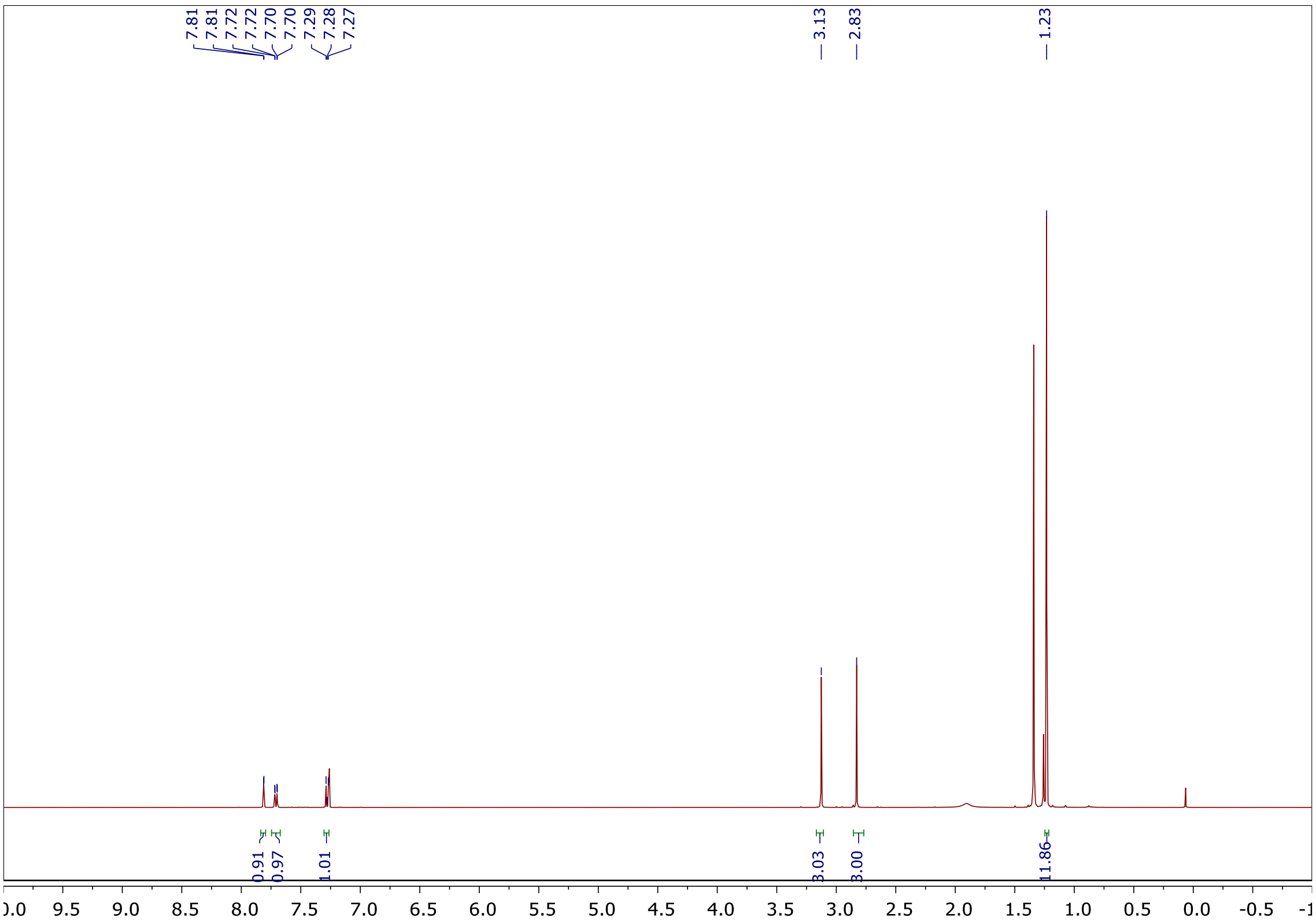

^{13}C NMR (101 MHz, $CDCl_3$)

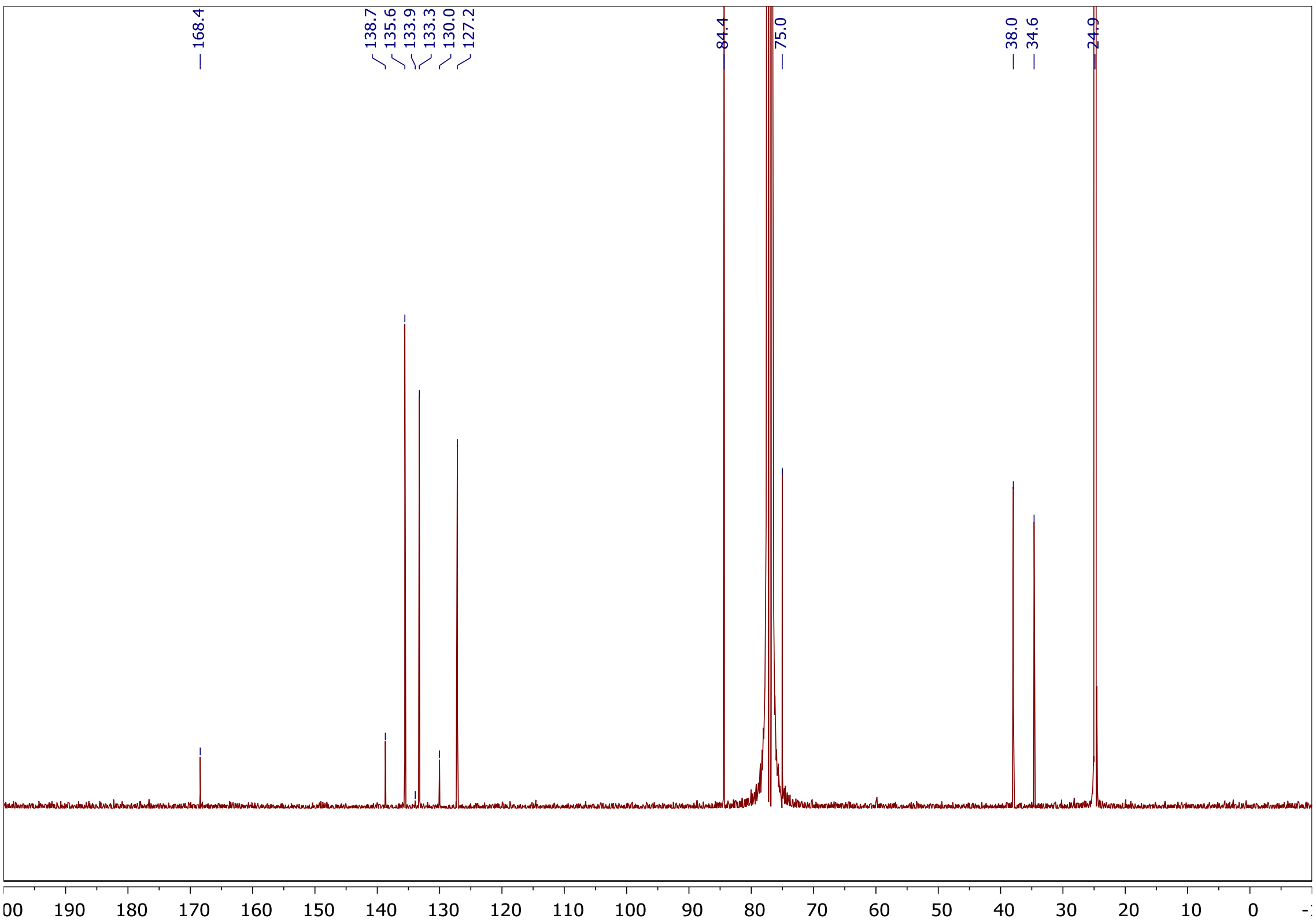

^{11}B NMR (128 MHz, $CDCl_3$)

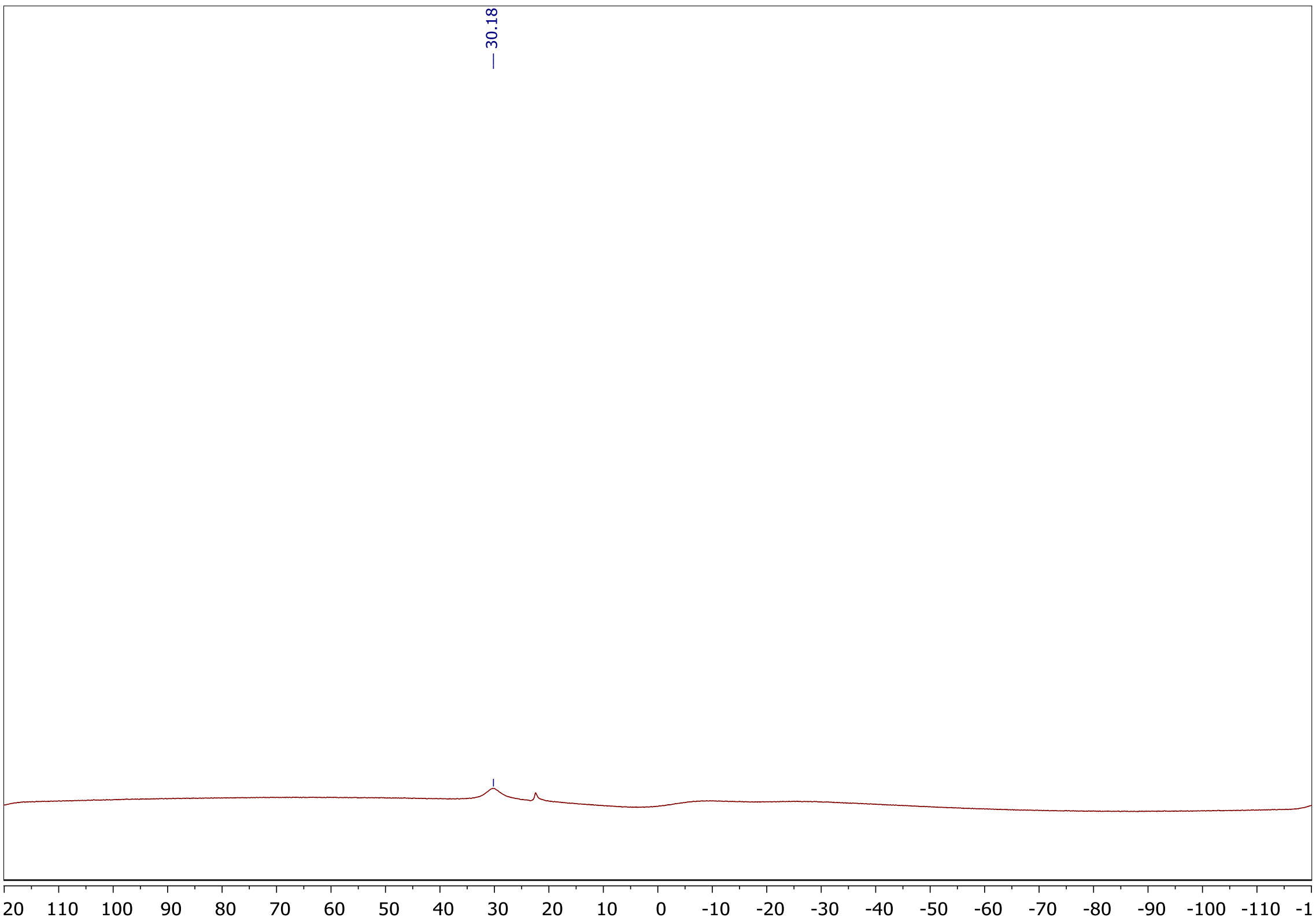

***tert*-Butyl (3a*R*,6a*S*)-5-(5-bromopyridin-2-yl)hexahydropyrrolo[3,4-*c*]pyrrole-2(1*H*)-carboxylate (13b)**

^{1}H NMR (300 MHz, $CDCl_3$)

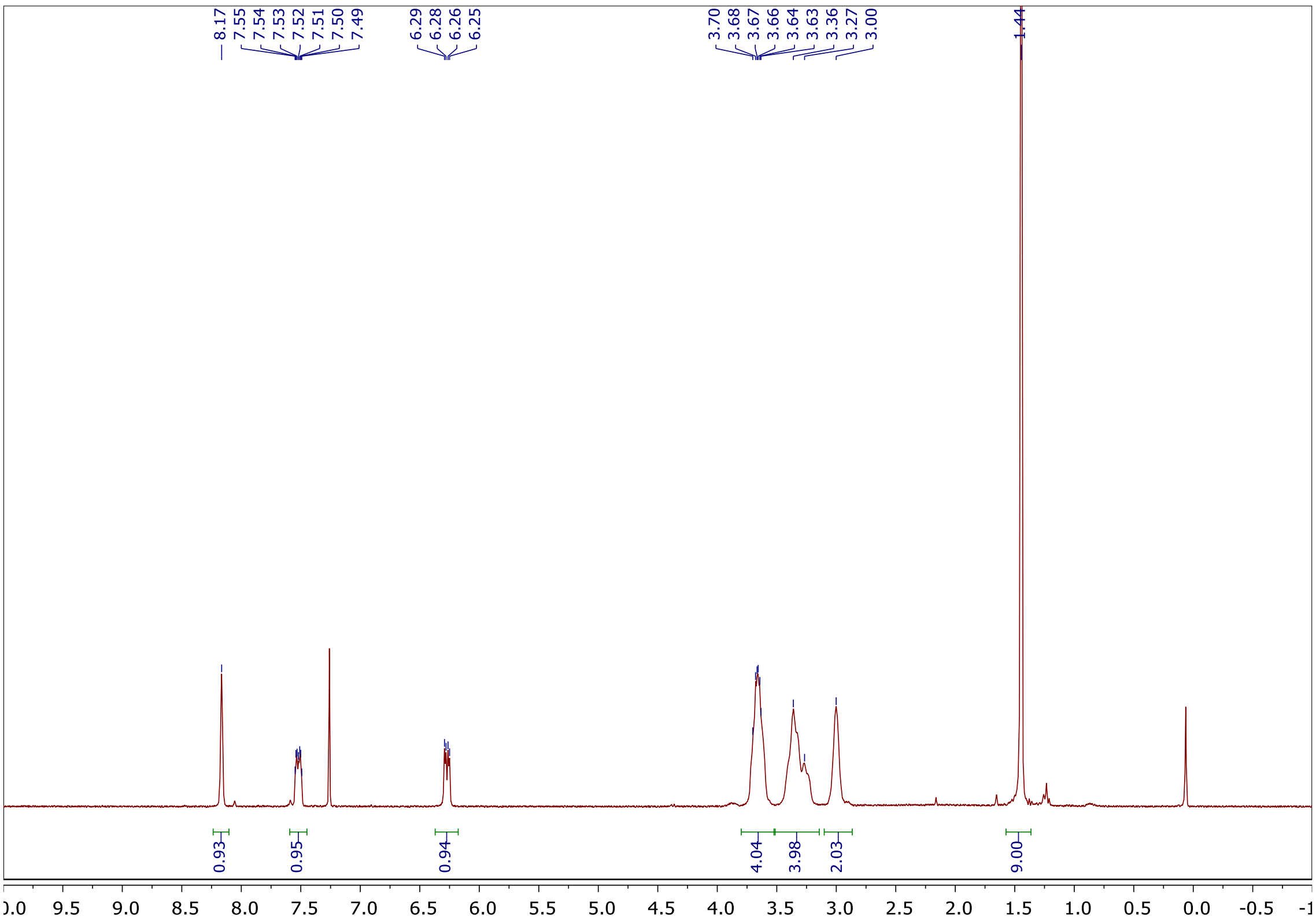

^{13}C NMR (75 MHz, $CDCl_3$)

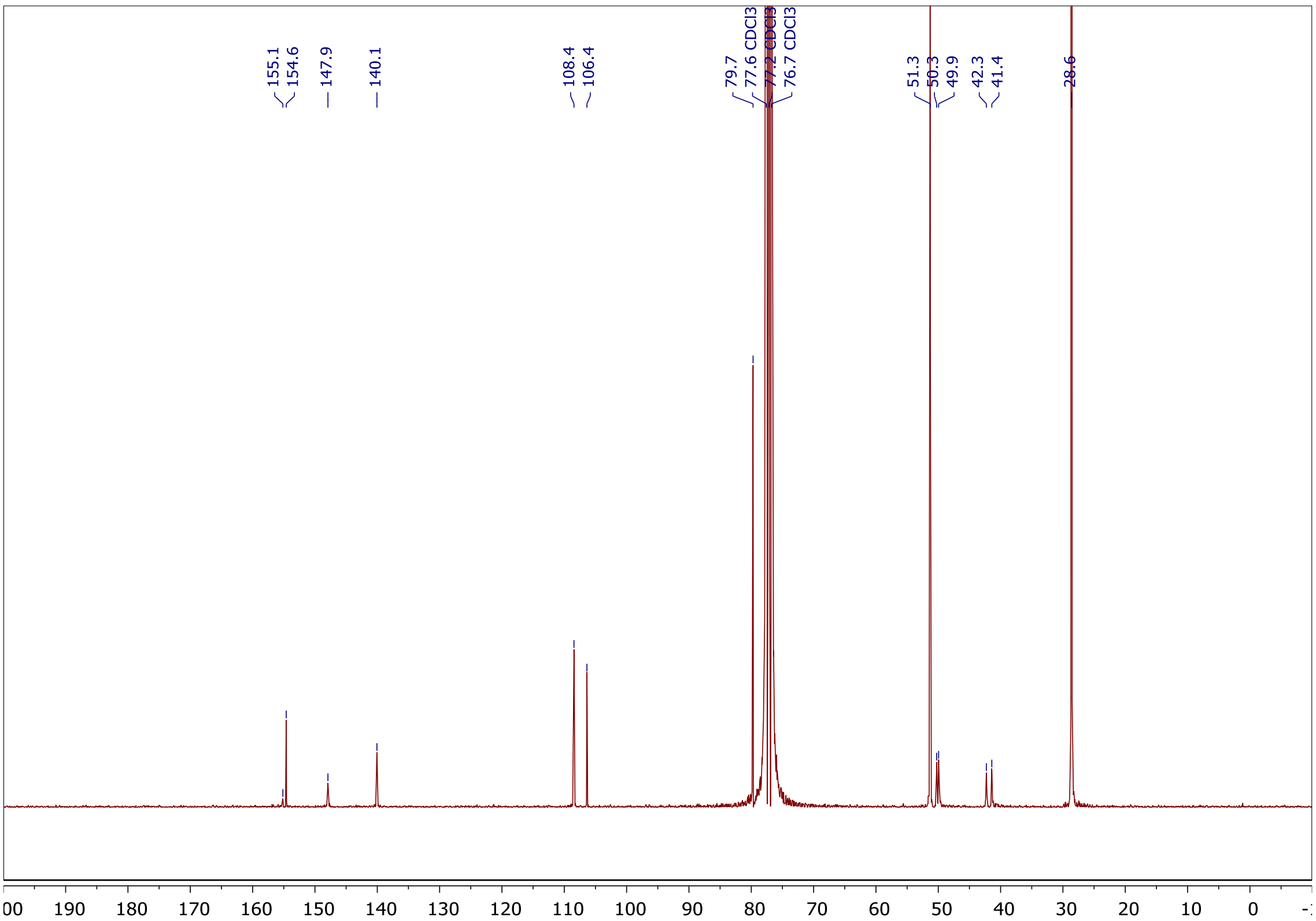

***tert*-Butyl (3a*R*,6a*S*)-5-(5-[3-chloro-4-{dimethylcarbamoyl}phenyl]pyridin-2-yl)hexahydropyrrolo[3,4-*c*]pyrrole-2(1*H*)-carboxylate (14b)**

^{1}H NMR (400 MHz, $CDCl_3$)

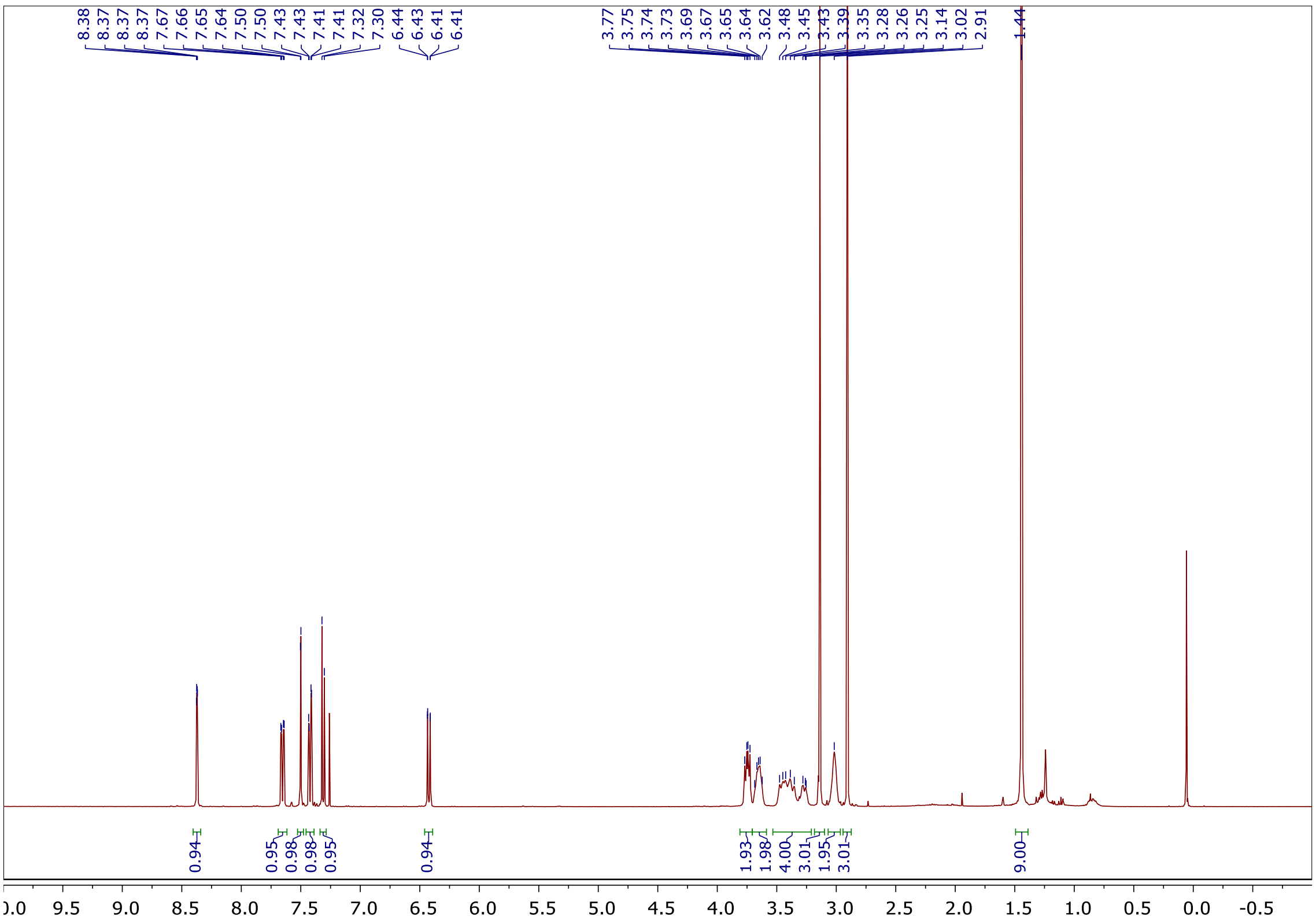

^{13}C NMR (101 MHz, $CDCl_3$)

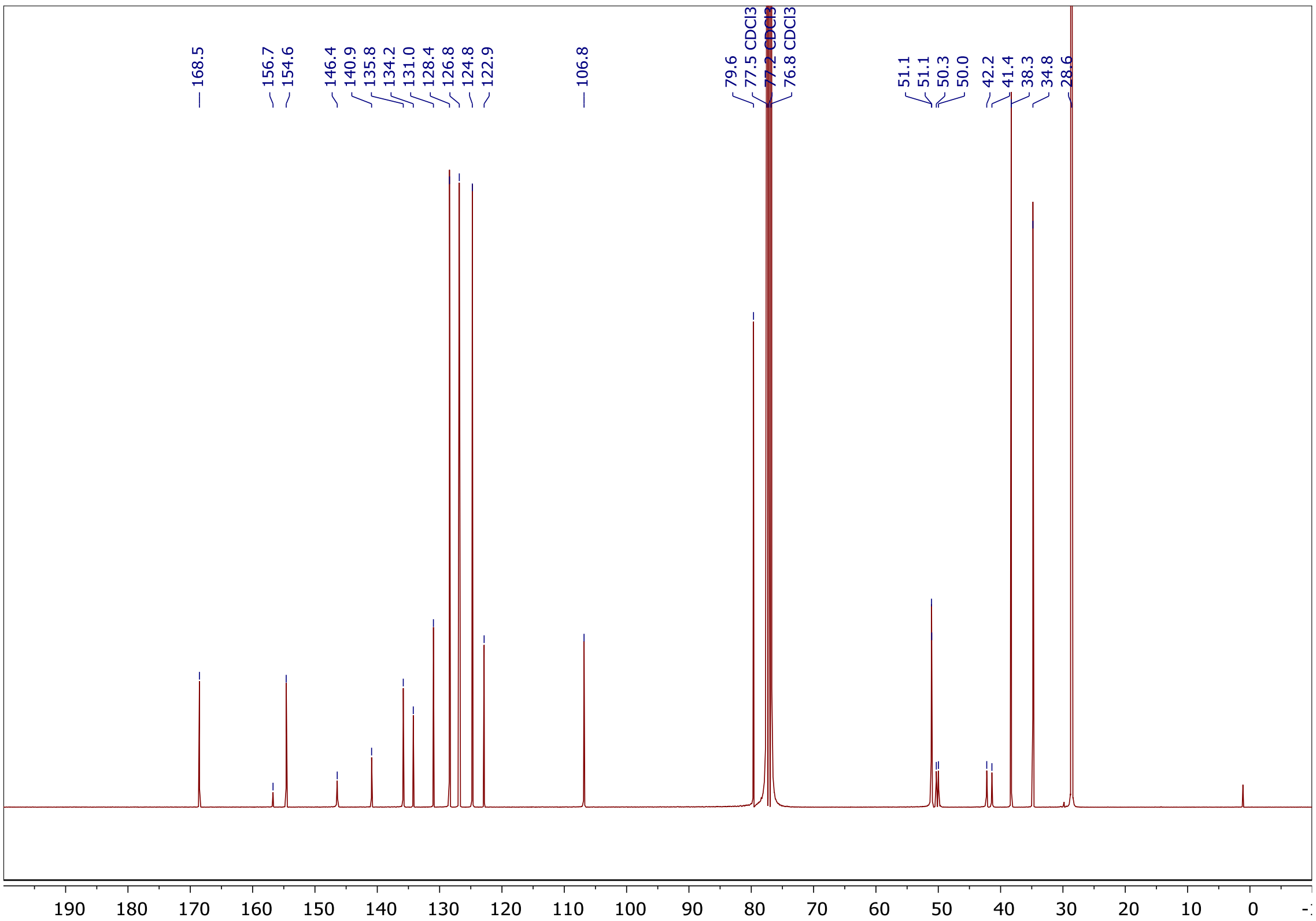

2-Chloro-*N*,*N*-dimethyl-4-(6-[{3a*R*,6a*S*}-5-{3-methyl-2-oxobutanoyl}hexahydropyrrolo[3,4-*c*]pyrrol-2{1*H*}-yl]pyridin-3-yl)benzamide (16b)

^{1}H NMR (300 MHz, acetone-d_6)

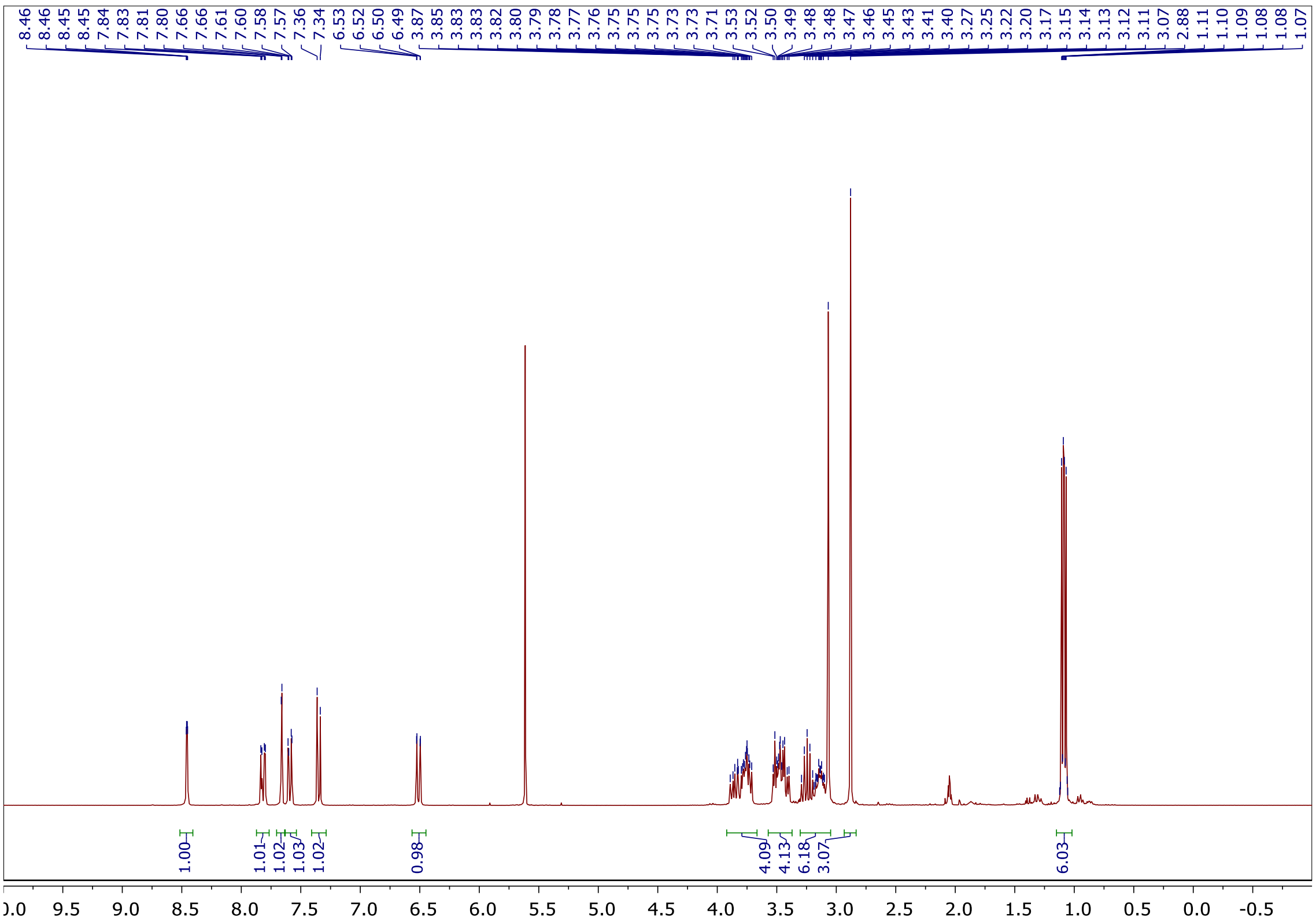

^{13}C NMR (75 MHz, acetone-d_6)

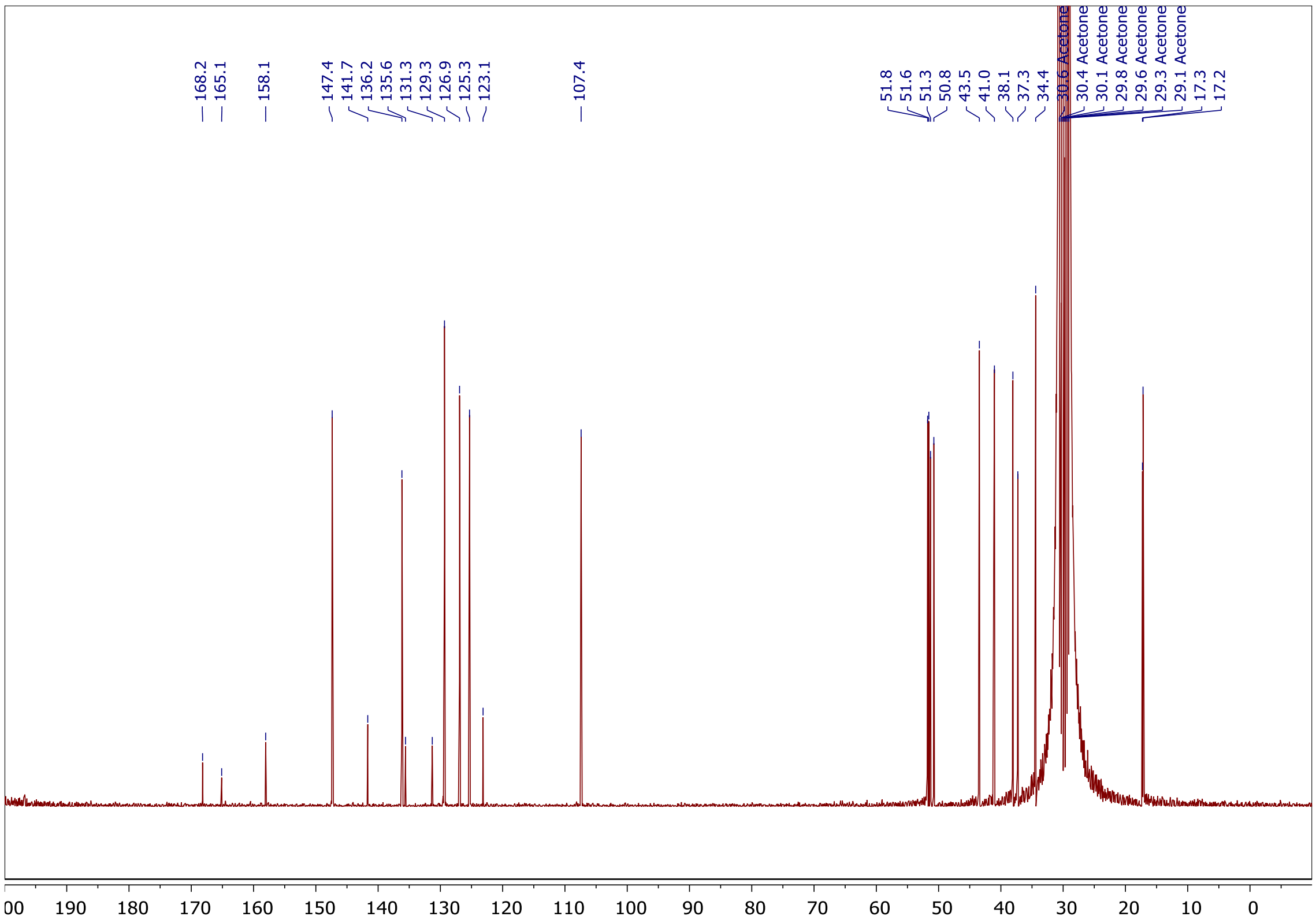

2-Chloro-4-(6-[{3a*R*,6a*S*}-5-{2-hydroxy-3-methyl-2-(trifluoromethyl)butanoyl}hexahydropyrrolo[3,4-*c*]pyrrol-2{1*H*}-yl]pyridin-3-yl)-*N*,*N*-dimethylbenzamide (*rac*-6)

^{1}H NMR (300 MHz, acetone-d_6)

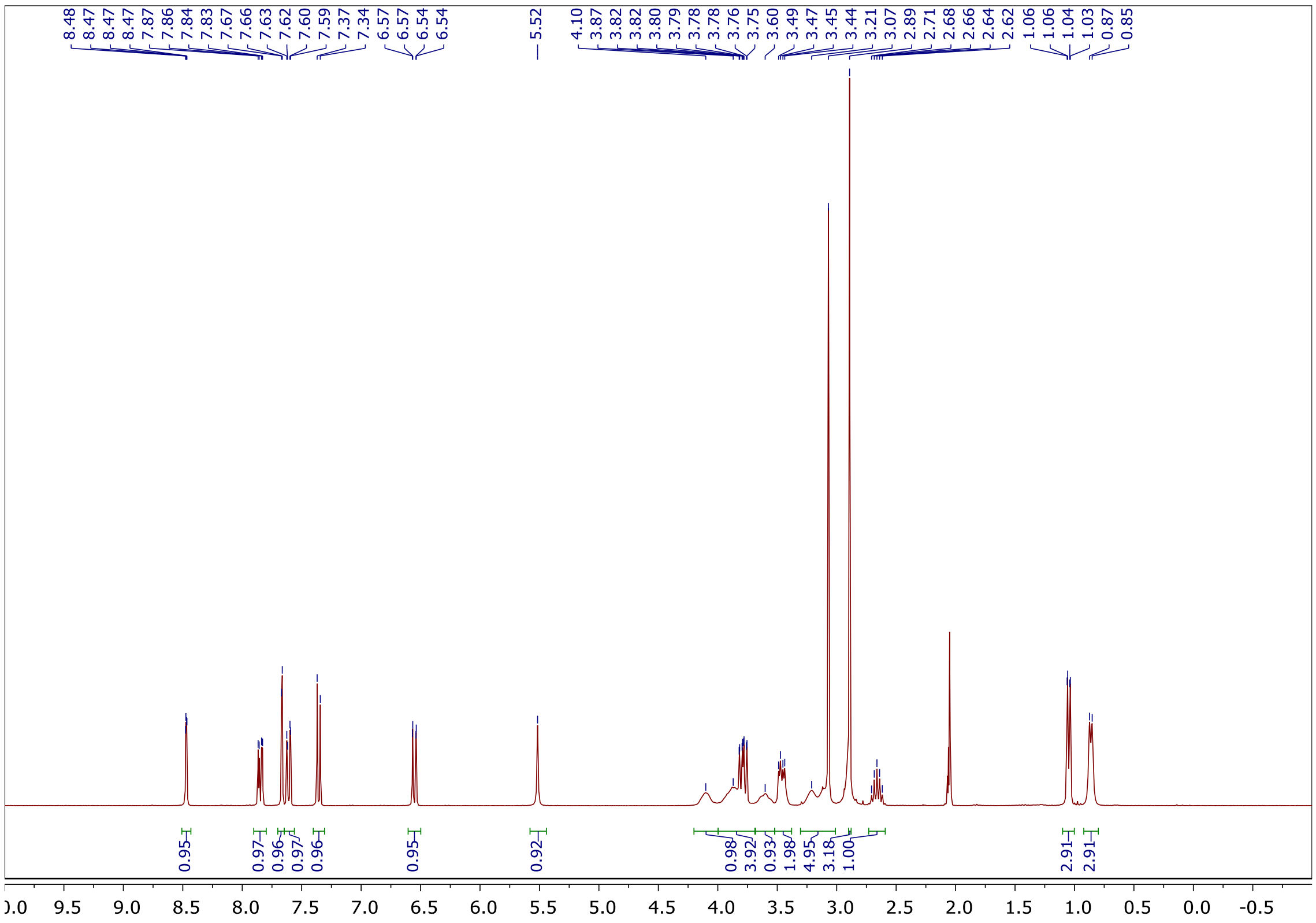

^{13}C NMR (75 MHz, acetone-d_6)

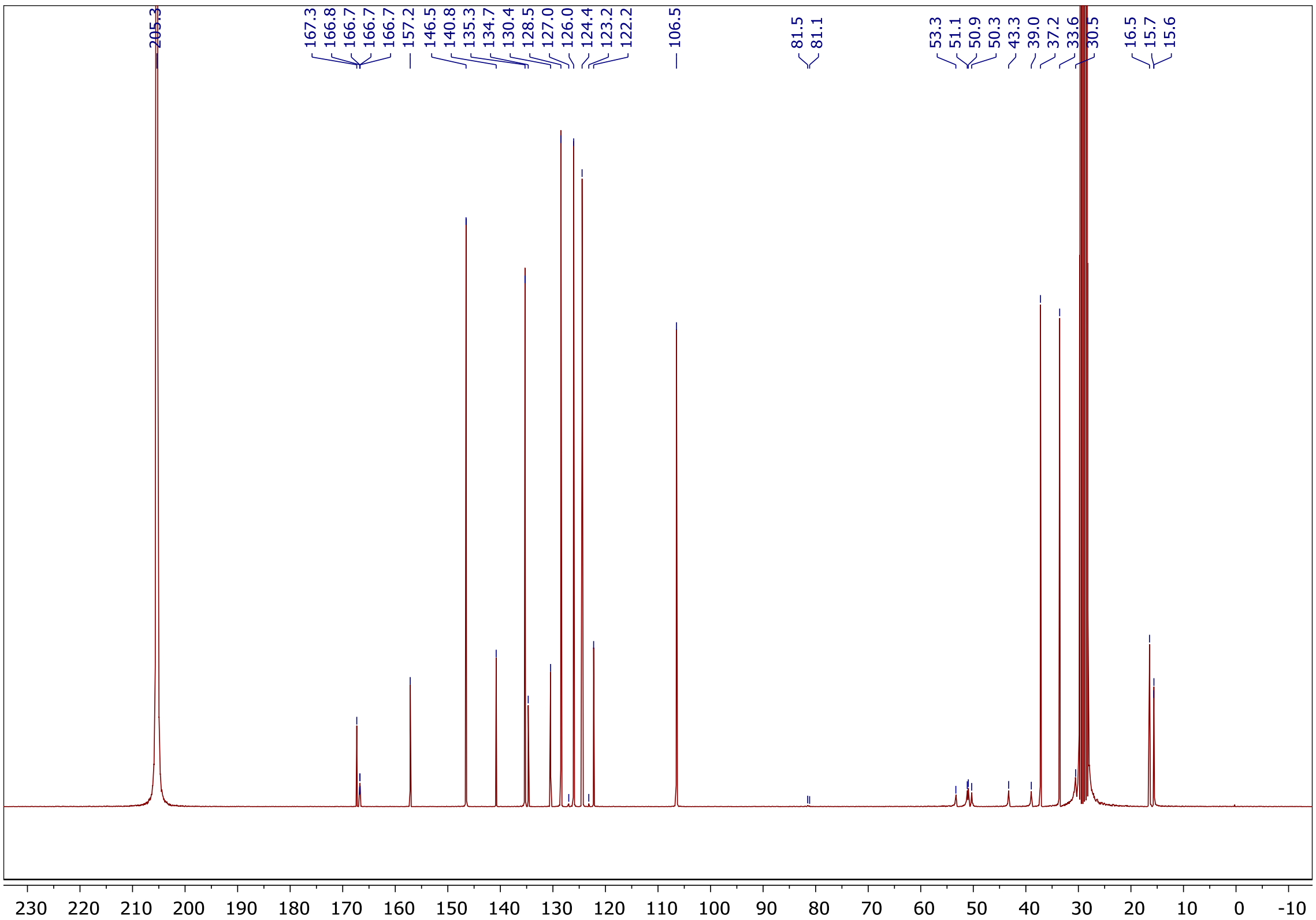

^{19}F NMR (282 MHz, acetone- d_6)

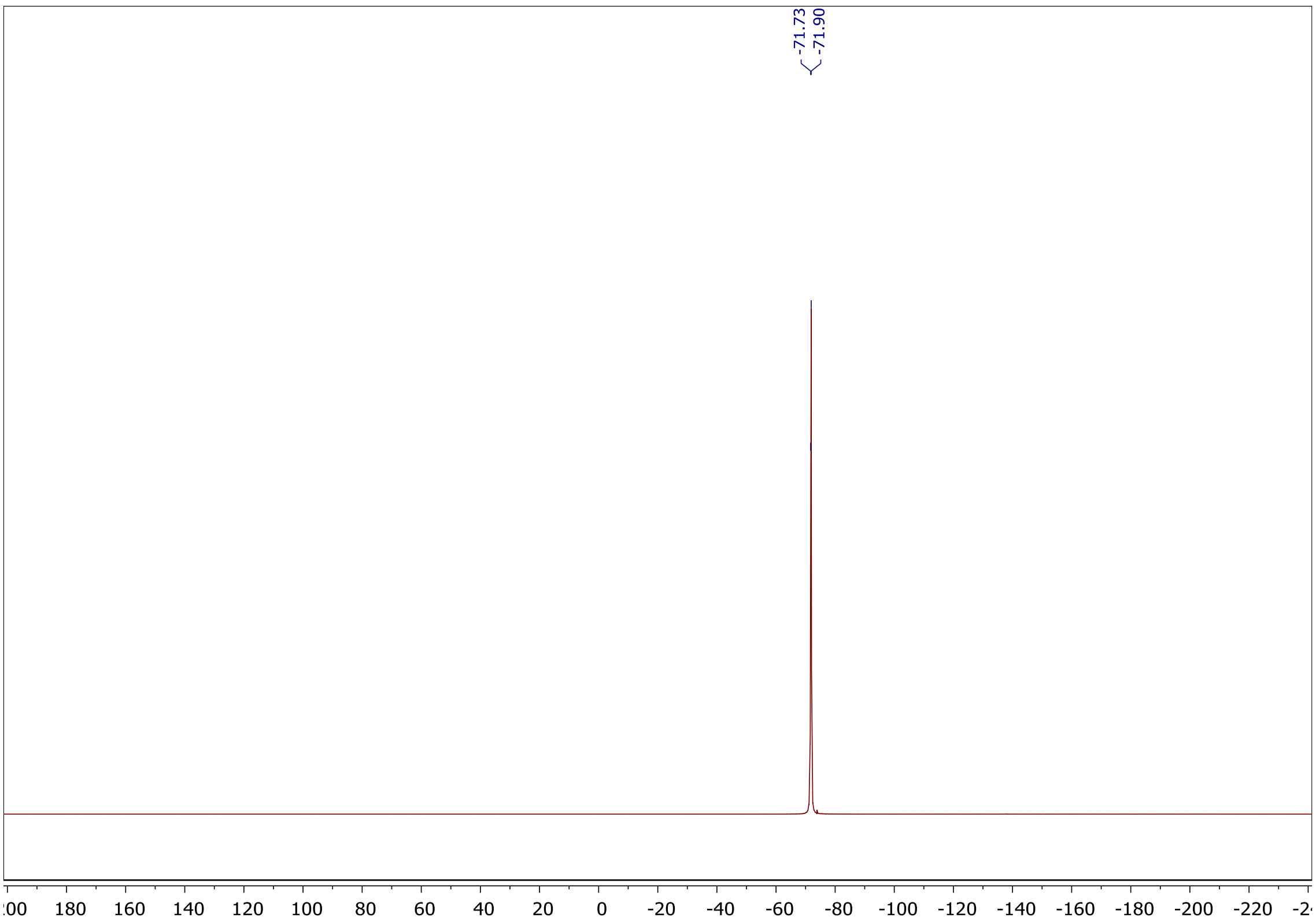

HPLC Traces

Chrom Type: Fixed WL Chromatogram, 254 nm

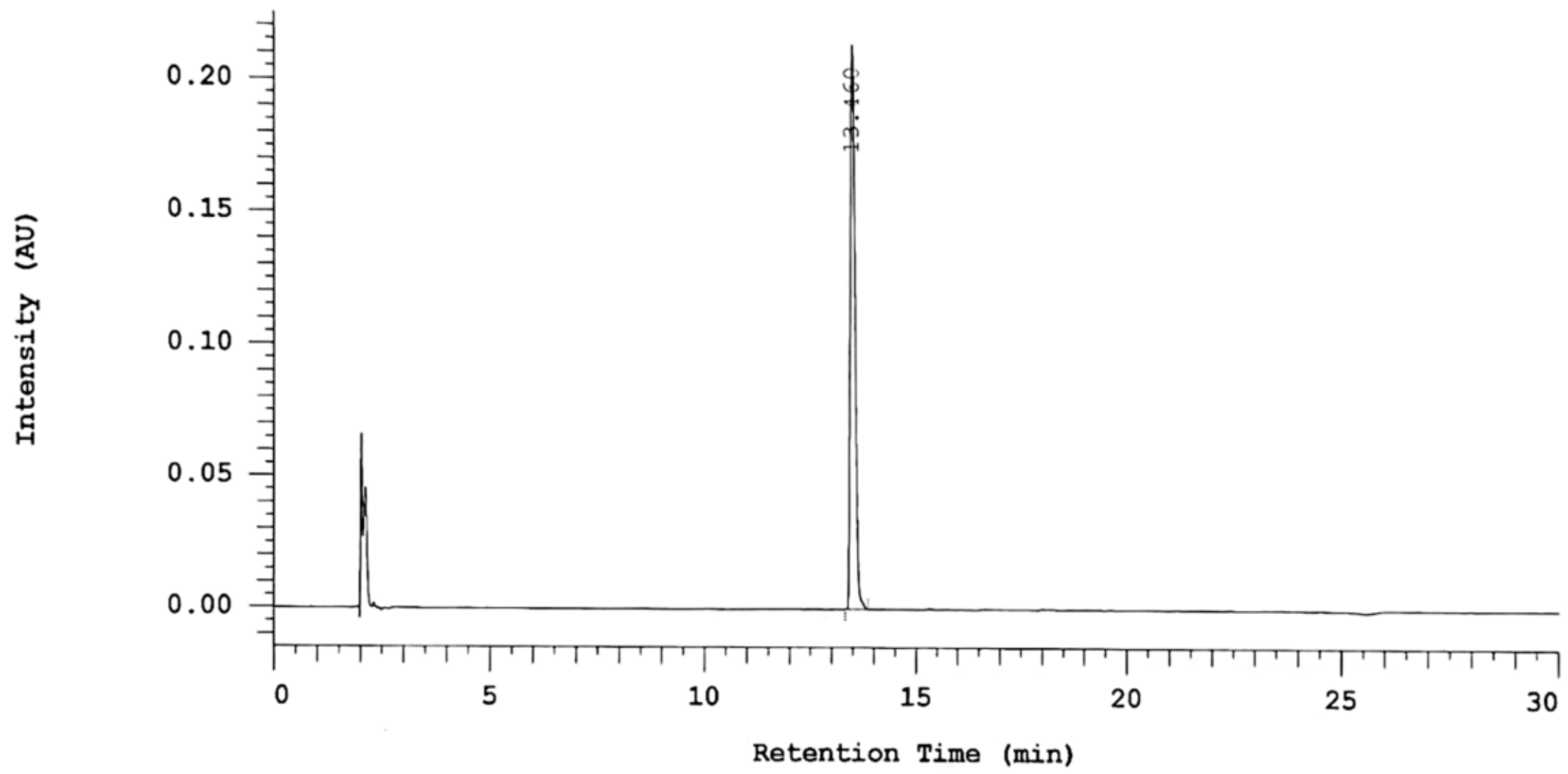

No.	RT	Area	Area %	Height
1	13.460	820863	100.000	106851
		820863	100.000	106851

Peak rejection level: 0

Chrom Type: Fixed WL Chromatogram, 280 nm

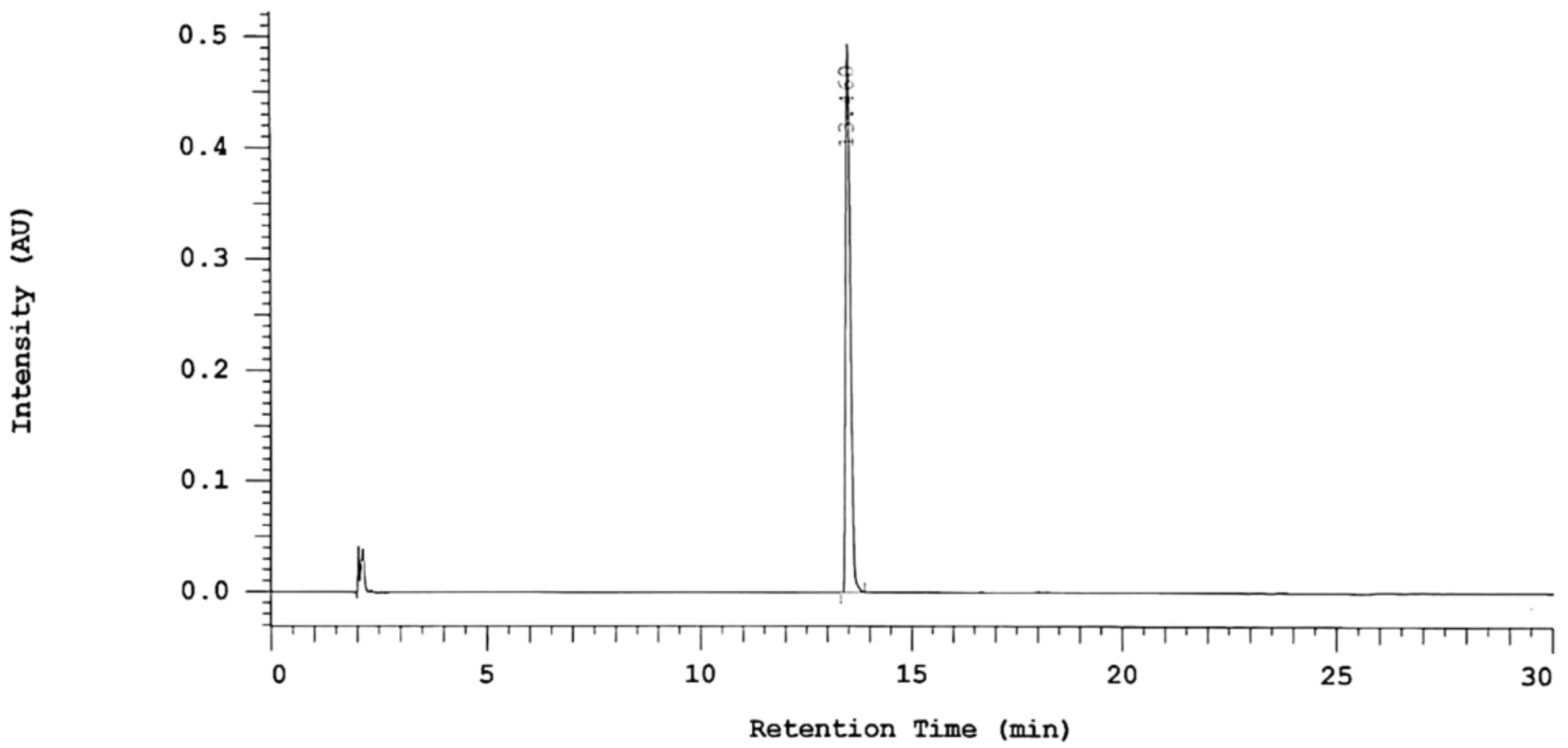

No.	RT	Area	Area %	Height
1	13.460	1910775	100.000	248504
		1910775	100.000	248504

Peak rejection level: 0

([5-Bromo-1*H*-inden-3-yl]oxy)(*tert*-butyl)dimethylsilane (21)

^{1}H NMR (300 MHz, $CDCl_3$)

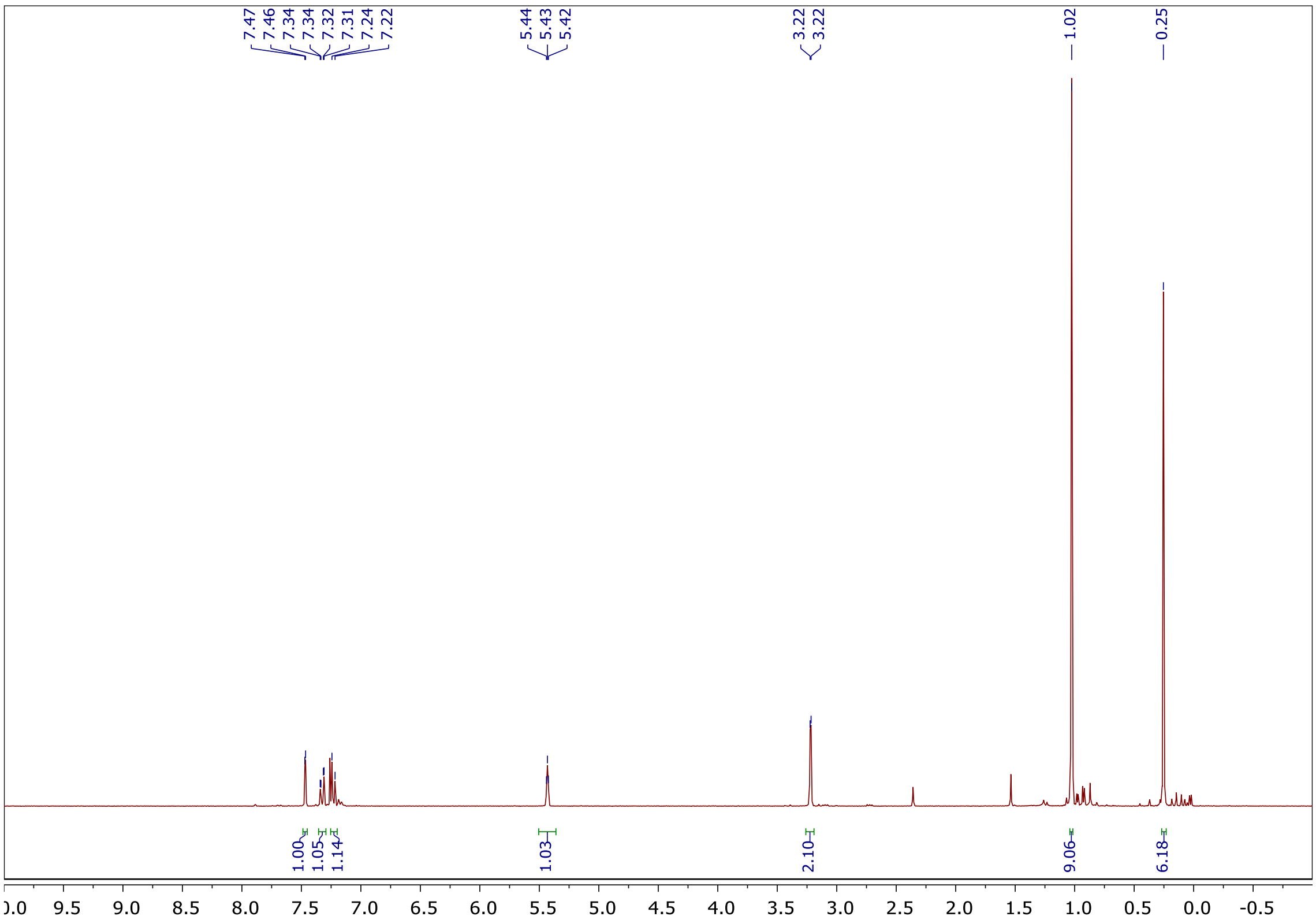

^{13}C NMR (75 MHz, $CDCl_3$)

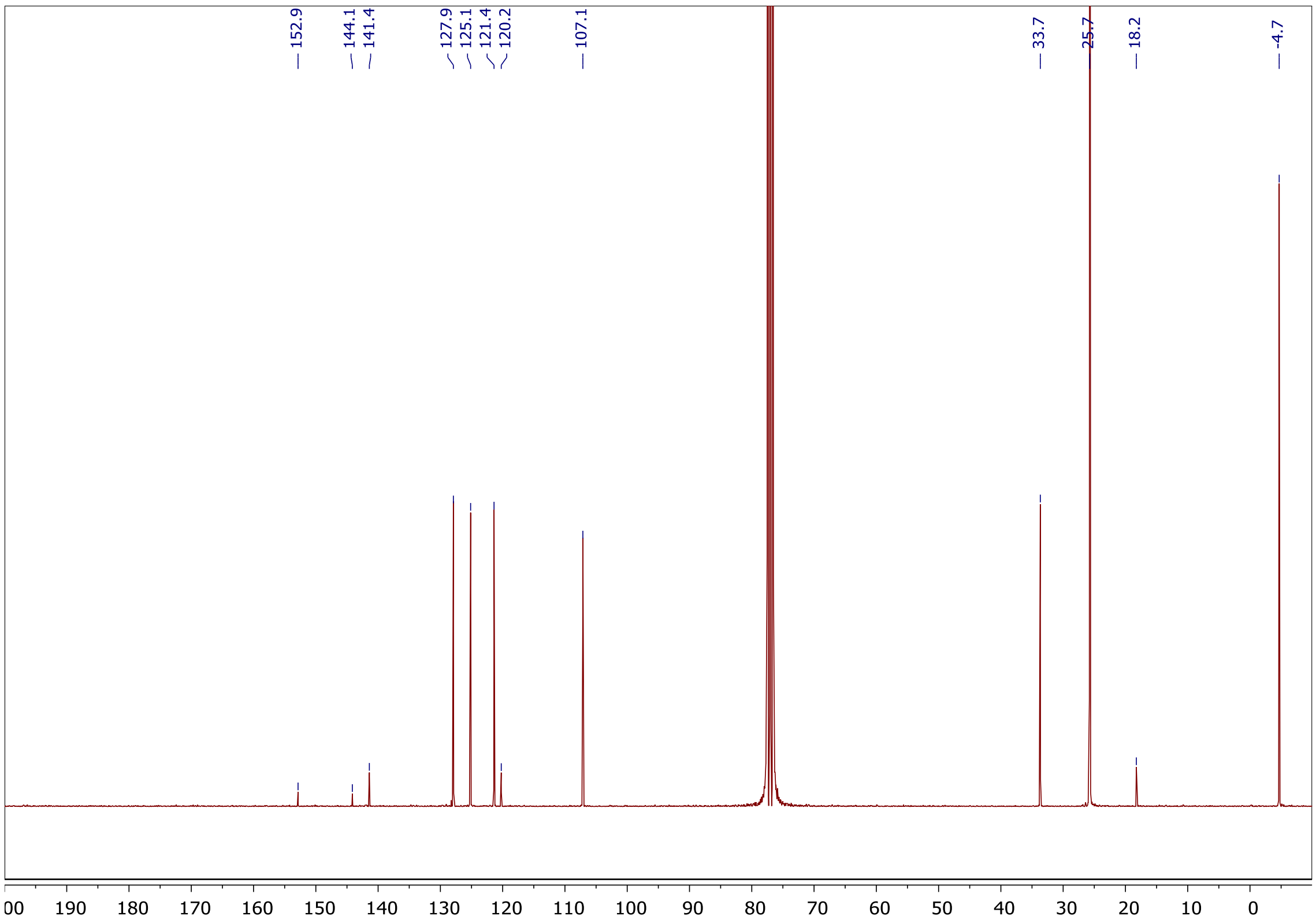

^{29}Si NMR (99 MHz, $CDCl_3$)

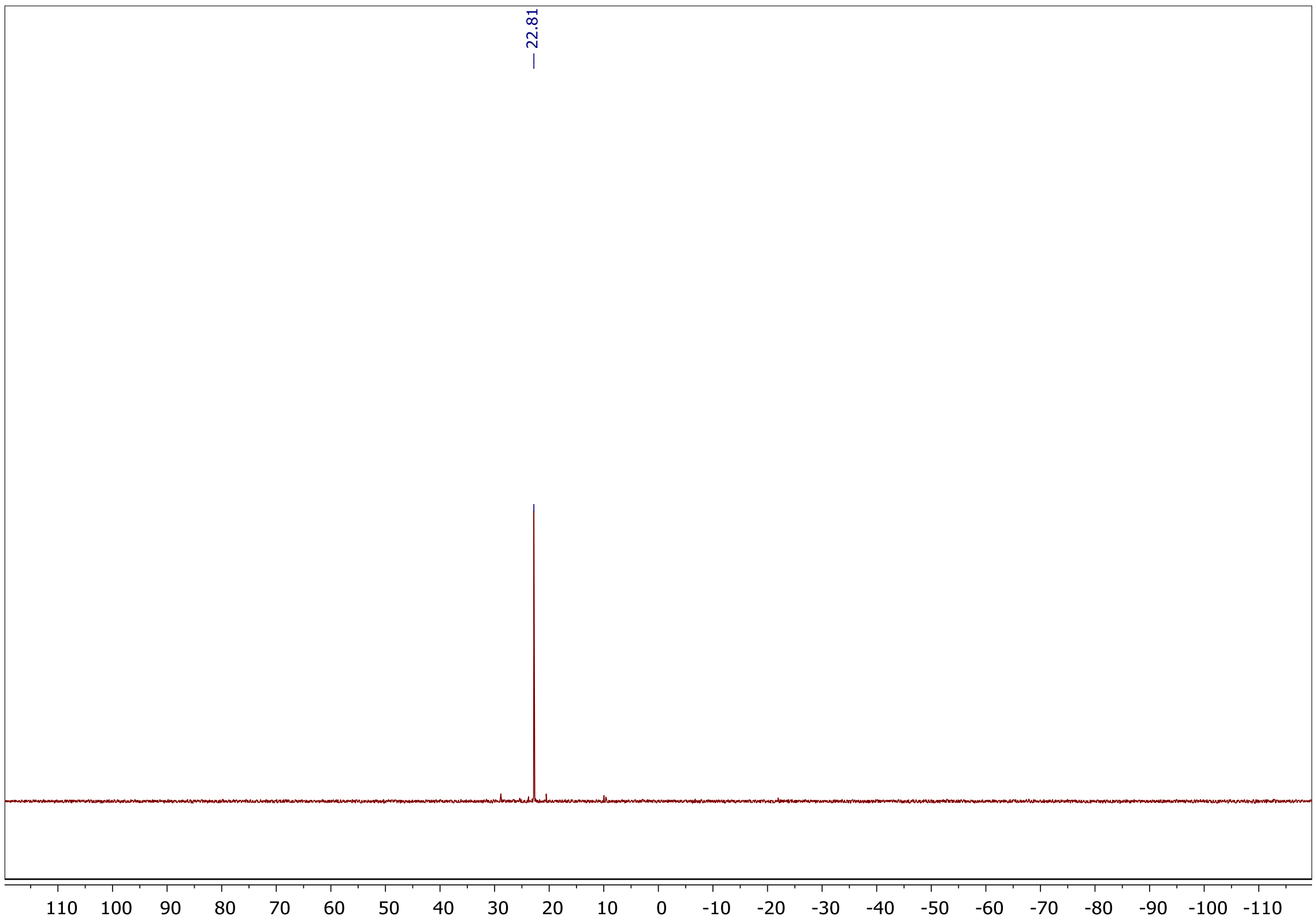

***tert*-Butyl 5-bromo-3-oxo-2,3-dihydrospiro[indene-1,4'-piperidine]-1'-carboxylate (23)**

^{1}H NMR (300 MHz, $CDCl_3$)

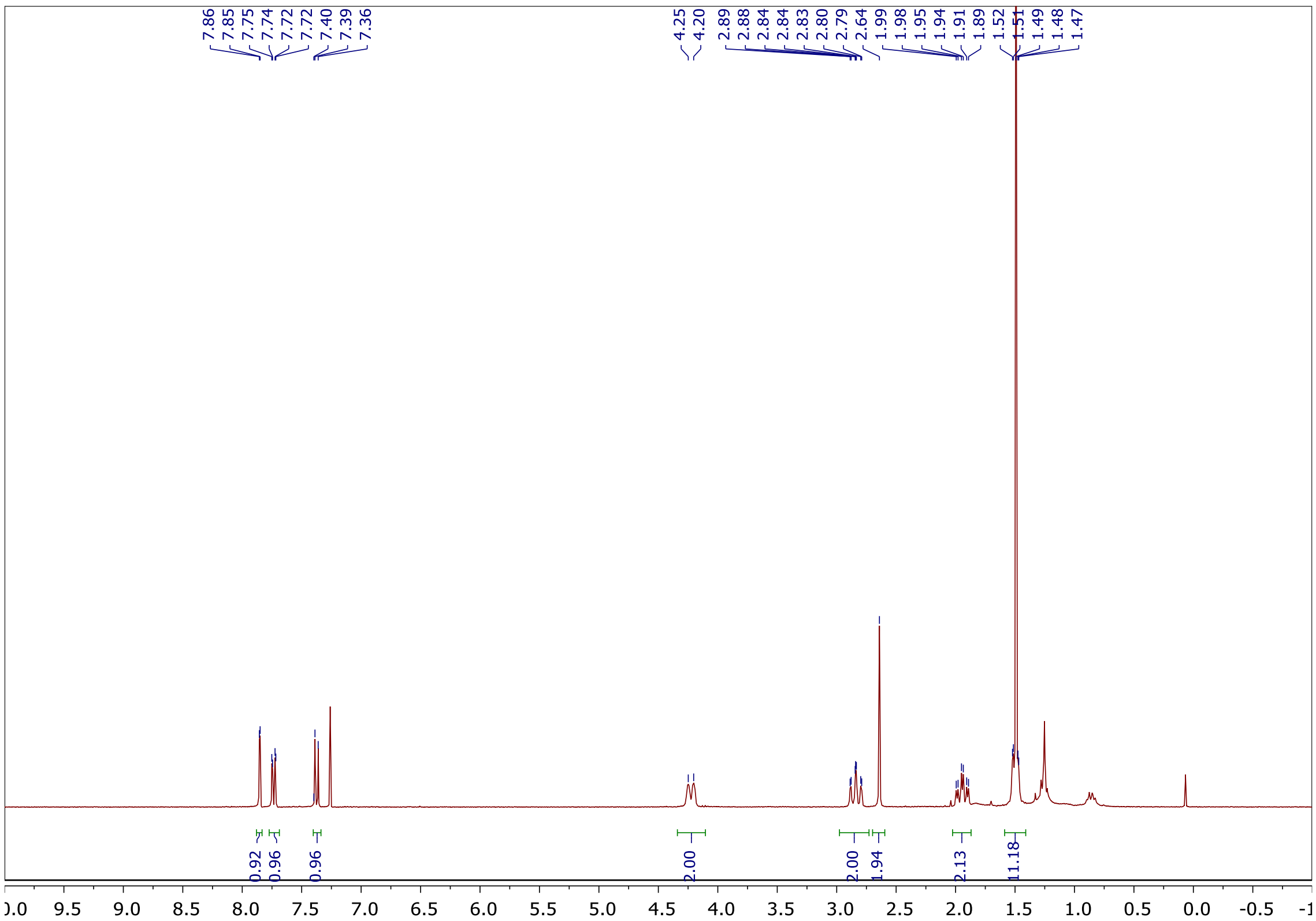

^{13}C NMR (75 MHz, $CDCl_3$)

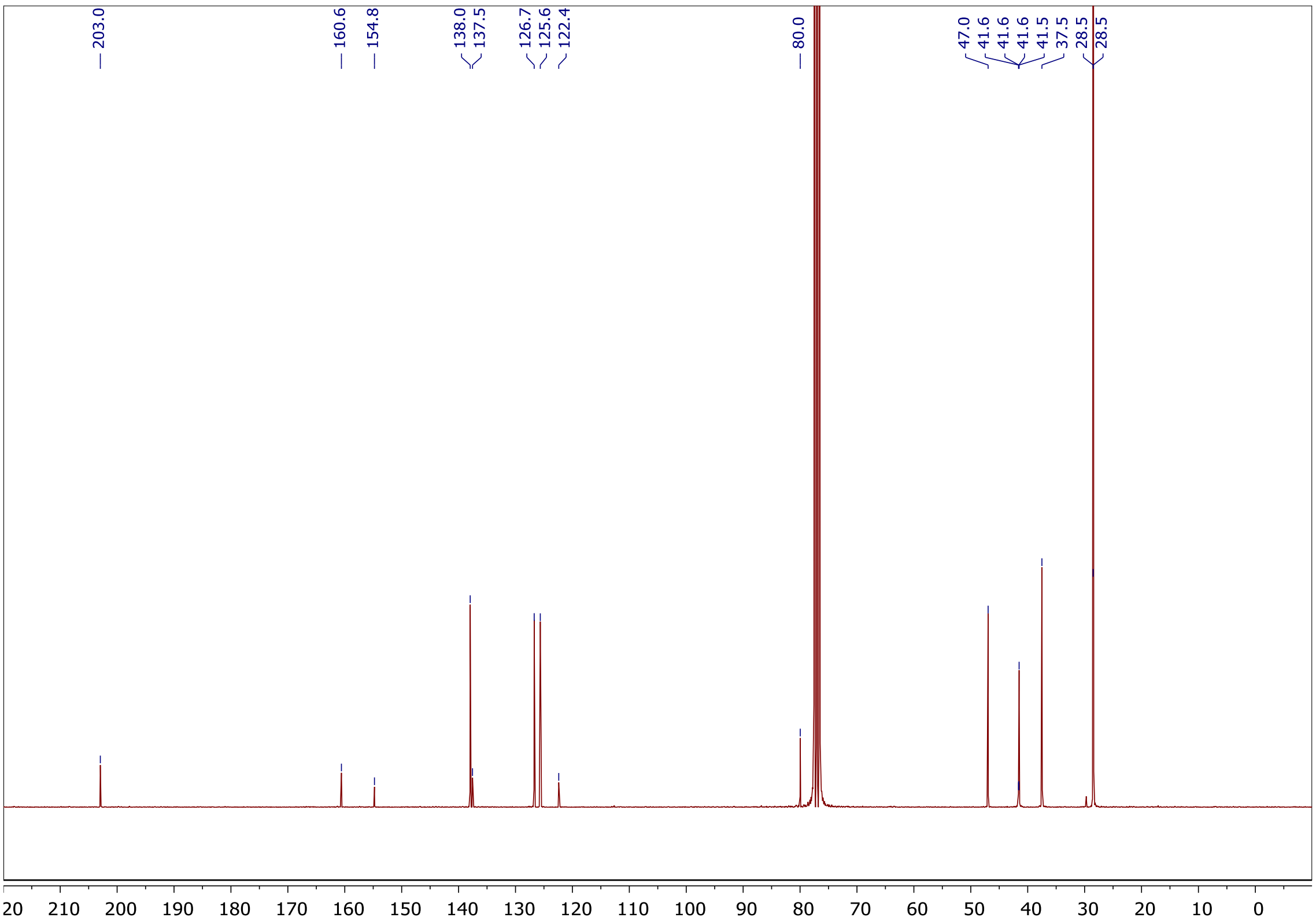

***tert*-Butyl 5-bromo-2,3-dihydrospiro[indene-1,4'-piperidine]-1'-carboxylate (13c)**

^{1}H NMR (300 MHz, $CDCl_3$)

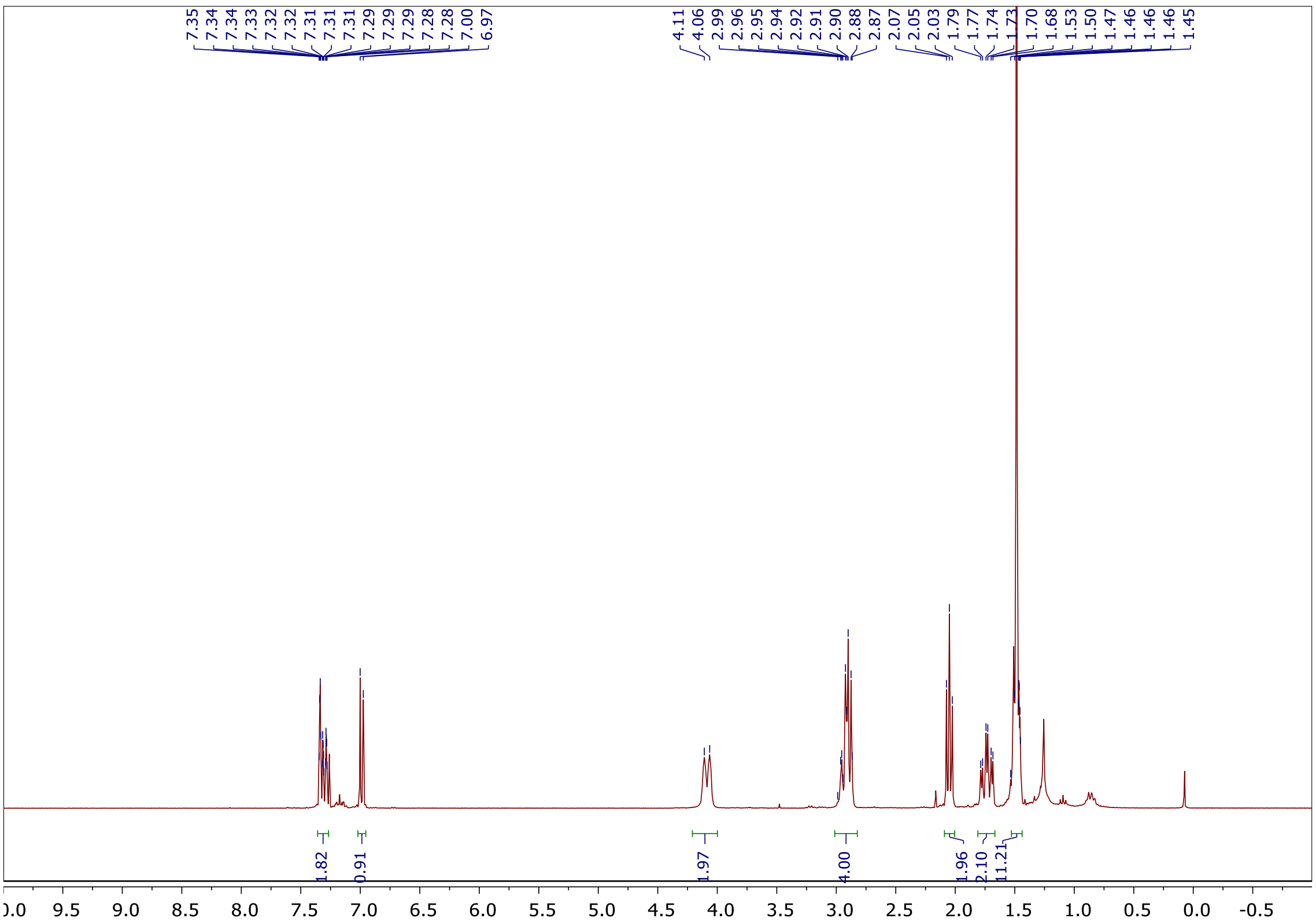

^{13}C NMR (75 MHz, $CDCl_3$)

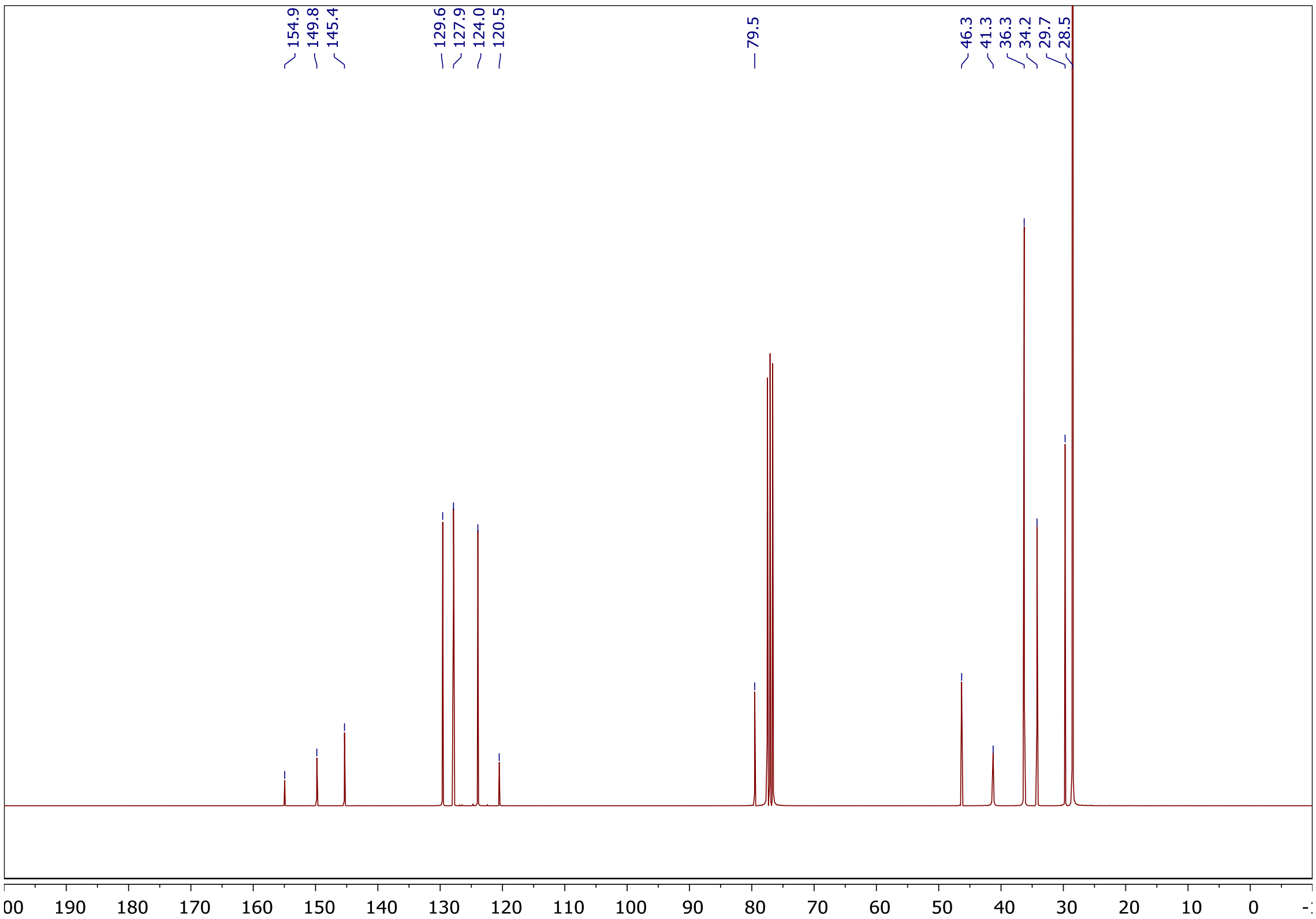

***tert*-Butyl 5-(3-chloro-4-[dimethylcarbamoyl]phenyl)-2,3-dihydrospiro[indene-1,4'-piperidine]-1'-carboxylate (14c)**

^{1}H NMR (500 MHz, CD_2Cl_2)

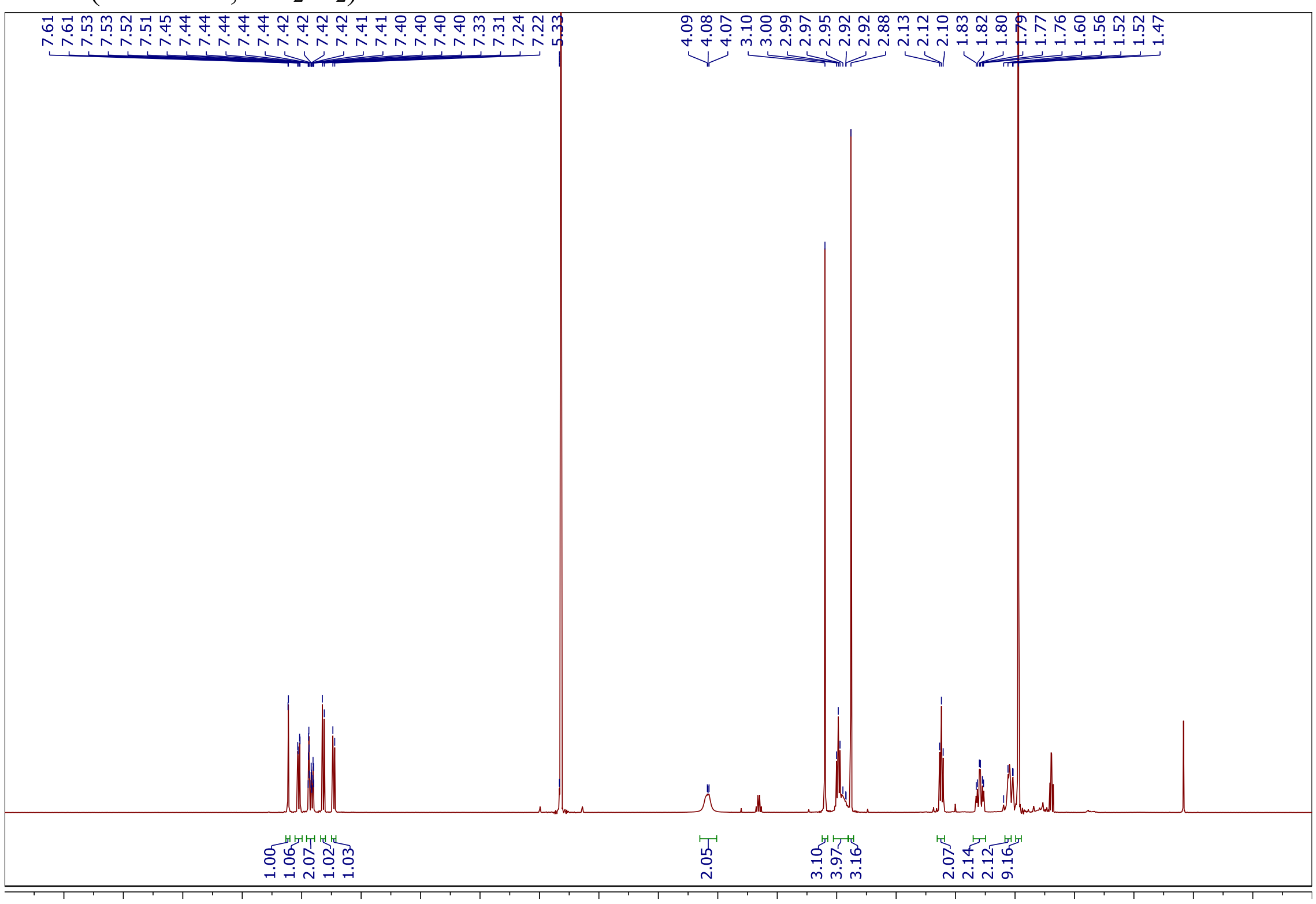

^{13}C NMR (126 MHz, CD_2Cl_2)

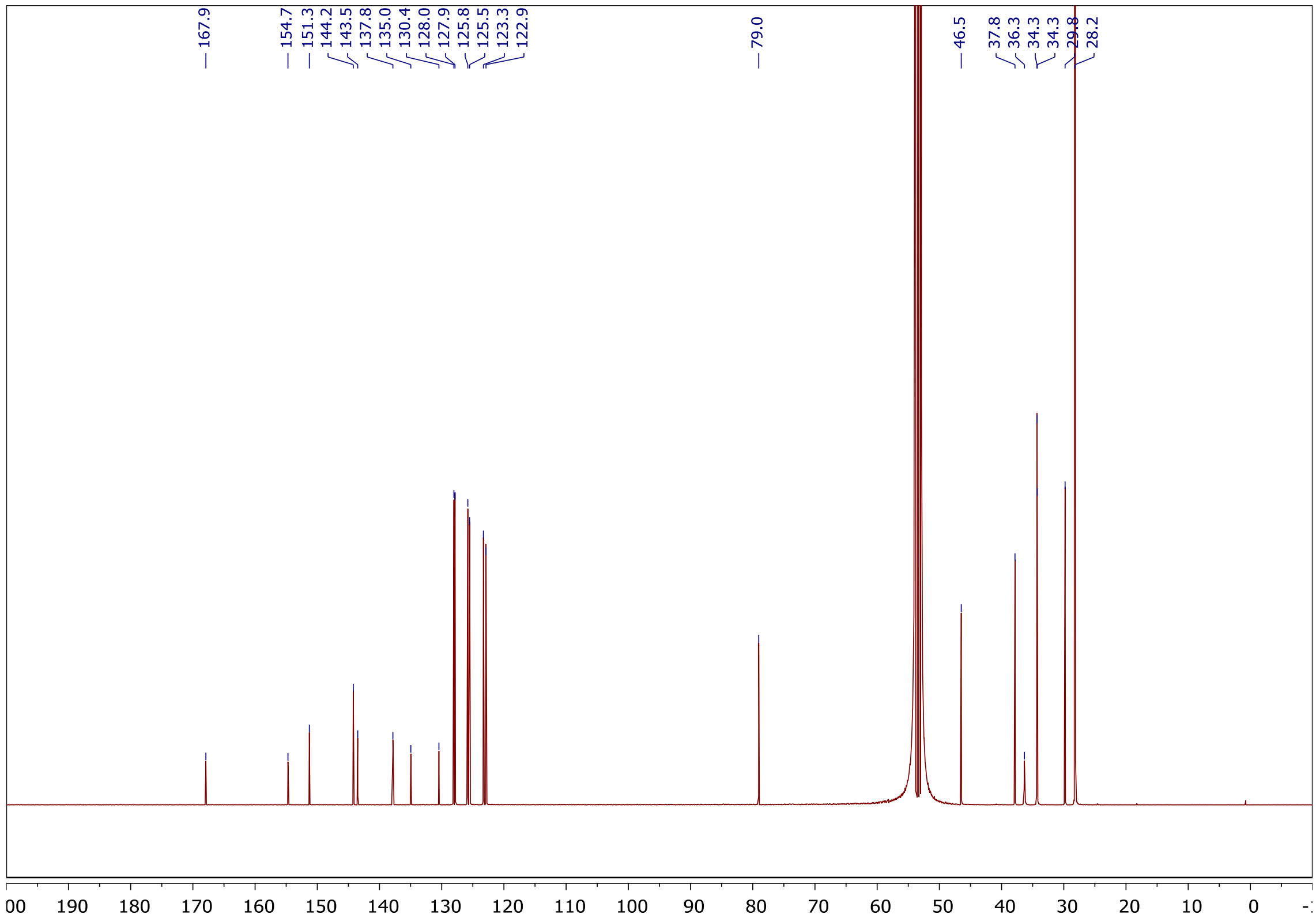

2-Chloro-*N,N*-dimethyl-4-(1'-[3-methyl-2-oxobutanoyl]-2,3-dihydrospiro[indene-1,4'-piperidin]-5-yl)benzamide (16c)

^{1}H NMR (300 MHz, CD_2Cl_2)

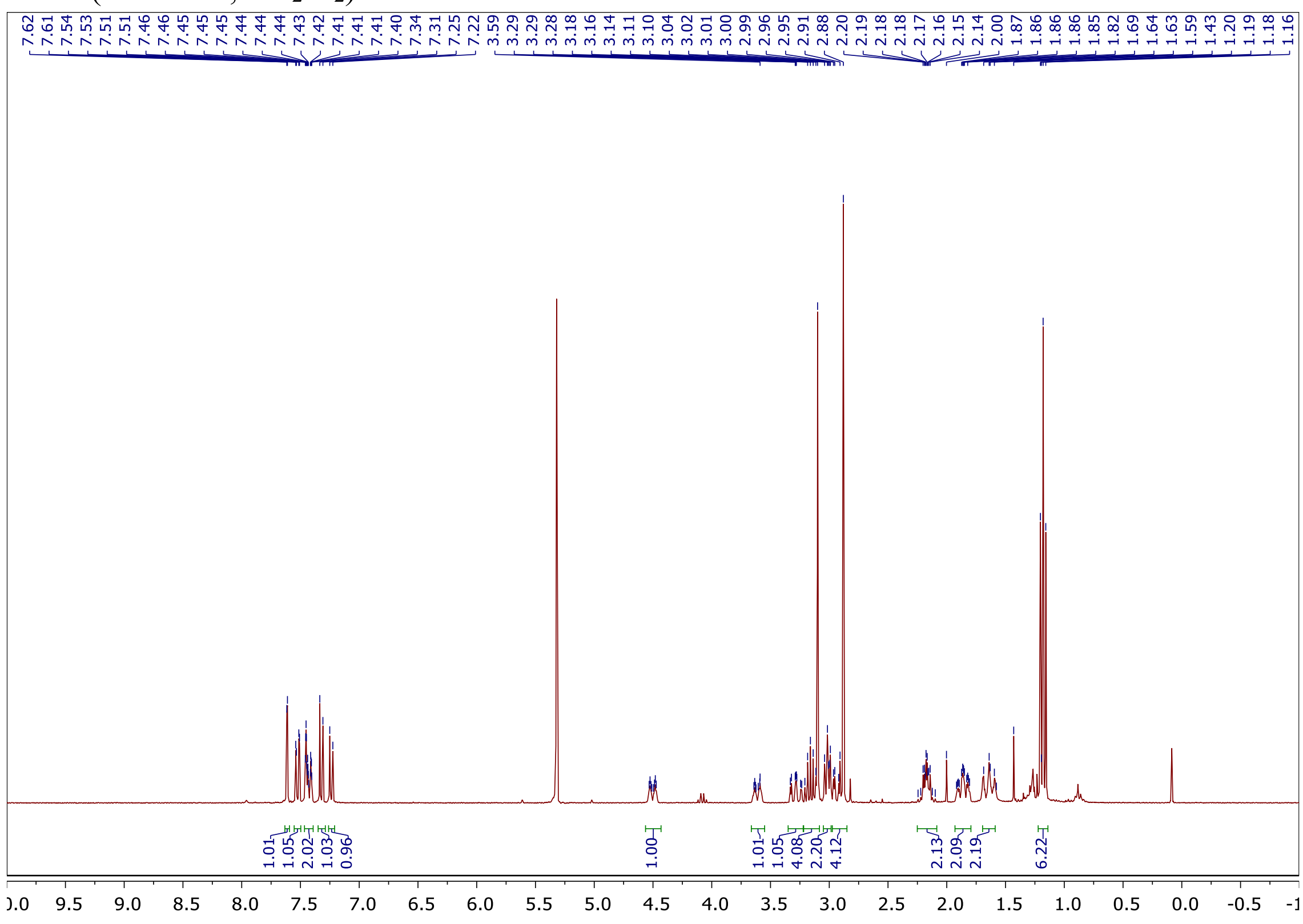

^{13}C NMR (75 MHz, CD_2Cl_2)

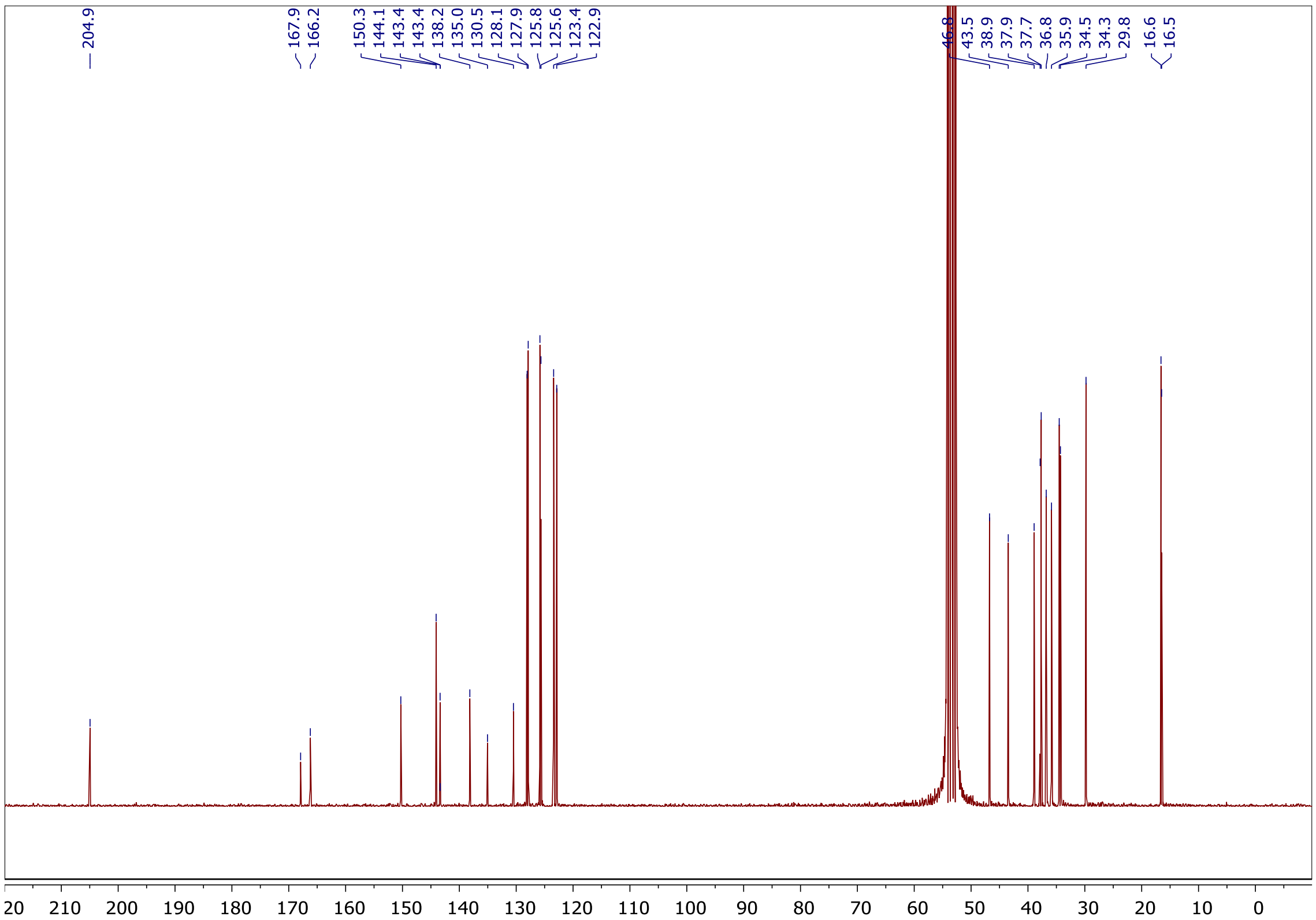

2-Chloro-4-(1'-[2-hydroxy-3-methyl-2-{trifluoromethyl}butanoyl]-2,3-dihydrospiro[indene-1,4'-piperidin]-5-yl)-*N,N*-dimethylbenzamide (*rac*-7)

^{1}H NMR (500 MHz, CD_2Cl_2)

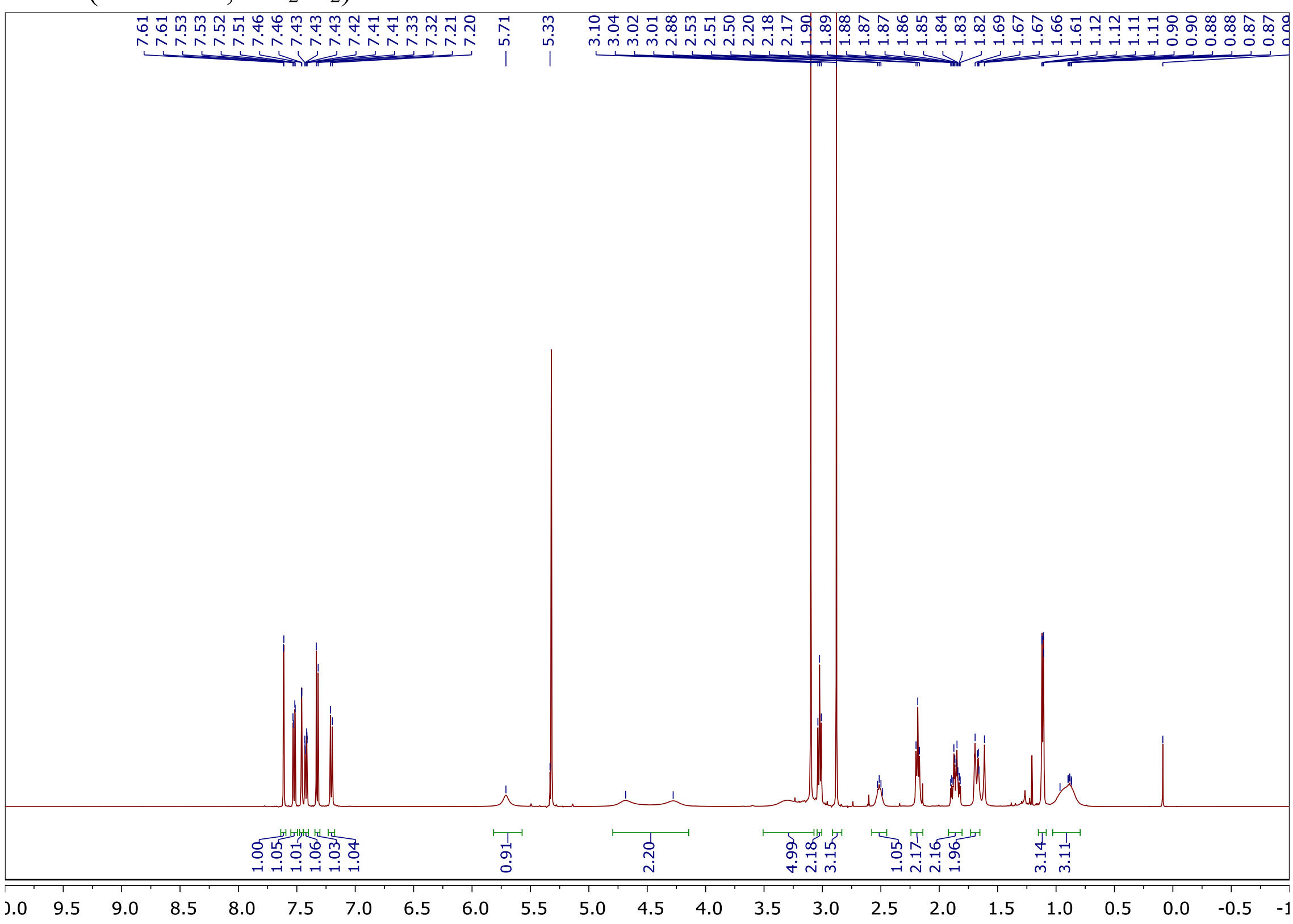

^{13}C NMR (126 MHz, CD_2Cl_2)

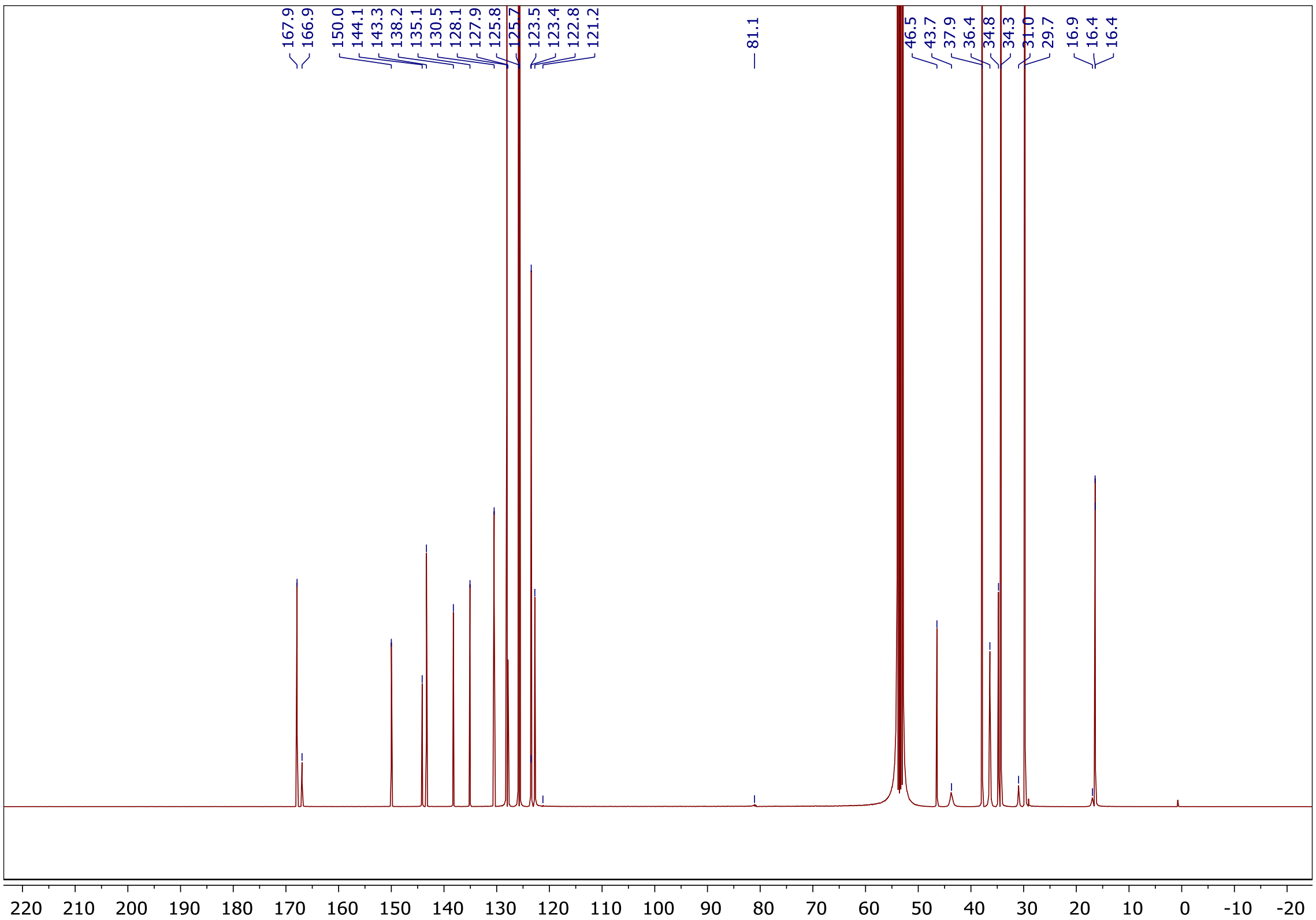

^{19}F NMR (471 MHz, CD_2Cl_2)

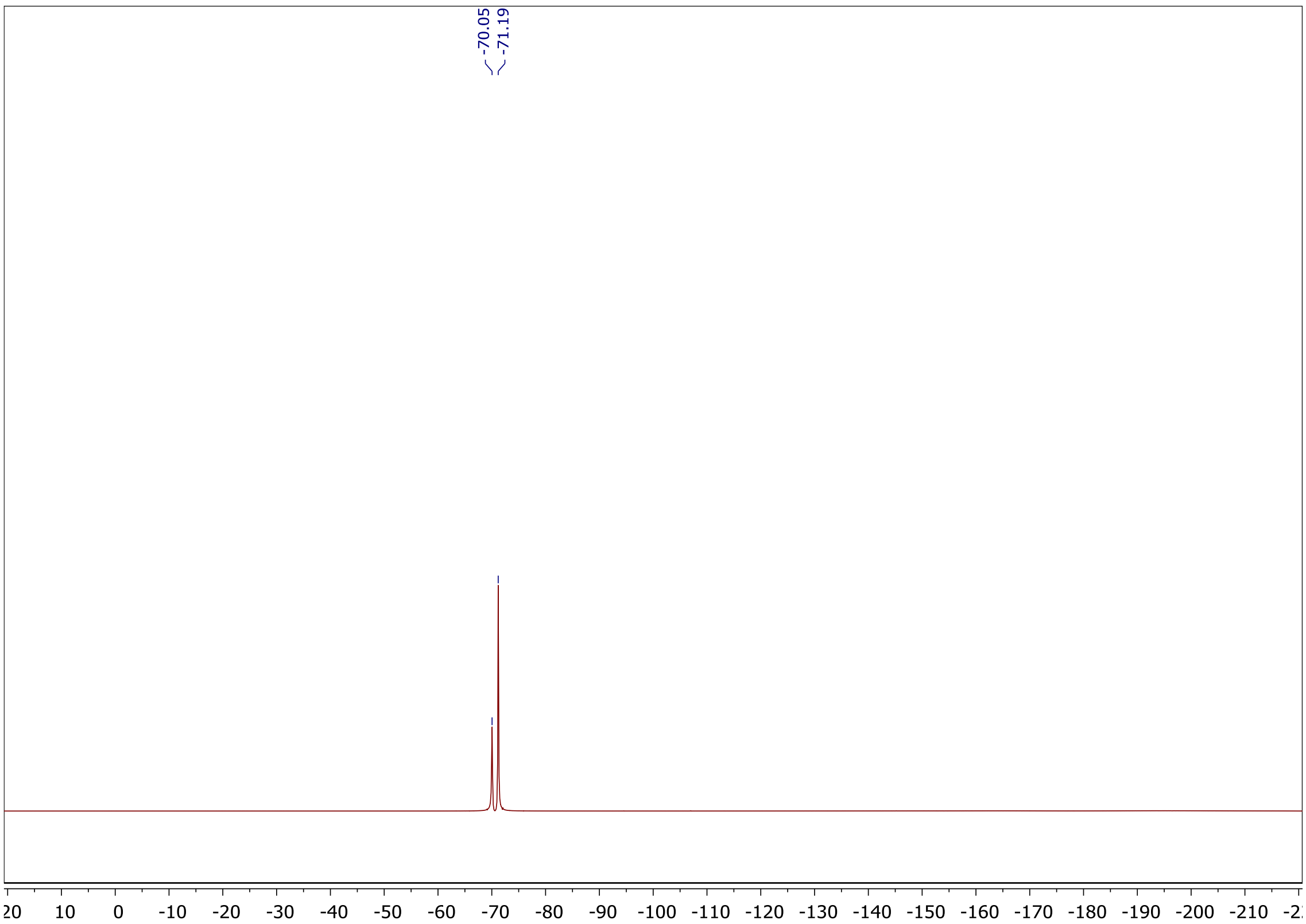

HPLC Traces

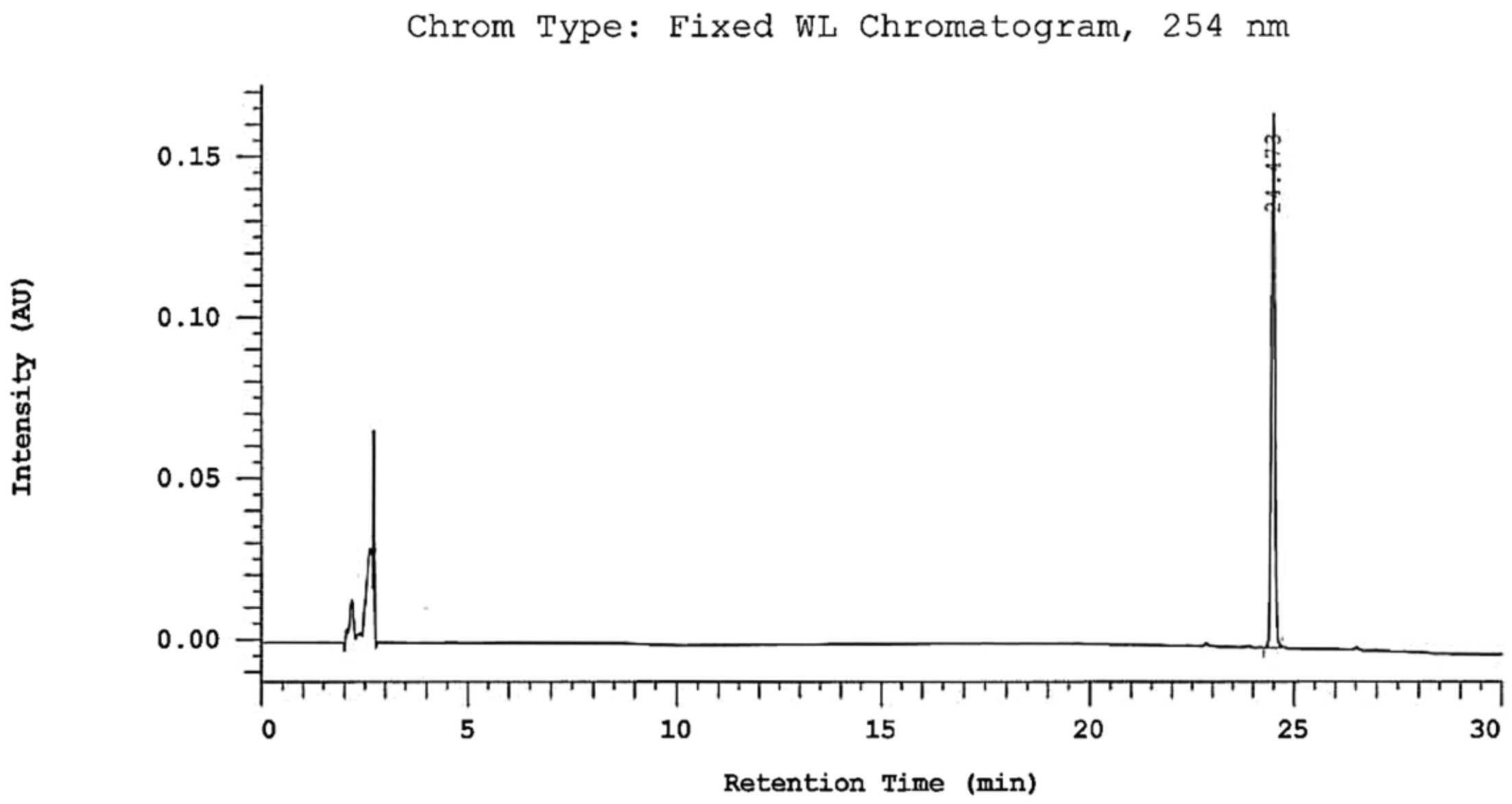

No.	RT	Area	Area %	Height
1	24.473	464334	100.000	82943
		464334	100.000	82943

Peak rejection level: 0

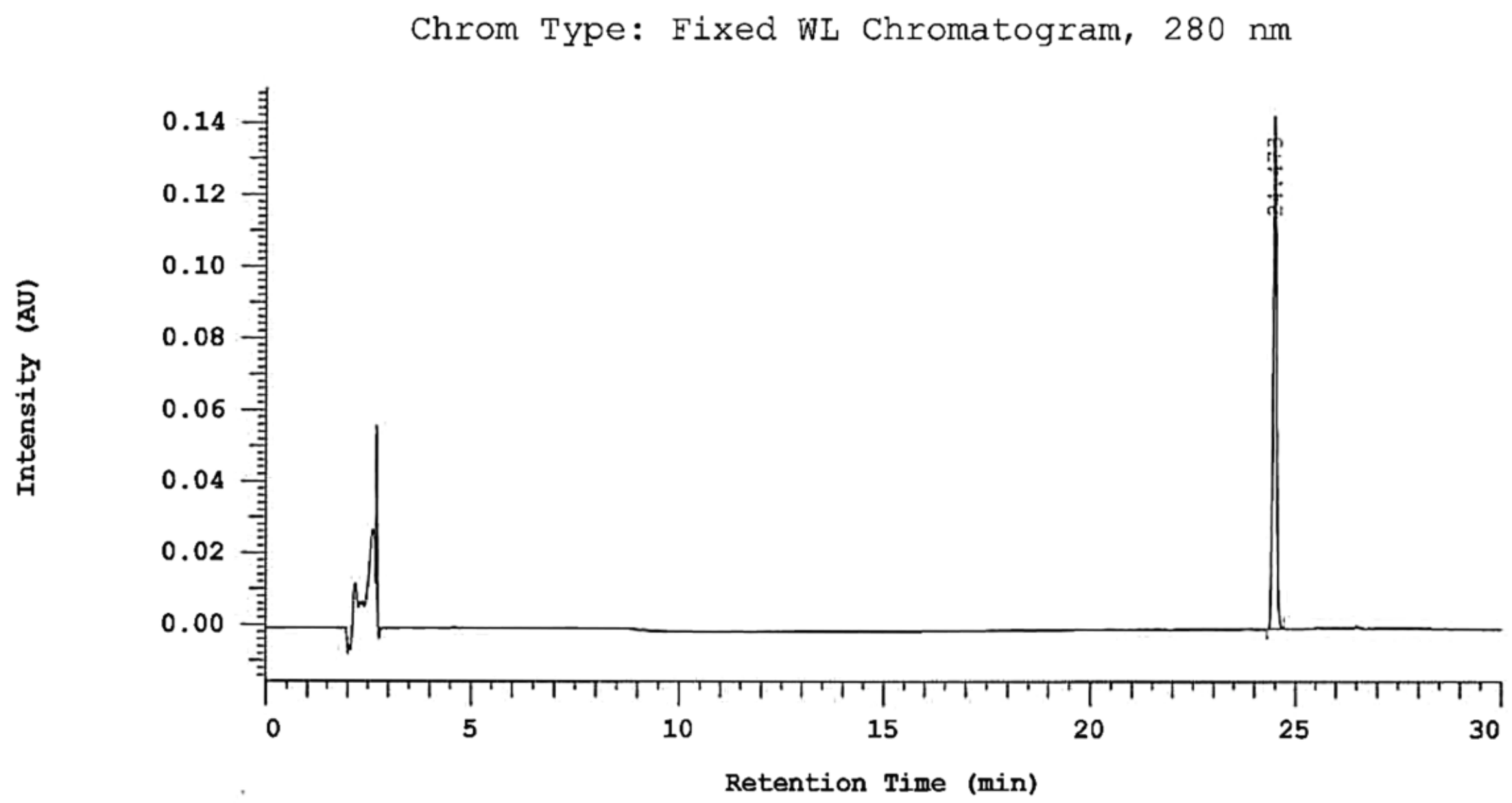

No.	RT	Area	Area %	Height
1	24.473	399277	100.000	71476
		399277	100.000	71476

Peak rejection level: 0

tert-Butyl 4-hydroxy-4-([phenylsulfinyl]methyl)piperidine-1-carboxylate (26)

^{1}H NMR (300 MHz, $CDCl_3$)

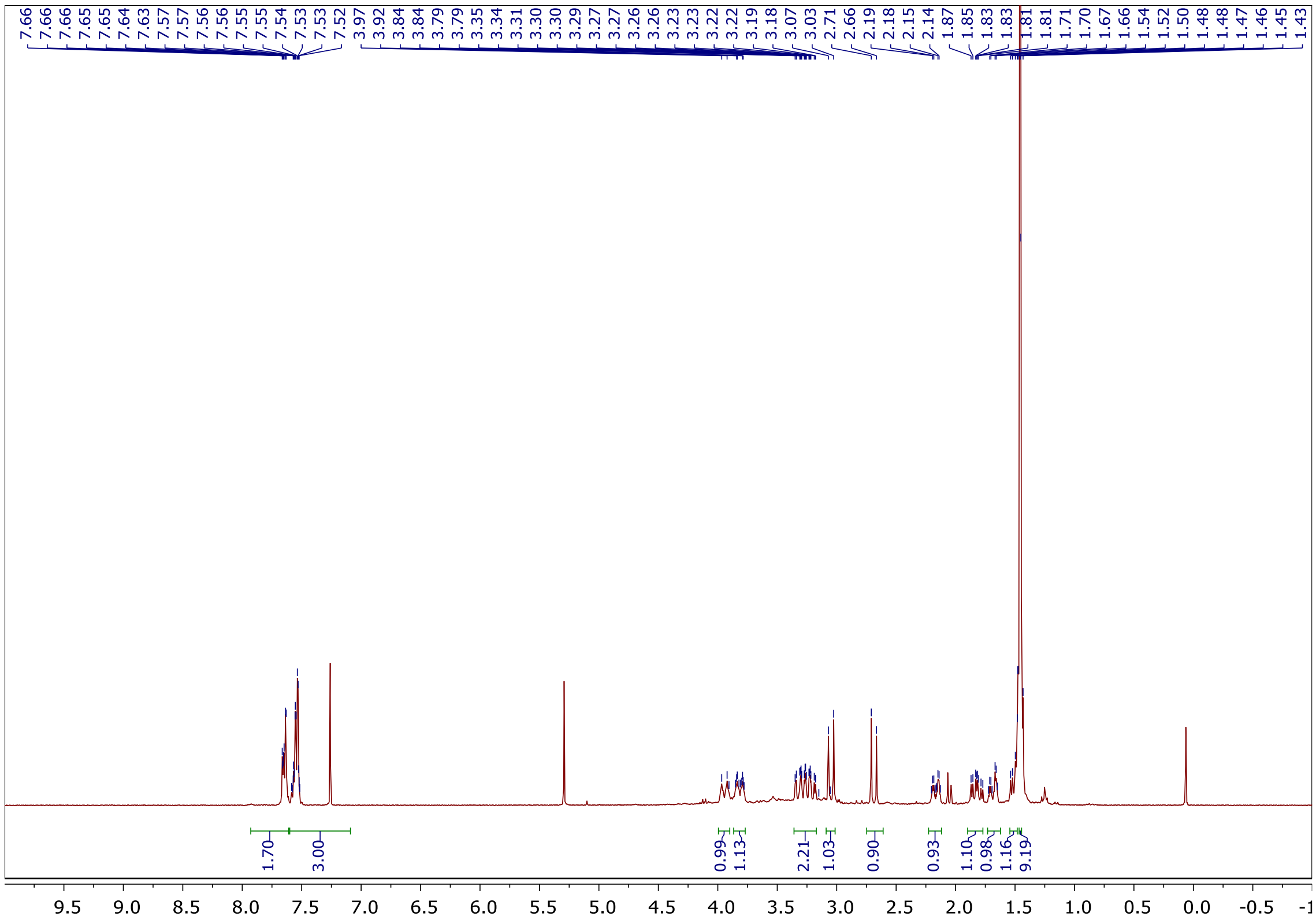

^{13}C NMR (75 MHz, $CDCl_3$)

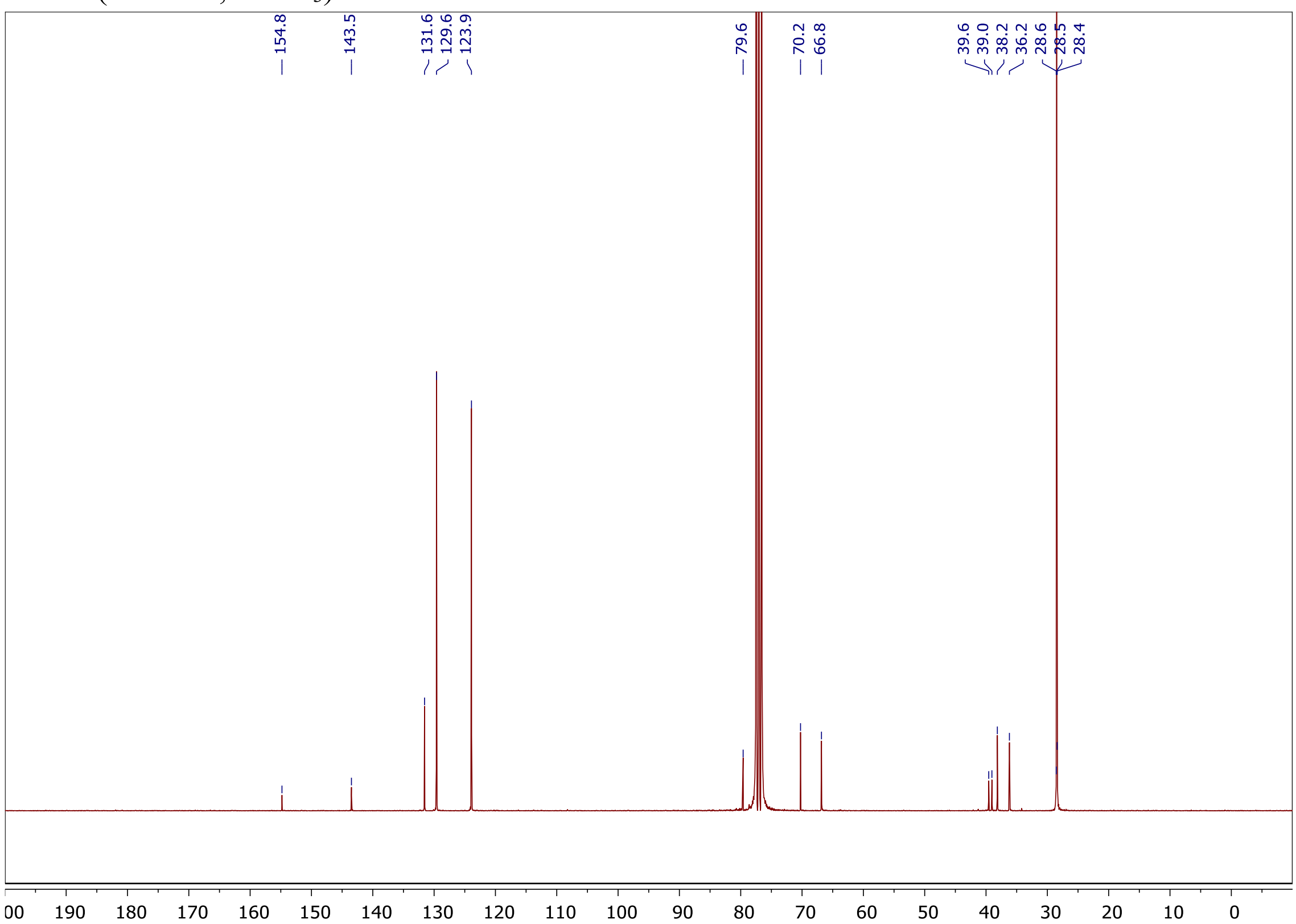

***tert*-Butyl 3-hydroxy-4-methylenepiperidine-1-carboxylate (27)**

^{1}H NMR (400 MHz, $CDCl_3$)

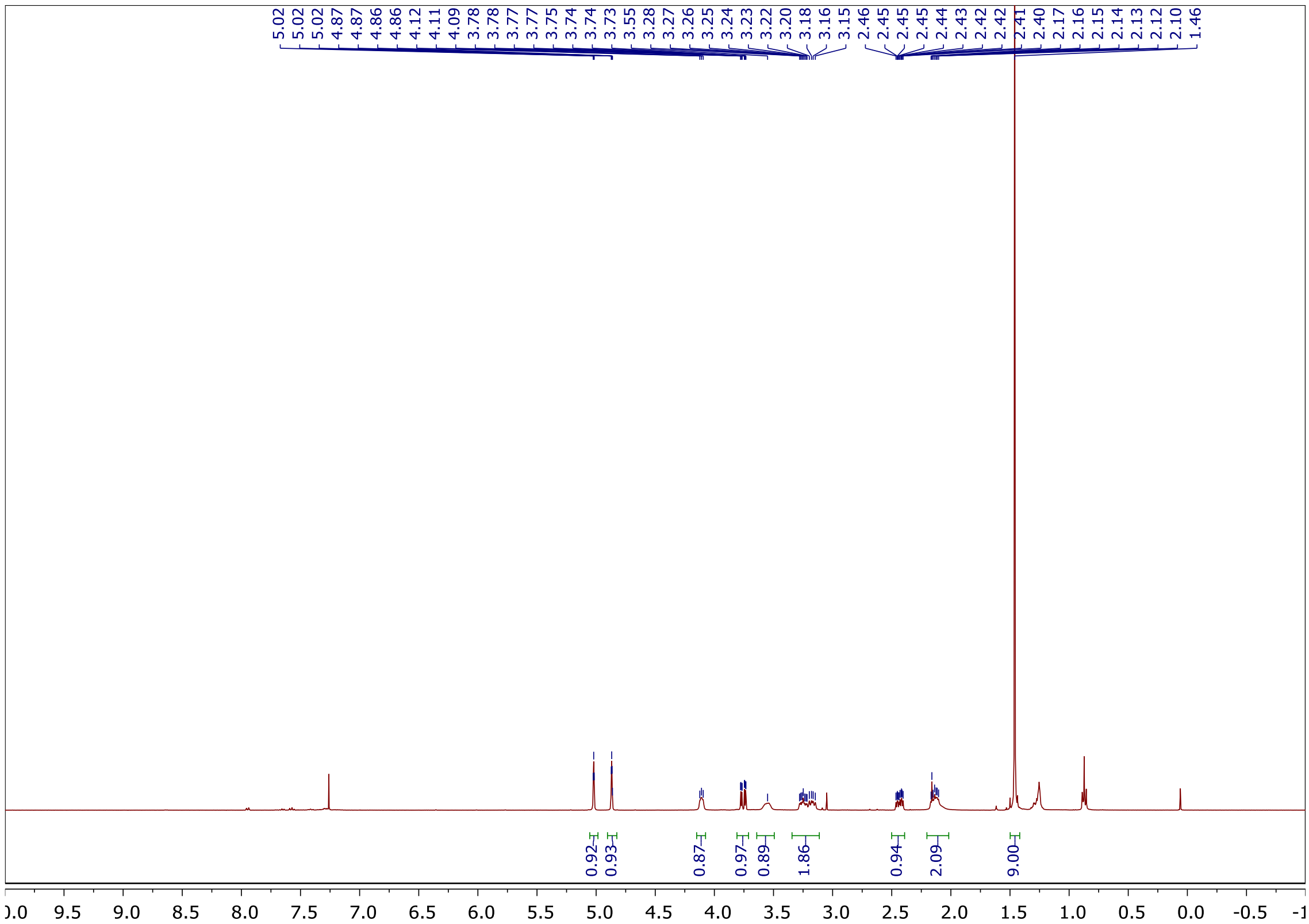

^{13}C NMR (75 MHz, $CDCl_3$)

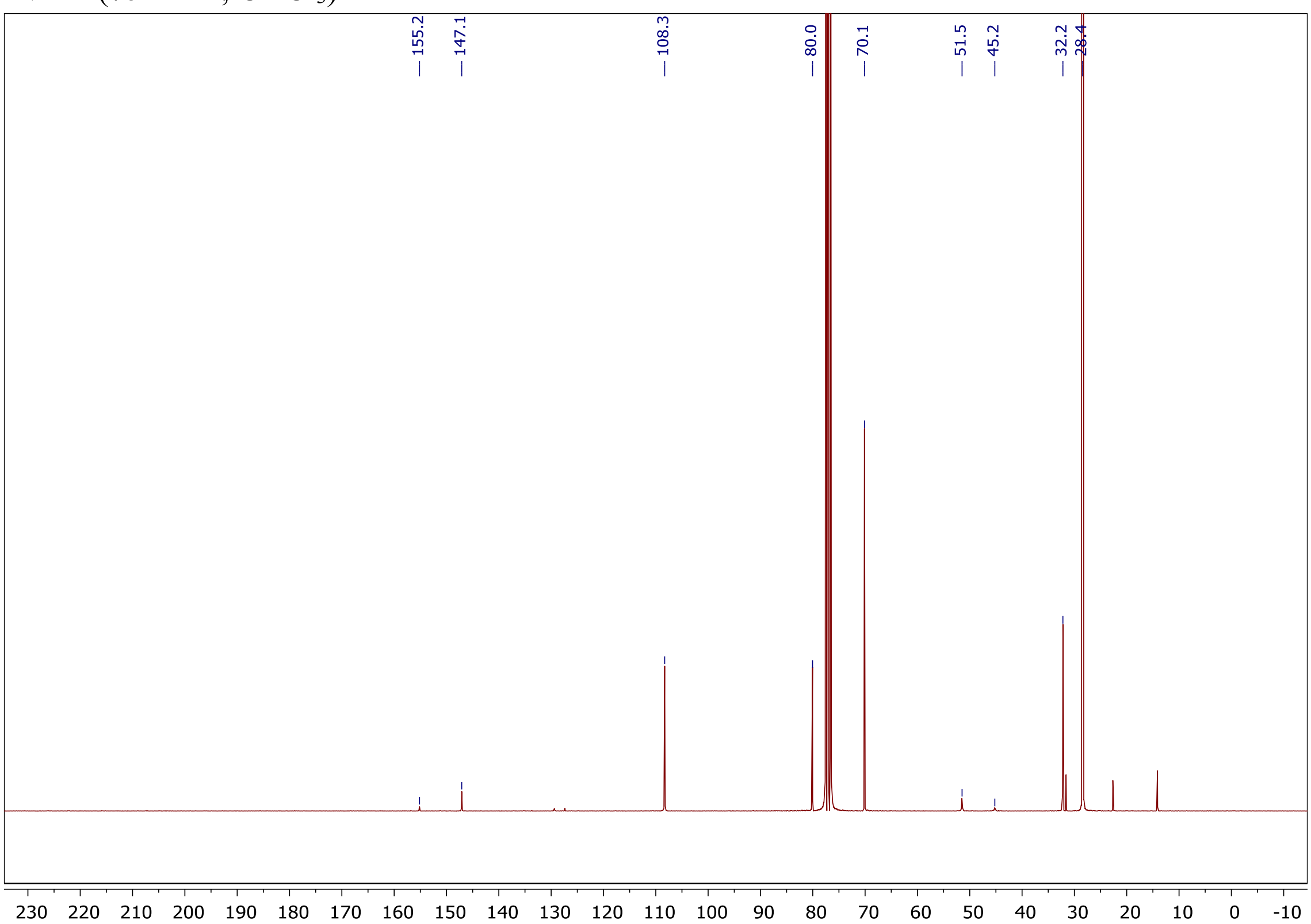

tert-Butyl 4-(chloromethyl)-3,6-dihydropyridine-1(2H)-carboxylate (28)

^{1}H NMR (300 MHz, $CDCl_3$)

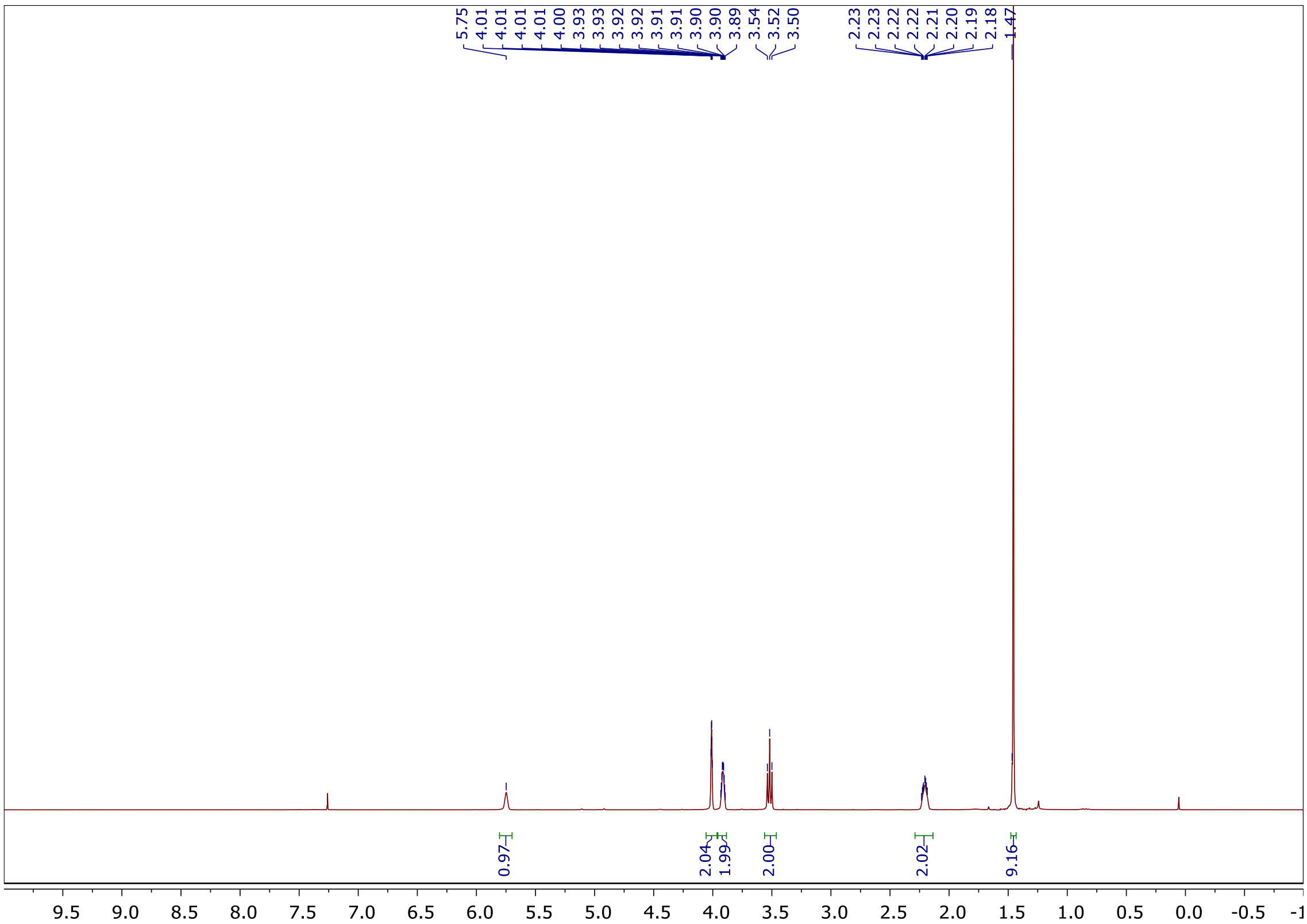

^{13}C NMR (75 MHz, $CDCl_3$)

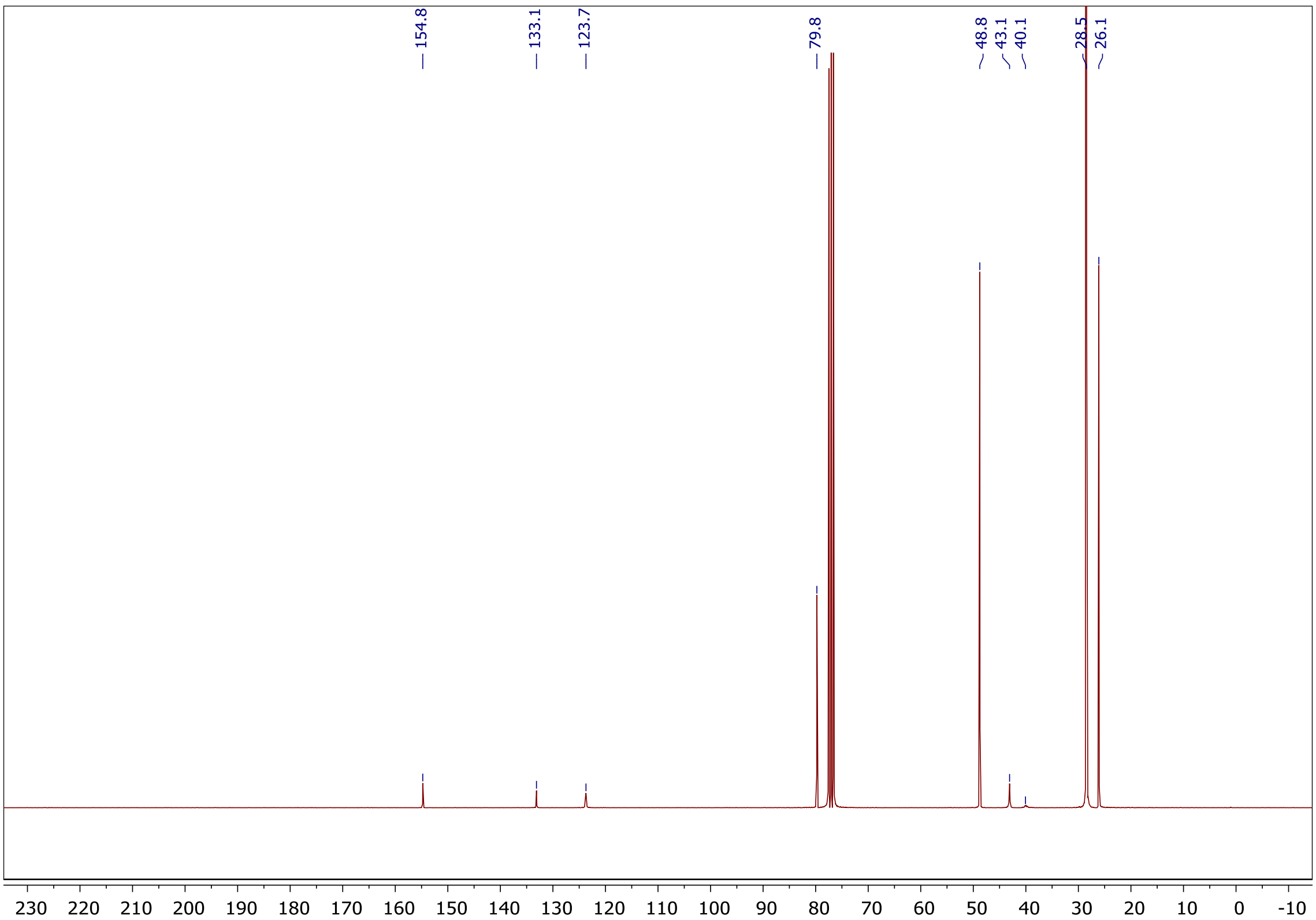

***tert*-Butyl 4-([5-bromo-2-iodophenoxy]methyl)-3,6-dihydropyridine-1(2*H*)-carboxylate (30)**

^{1}H NMR (400 MHz, $CDCl_3$)

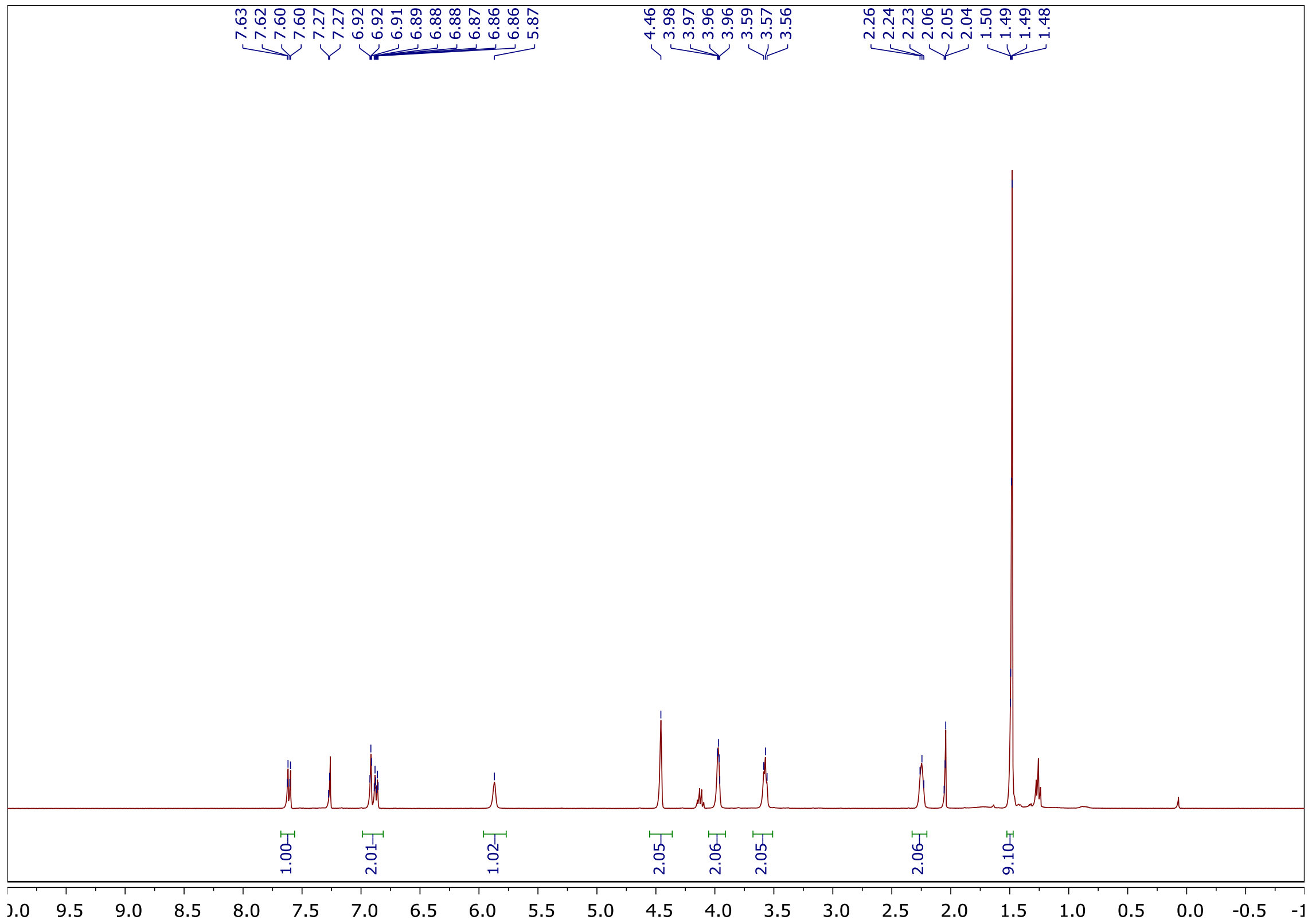

^{13}C NMR (101 MHz, $CDCl_3$)

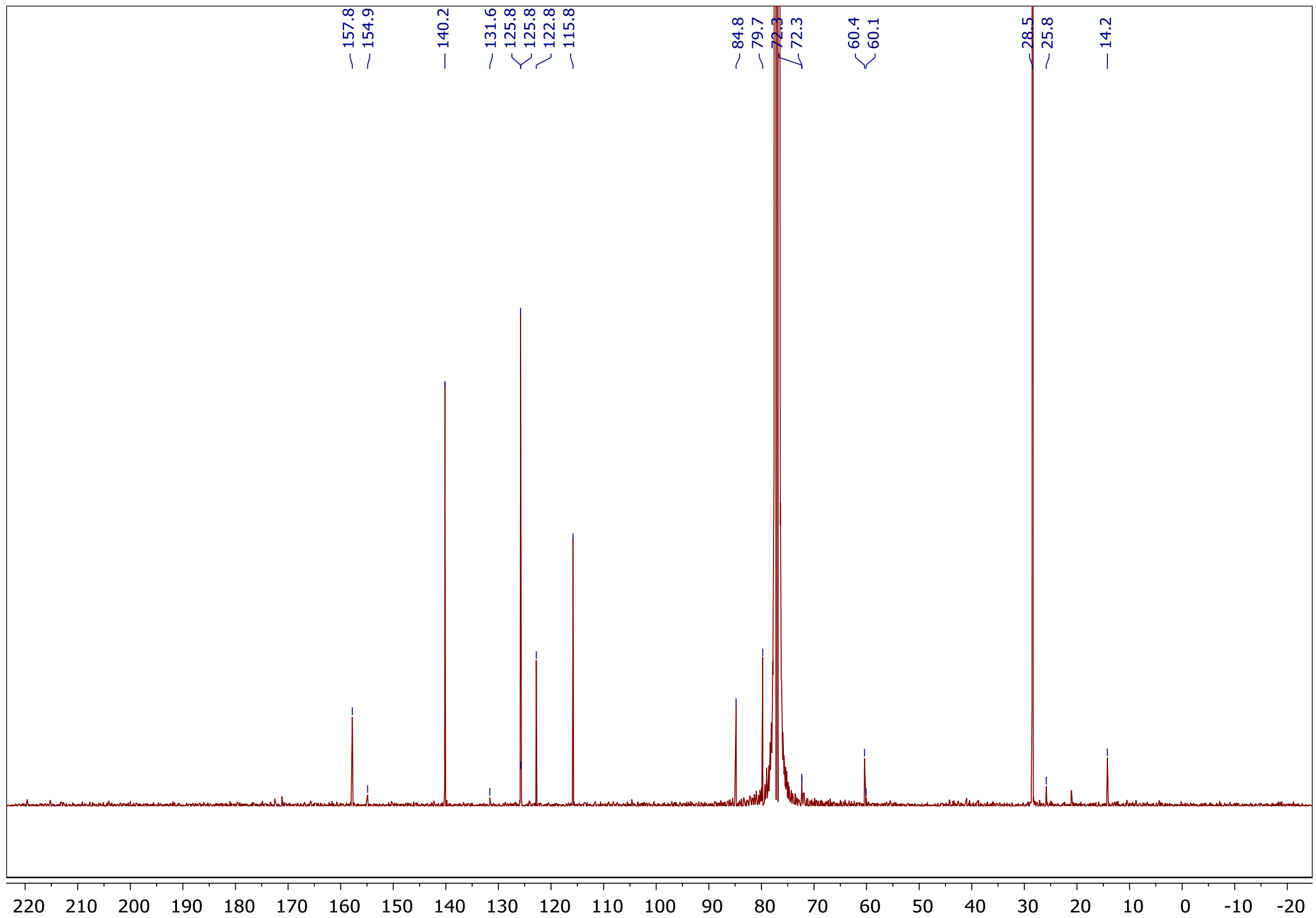

2,4,6-Tris(diphenylamino)-5-fluoroisophthalonitrile (31)

^{1}H NMR (300 MHz, $CDCl_3$)

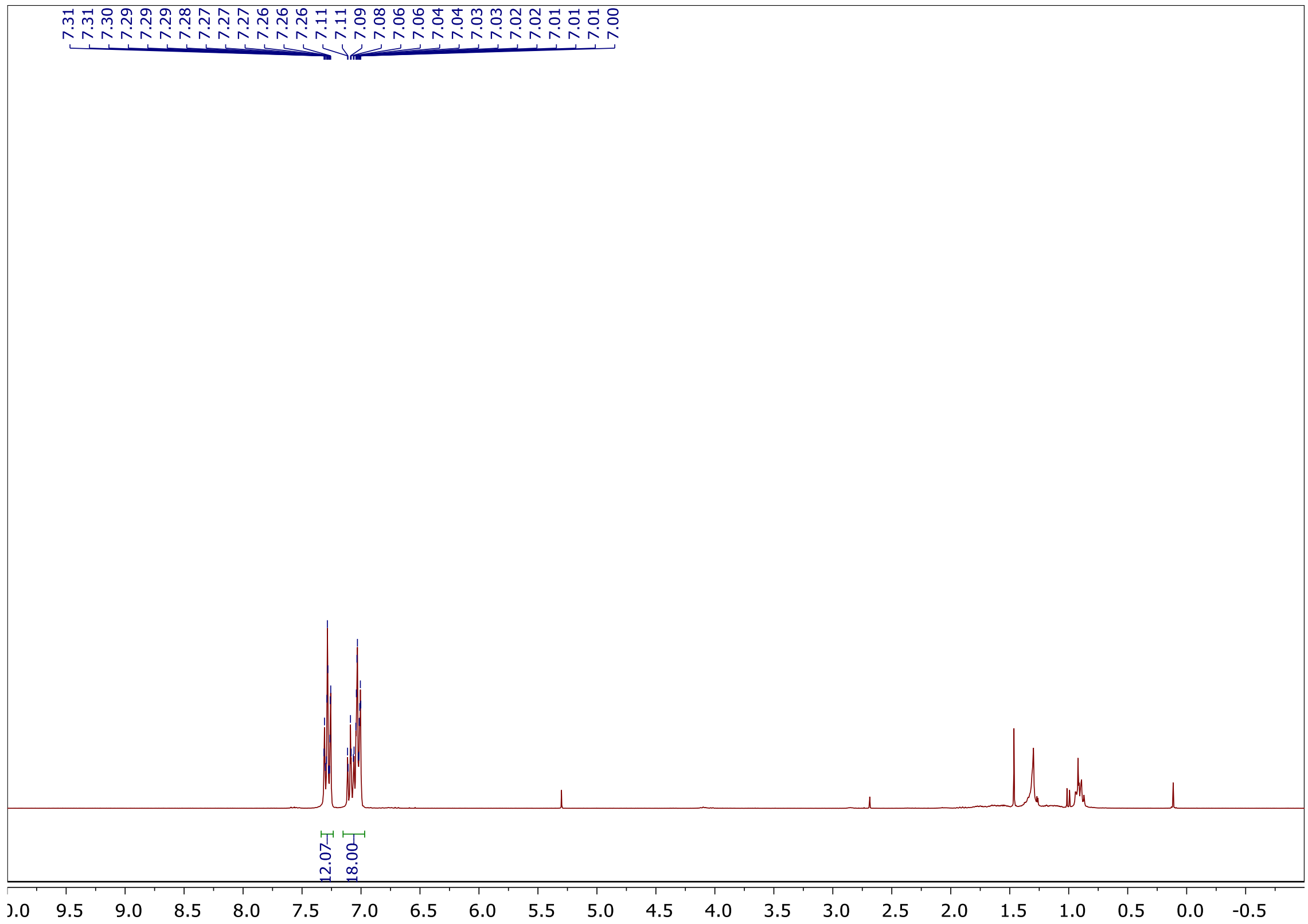

^{13}C NMR (75 MHz, $CDCl_3$)

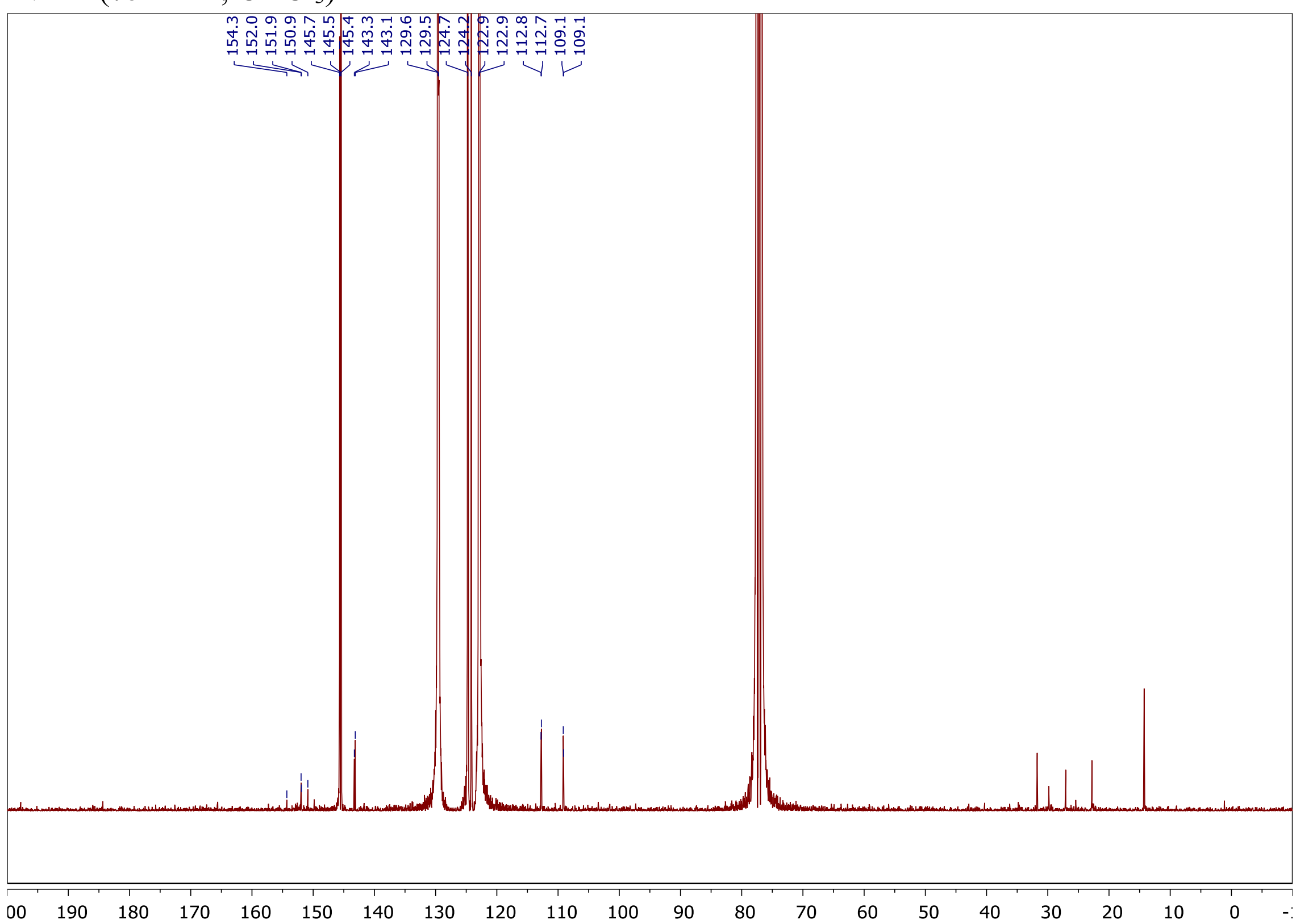

^{19}F NMR (282 MHz, $CDCl_3$)

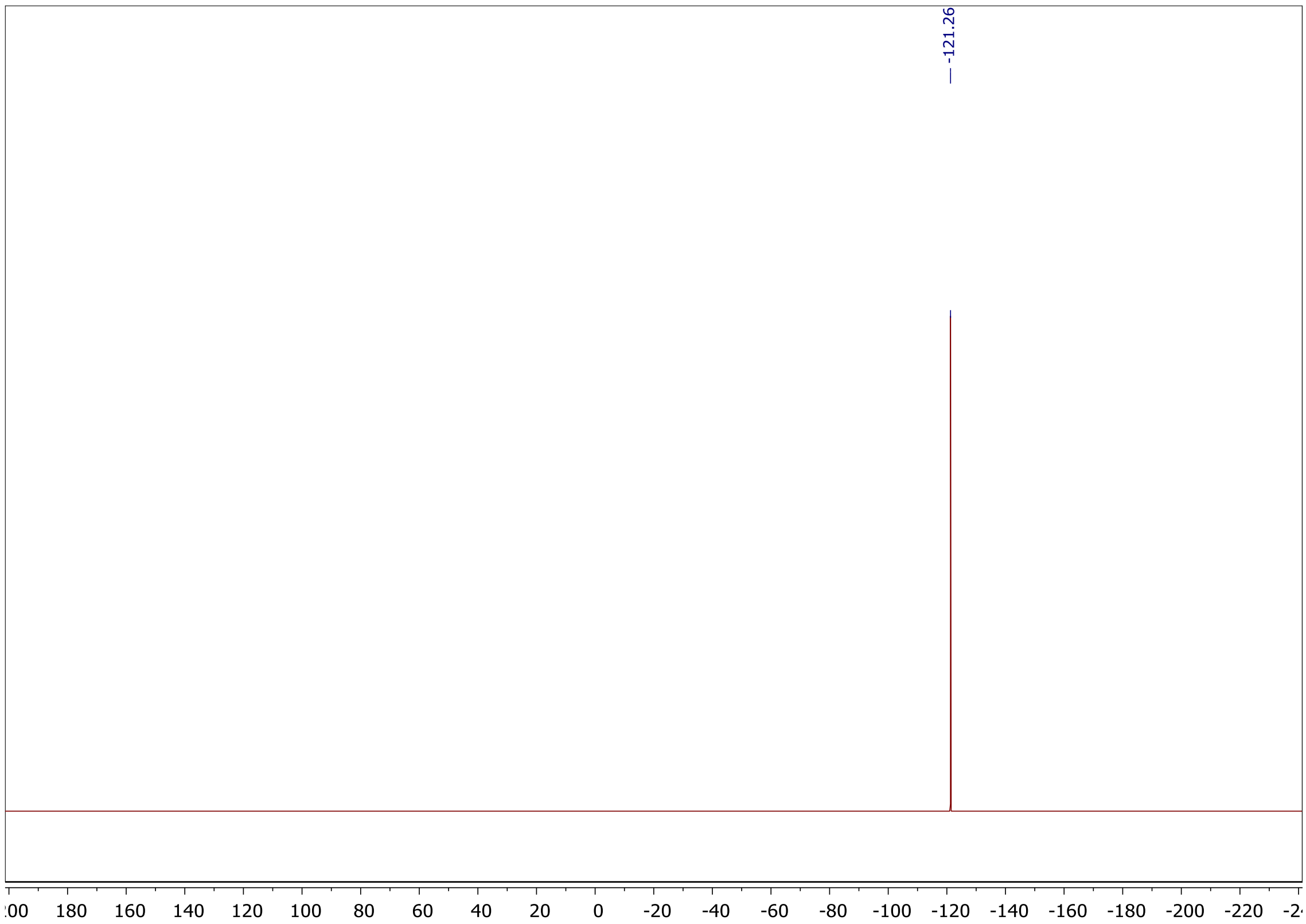

***tert*-Butyl 6-bromo-2*H*-spiro[benzofuran-3,4'-piperidine]-1'-carboxylate (13d)**

^{1}H NMR (300 MHz, $CDCl_3$)

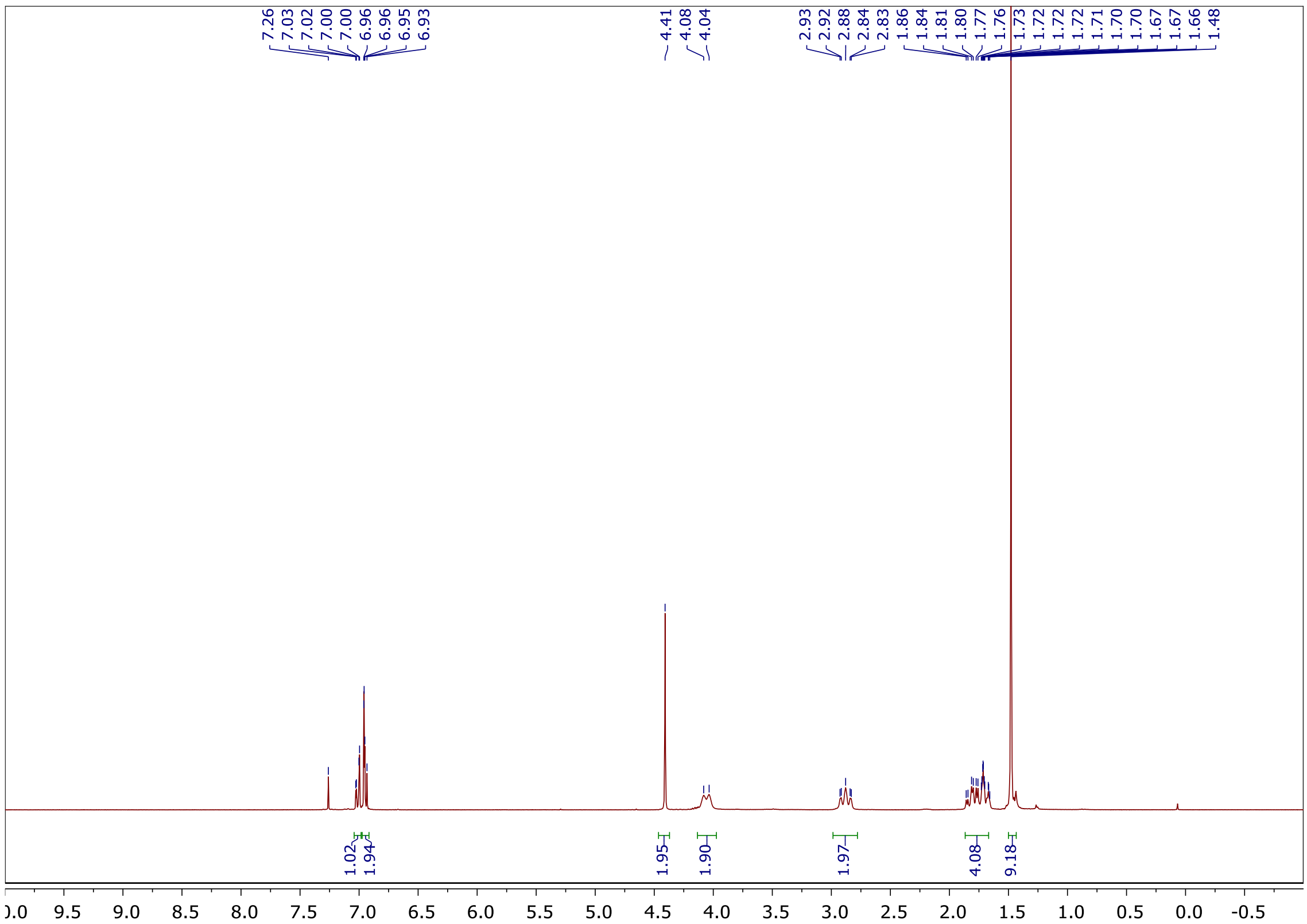

^{13}C NMR (75 MHz, $CDCl_3$)

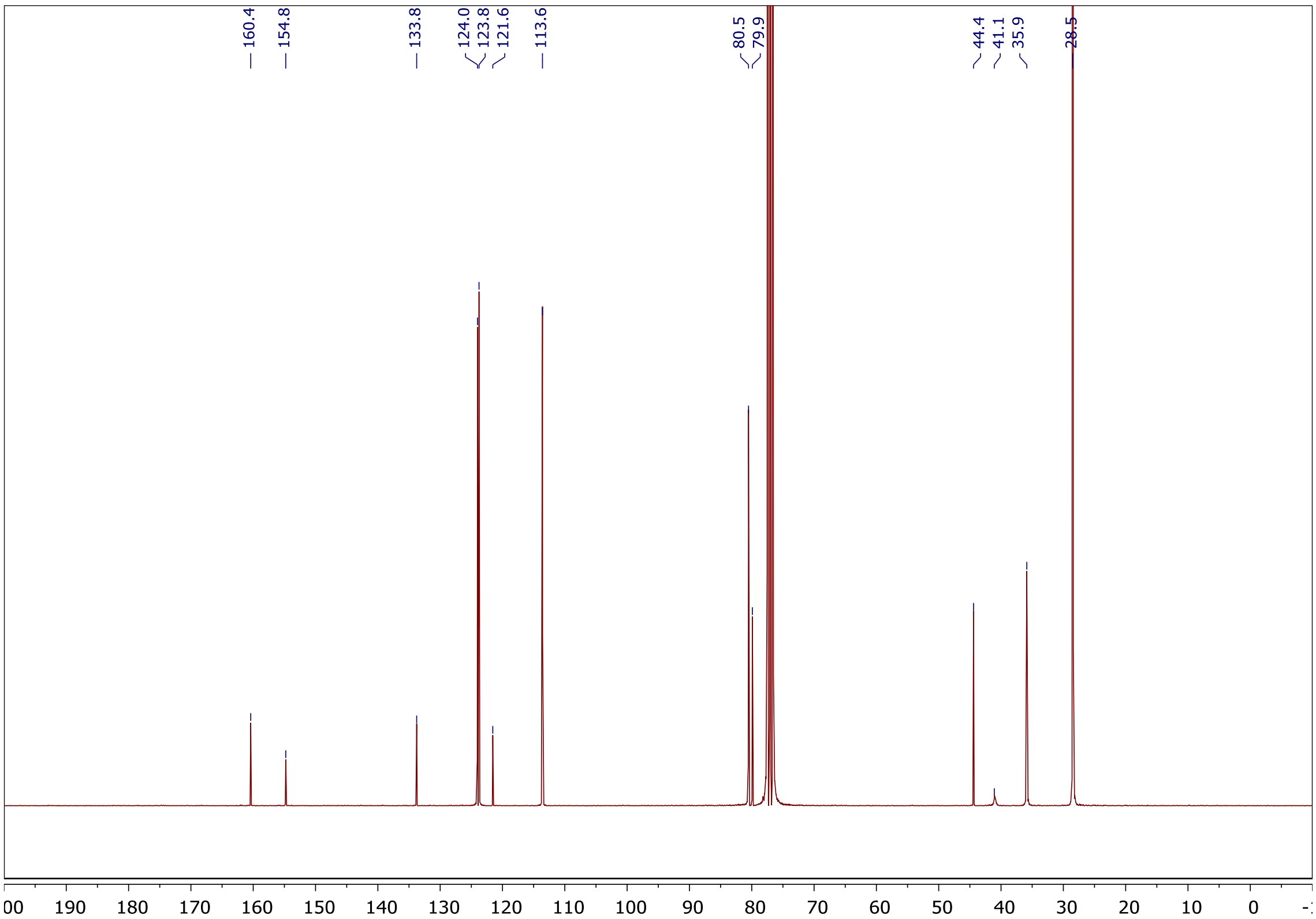

tert-Butyl 6-(3-chloro-4-[dimethylcarbamoyl]phenyl)-2H-spiro[benzofuran-3,4'-piperidine]-1'-carboxylate (14d)

^{1}H NMR (500 MHz, CD_2Cl_2)

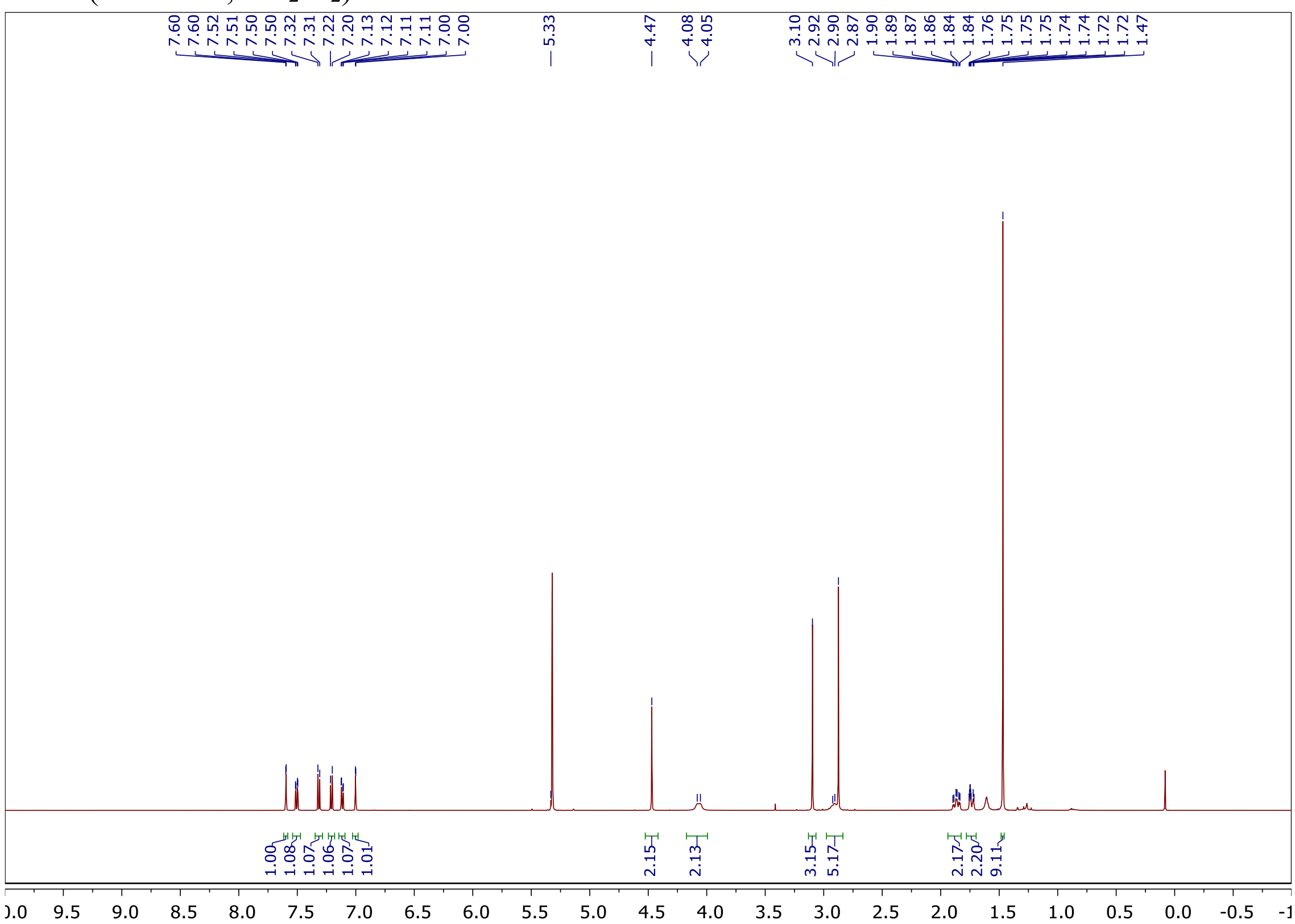

^{13}C NMR (126 MHz, CD_2Cl_2)

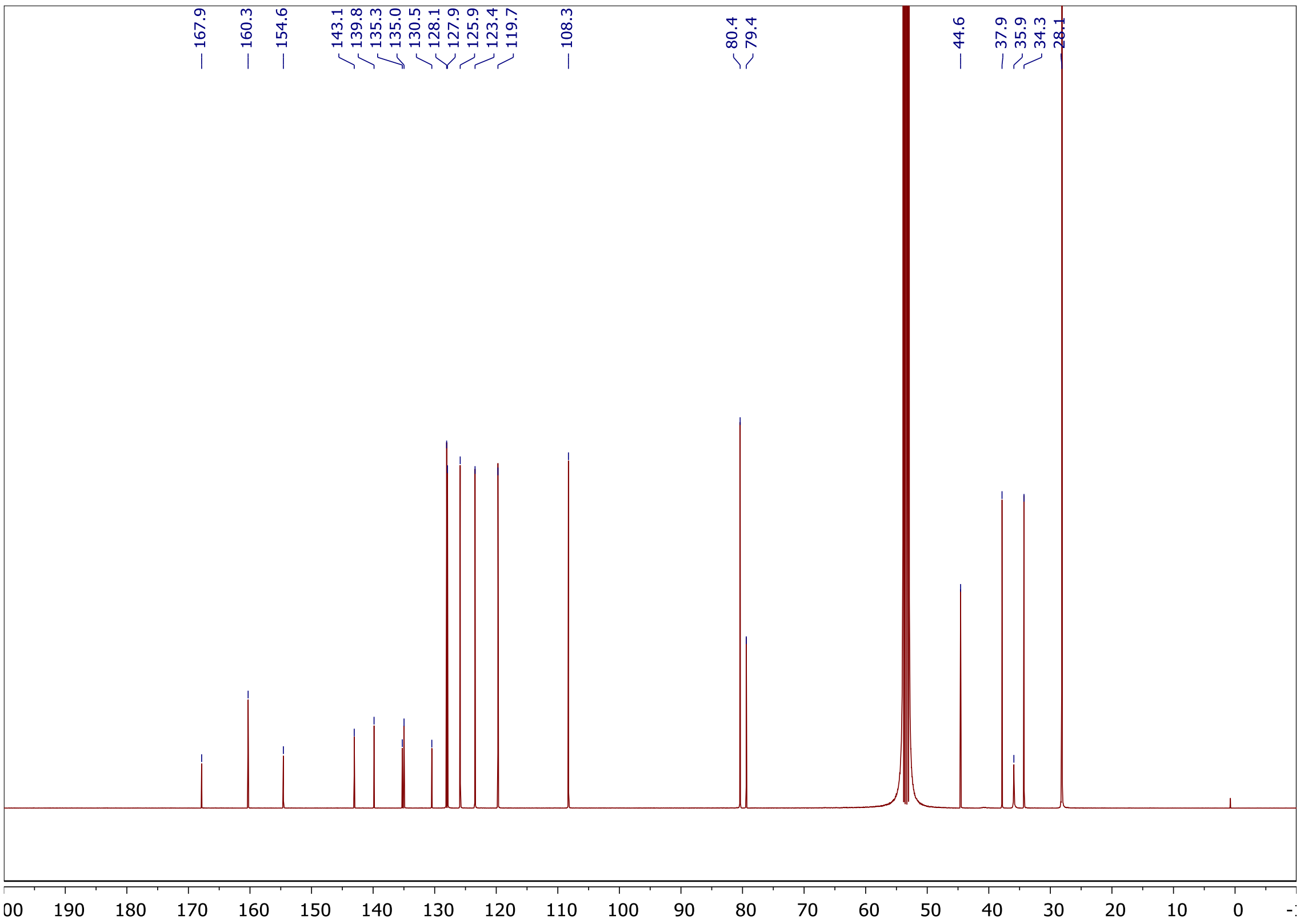

2-Chloro-*N,N*-dimethyl-4-(1'-[3-methyl-2-oxobutanoyl]-2*H*-spiro[benzofuran-3,4'-piperidin]-6-yl)benzamide (16d)

^{1}H NMR (400 MHz, $CDCl_3$)

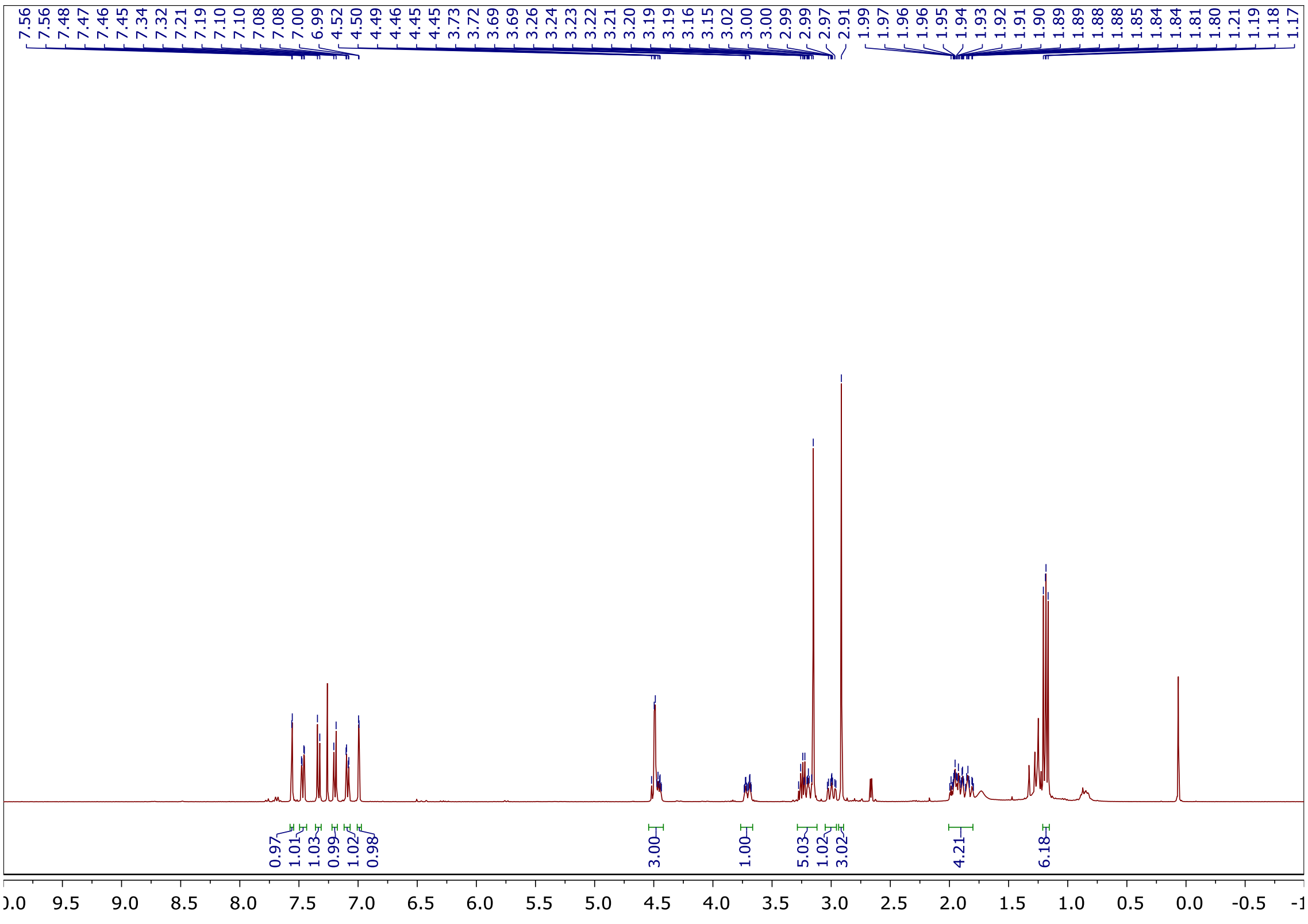

^{13}C NMR (101 MHz, $CDCl_3$)

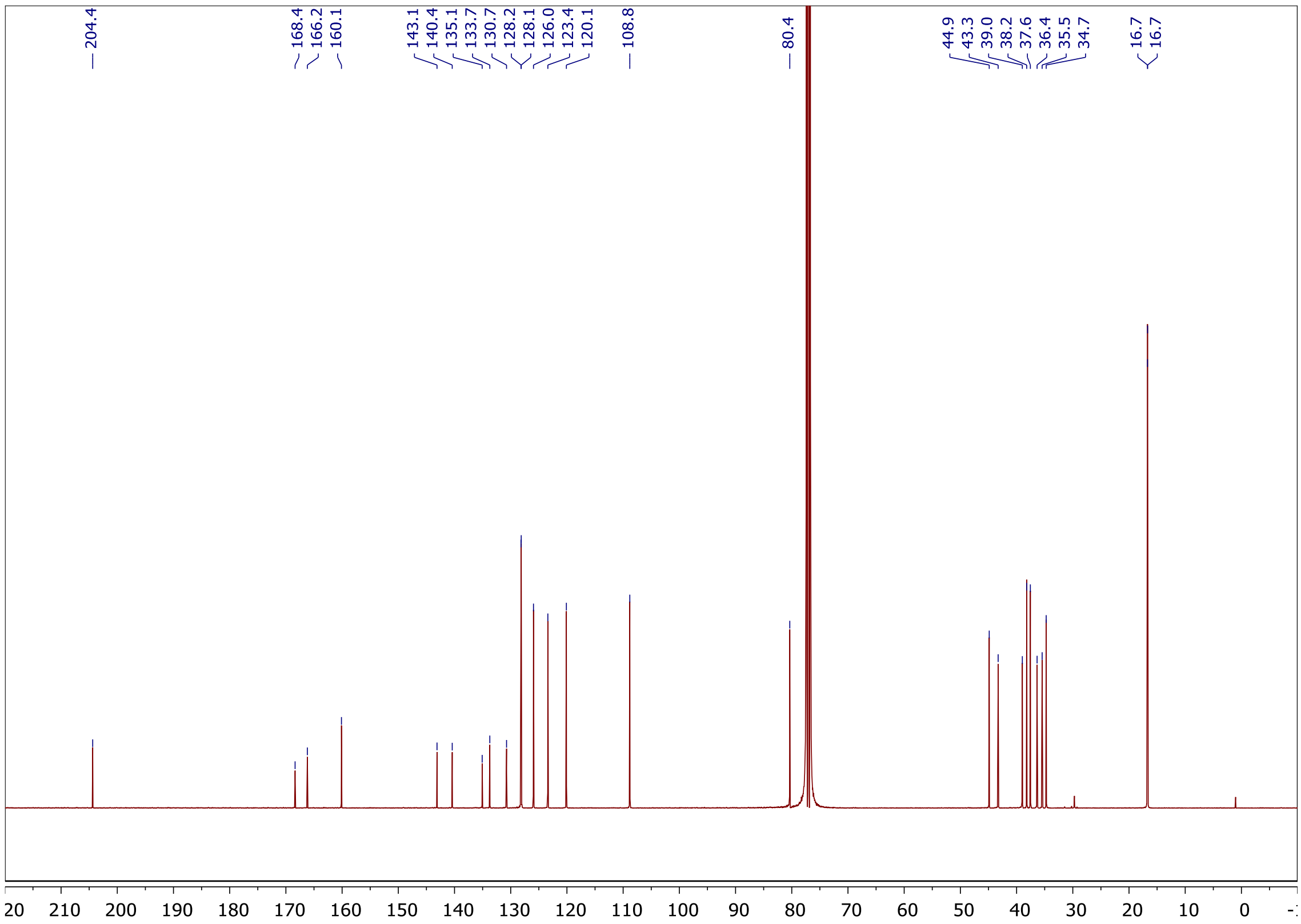

2-Chloro-4-(1'-[2-hydroxy-3-methyl-2-{trifluoromethyl}butanoyl]-2*H*-spiro[benzofuran-3,4'-piperidin]-6-yl)-*N,N*-dimethylbenzamide (*rac*-8)

^{1}H NMR (500 MHz, CD_2Cl_2)

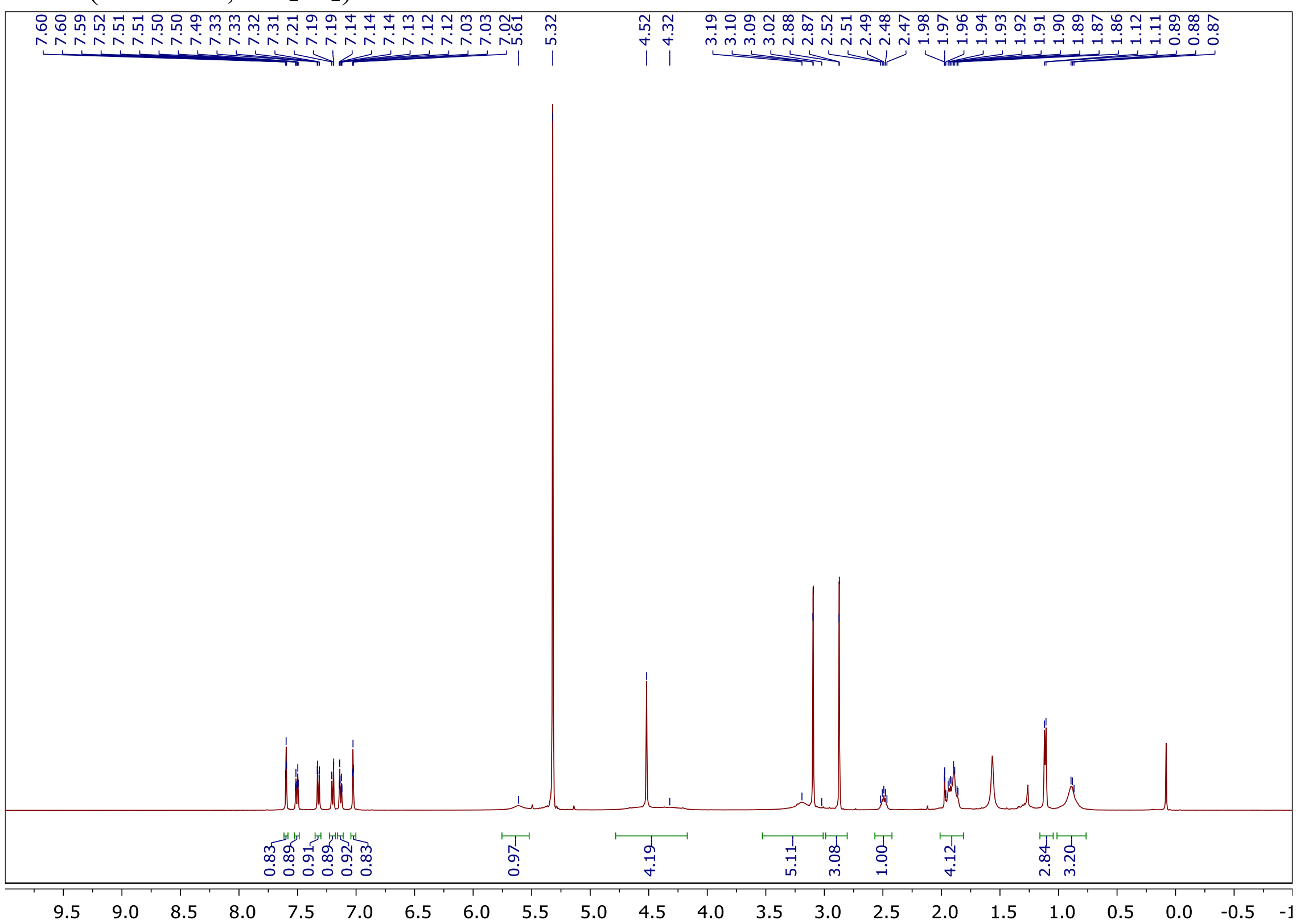

^{13}C NMR (126 MHz, CD_2Cl_2)

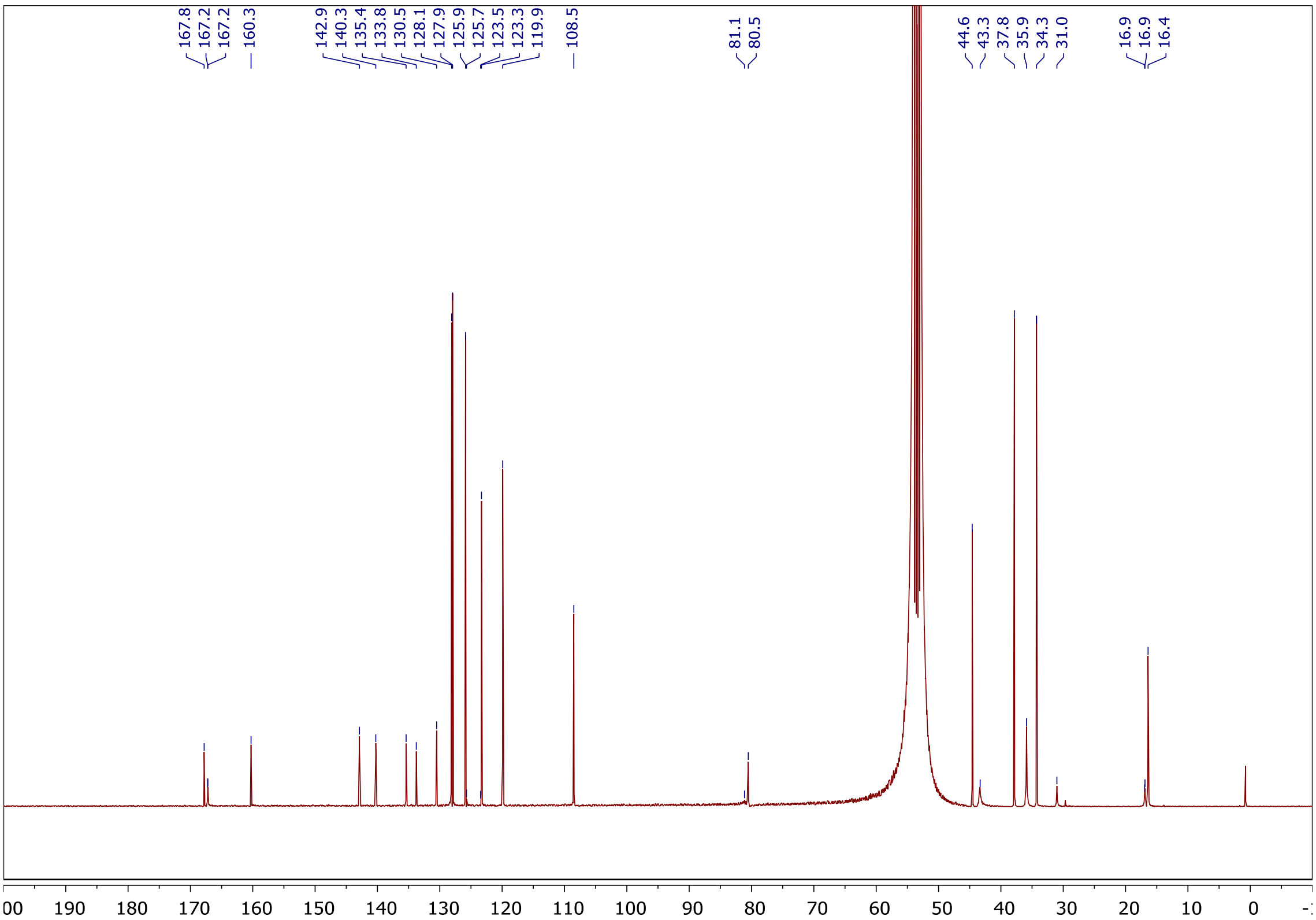

^{19}F NMR (471 MHz, CD_2Cl_2)

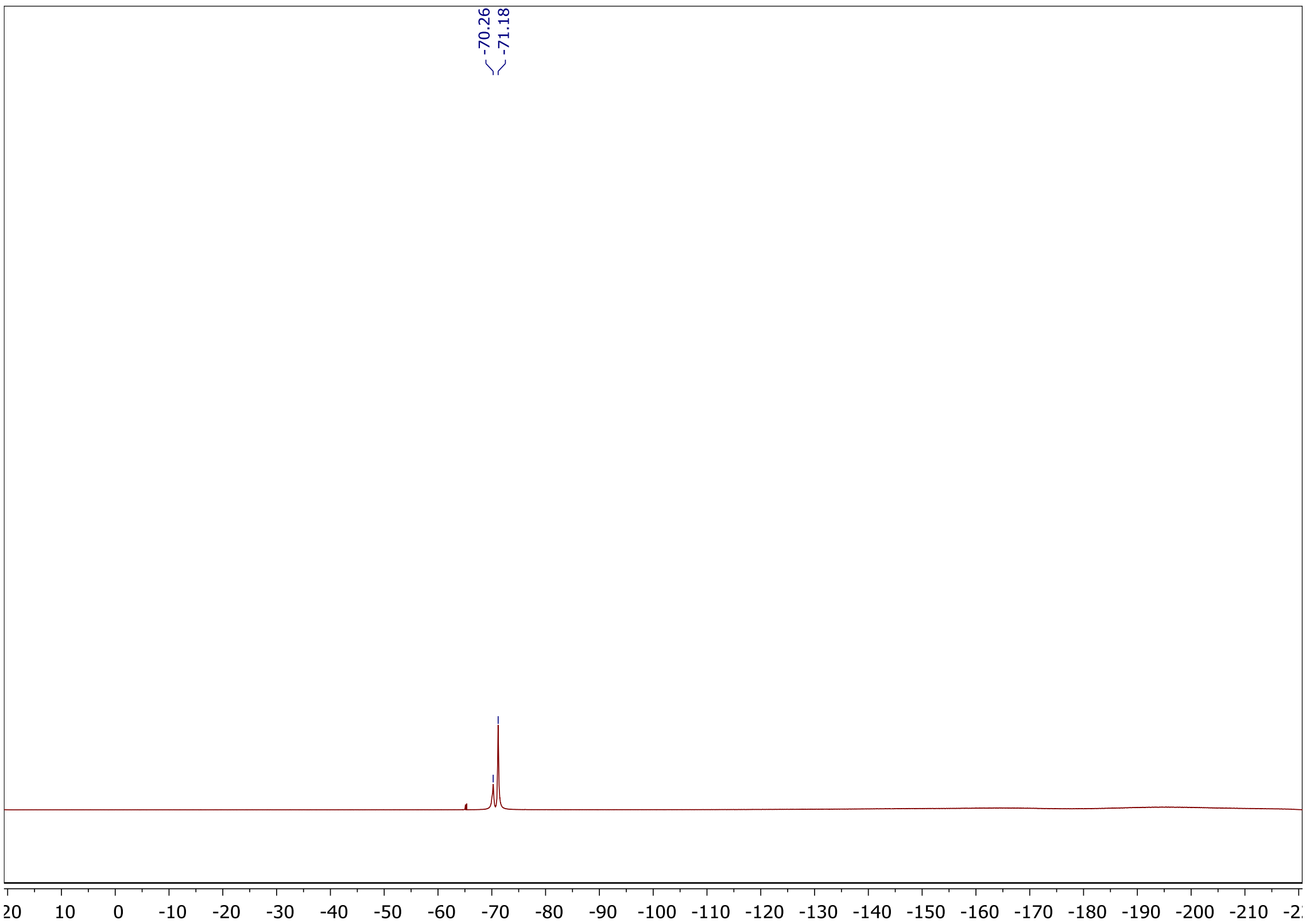

HPLC Traces

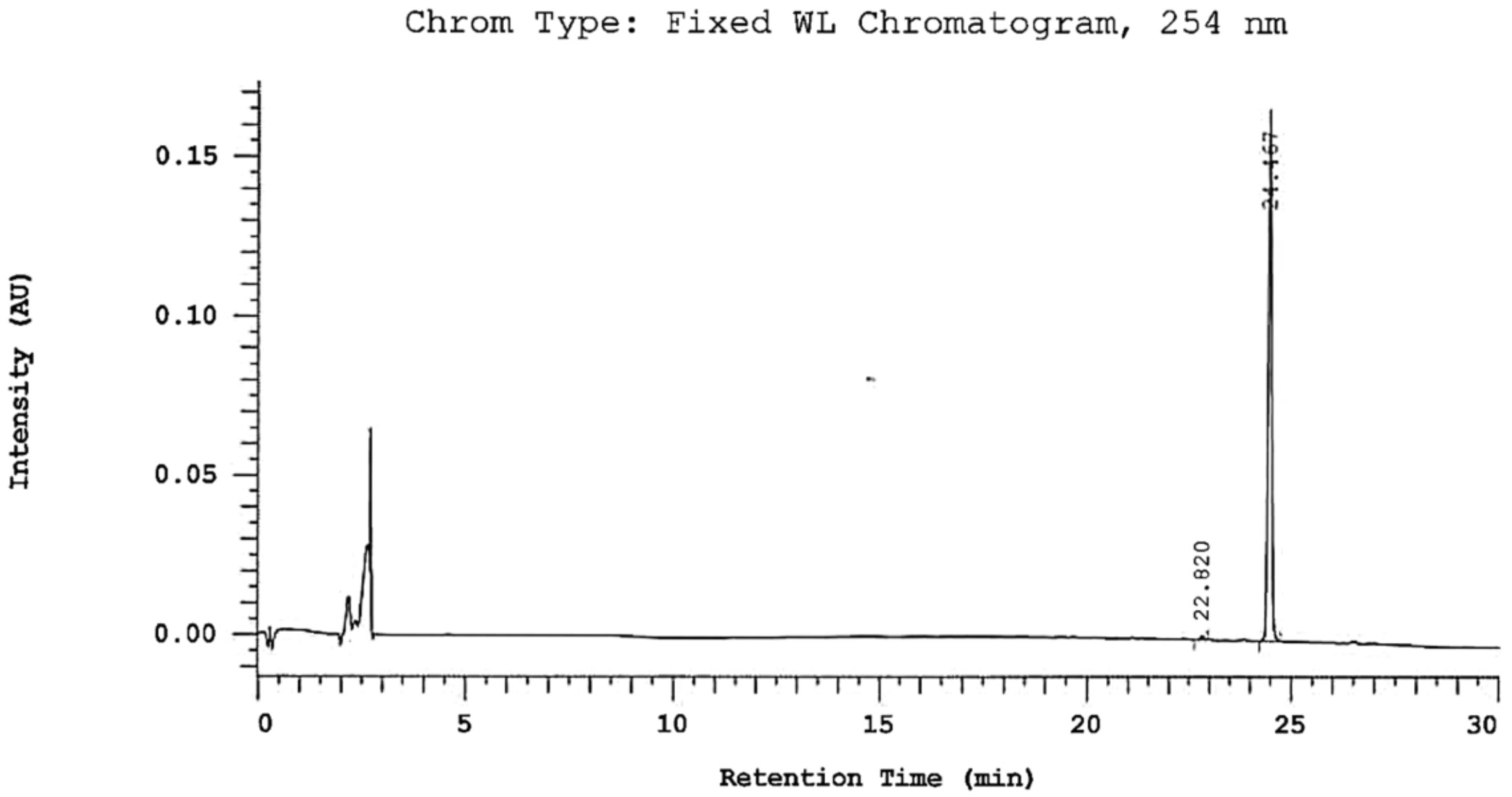

No.	RT	Area	Area %	Height
1	22.820	4055	0.860	638
2	24.467	467269	99.140	83325
		471324	100.000	83963

Peak rejection level: 0

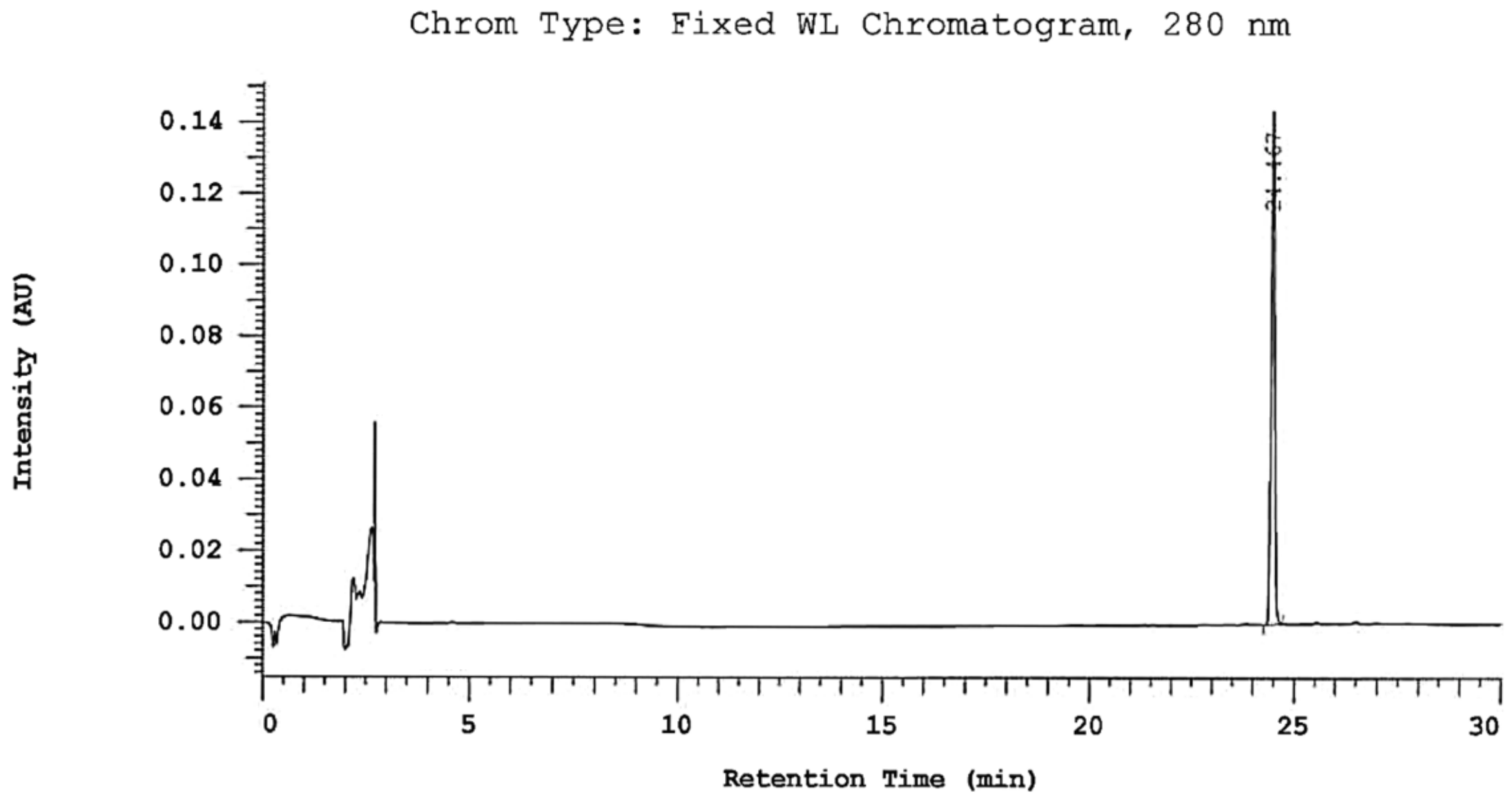

No.	RT	Area	Area %	Height
1	24.467	401149	100.000	71838
		401149	100.000	71838

Peak rejection level: 0

4. Supplementary References

(1) Stachel, S. J.; Zerbinatti, C.; Rudd, M. T.; Cosden, M.; Suon, S.; Nanda, K. K.; Wessner, K.; DiMuzio, J.; Maxwell, J.; Wu, Z.; Uslaner, J. M.; Michener, M. S.; Szczerba, P.; Brnardic, E.; Rada, V.; Kim, Y.; Meissner, R.; Wuelfing, P.; Yuan, Y.; Ballard, J.; Holahan, M.; Klein, D. J.; Lu, J.; Fradera, X.; Parthasarathy, G.; Uebele, V. N.; Chen, Z.; Li, Y.; Li, J.; Cooke, A. J.; Bennett, D. J.; Bilodeau, M. T.; Renger, J. Identification and in Vivo Evaluation of Liver X Receptor β-Selective Agonists for the Potential Treatment of Alzheimer's Disease. *J. Med. Chem.* **2016**, *59* (7), 3489–3498. https://doi.org/10.1021/acs.jmedchem.6b00176.

(2) Trott, O.; Olson, A. J. AutoDock Vina: Improving the Speed and Accuracy of Docking with a New Scoring Function, Efficient Optimization, and Multithreading. *J. Comput. Chem.* **2010**, *31* (2), 455–461. https://doi.org/10.1002/jcc.21334.

(3) Parsons, S.; Flack, H. D.; Wagner, T. Use of Intensity Quotients and Differences in Absolute Structure Refinement. *Acta Crystallogr. Sect. B Struct. Sci. Cryst. Eng. Mater.* **2013**, *69* (3), 249–259. https://doi.org/10.1107/S2052519213010014.

(4) Sheldrick, G. M. Crystal Structure Refinement with SHELXL. *Acta Crystallogr. Sect. C Struct. Chem.* **2015**, *71* (1), 3–8. https://doi.org/10.1107/S2053229614024218.

(5) Sheldrick, G. M. SHELXT – Integrated Space-Group and Crystal-Structure Determination. *Acta Crystallogr. Sect. A Found. Adv.* **2015**, *71* (1), 3–8. https://doi.org/10.1107/S2053273314026370.

(6) Lübben, J.; Wandtke, C. M.; Hübschle, C. B.; Ruf, M.; Sheldrick, G. M.; Dittrich, B. Aspherical Scattering Factors for SHELXL – Model, Implementation and Application. *Acta Crystallogr. Sect. A Found. Adv.* **2019**, *75* (1), 50–62. https://doi.org/10.1107/S2053273318013840.

(7) Speckmeier, E.; Fischer, T. G.; Zeitler, K. A Toolbox Approach To Construct Broadly Applicable Metal-Free Catalysts for Photoredox Chemistry: Deliberate Tuning of Redox Potentials and Importance of Halogens in Donor–Acceptor Cyanoarenes. *J. Am. Chem. Soc.* **2018**, *140* (45), 15353–15365. https://doi.org/10.1021/jacs.8b08933.
